# Integration of single-cell and bulk RNA-seq via machine learning to reveal ferroptosis- and lipid metabolism-driven immune landscape heterogeneity and predict immunotherapy response in colon cancer

**DOI:** 10.3389/fimmu.2025.1699079

**Published:** 2025-12-05

**Authors:** Weifeng Wang, Xuanhao Lin, Chufa Zheng, Peixiu Yao, Dejin Xie, Yiyan Lin, Xiaozhong Wang, Weiqin Hong

**Affiliations:** 1 Department of General Surgery, Shantou Central Hospital, Shantou, China; 2 Department of Biobank, Shantou Central Hospital, Shantou, China; 3 The Second Clinical School, Guangzhou Medical University, Guangzhou, China

**Keywords:** colon cancer, ferroptosis, immunotherapy, lipid metabolism, prognostic signature, single-cell analysis

## Abstract

**Objective:**

This study explored the interactions between ferroptosis and lipid metabolism in colon cancer, established a prognostic model to elucidate immune microenvironment heterogeneity, and evaluated the prospects of immunotherapy.

**Methods:**

Transcriptome sequencing and single-cell transcriptome data from The Cancer Genome Atlas and Gene Expression Omnibus were analyzed. Nonnegative matrix factorization clustering and weighted gene coexpression network analysis identified ferroptosis- and lipid metabolism-related genes. Machine learning algorithms including support vector machine, random forest, extreme gradient boosting, and least absolute shrinkage and selection operator regression were used to construct a prognostic model. Expression patterns of selected genes were validated via Human Protein Atlas and immunohistochemistry.

**Results:**

We developed a prognostic risk model comprising 13 genes through the application of multiple machine learning algorithm sand and confirmed as an independent prognostic factor. Gene set enrichment analysis (GSEA) revealed that the high-risk group was significantly enriched in hypoxia, tumor angiogenesis, epithelial-mesenchymal transition (EMT), and extracellular matrix (ECM) component synthesis and interactions, suggesting enhanced invasiveness and metastatic potential. Conversely, the low-risk group was enriched in biological processes related to oxidation, lipid metabolism, and ferroptosis. Moreover, the high-risk group exhibited more pronounced stromal infiltration and immunosuppressive activity within the tumor microenvironment, suggesting a greater tendency toward immune escape. In contrast, the low-risk group showed better responses to immunotherapy, a finding validated across multiple real-world immunotherapy datasets. Additionally, cell-cell communication analysis based on single-cell datasets revealed that M2 macrophages might be associated with T-cell exhaustion through SPP1-CD44 ligand-receptor interactions, thereby exerting immunosuppressive effects. Finally, immunohistochemistry (IHC) experiments confirmed the differential expression patterns of the SHH, WDR72, and EPOP genes between tumor and normal tissues, corroborating our findings at the mRNA level.

**Conclusion:**

In this study, we conducted a comprehensive analysis of ferroptosis-lipid metabolism interactions in colon cancer by integrating bulk transcriptomic and single-cell RNA sequencing data. The prognostic model constructed on the basis of lipid metabolism and ferroptosis-related genes has potential as an independent prognostic biomarker for colon cancer patients and may serve as a predictor of immunotherapy response, facilitating the optimization of personalized therapeutic strategies.

## 1 Introduction

According to global cancer statistics in 2020 (1), colon cancer (CC) ranks fifth in cancer incidence and fifth in cancer-related mortality worldwide. In 2020, approximately 1.15 million new CC cases (6.0%) and 580,000 CC-related deaths (5.8%) were reported globally (1). Although diverse therapeutic strategies for CC are currently available, surgery and adjuvant chemotherapy remain the primary treatment modalities (2). The initial symptoms of CC are not prominent, and statistical data indicate that approximately 20% of newly diagnosed colorectal cancer patients already present with metastases at diagnosis (3). Additionally, owing to heterogeneity in the tumor microenvironment, some patients exhibit limited sensitivity to chemotherapy. It has been reported that more than 40% of patients are resistant to 5-fluorouracil (5-FU), with some experiencing drug resistance and disease recurrence even after treatment (4). Therefore, there is an urgent need to establish novel gene models capable of assisting in disease diagnosis, assessing patient prognosis, dissecting the tumor microenvironment, and providing personalized therapeutic options for cancer treatment.

Ferroptosis is a newly defined form of regulated cell death (RCD) characterized by iron overload, accumulation of lipid reactive oxygen species (ROS), and lipid peroxidation (5). Morphologically, biochemically, and genetically, ferroptosis differs distinctly from apoptosis, necrosis, autophagy, and other forms of RCD (6). Previous studies (7, 8)have demonstrated a close association between lipid metabolism and cellular sensitivity to ferroptosis. Specifically, monounsaturated fatty acids (MUFAs) and polyunsaturated fatty acids (PUFAs) dictate the degree of intracellular lipid peroxidation and susceptibility to ferroptosis (9). For example, SCD1, a key enzyme involved in lipid metabolism, catalyzes the rate-limiting step of MUFA synthesis and protects ovarian cancer cells from ferroptosis (10). Reducing the intake of polyunsaturated fatty acids can decrease the accumulation of lipid peroxidation substrates within cells, thereby inhibiting ferroptosis (11). Similarly, Yang et al. (12) reported that BRD4 and HMGB2 upregulated the transcription levels of genes involved in fatty acid β-oxidation and PUFA synthesis (such as HADH, ACSL1, and ACAA2), thereby promoting erastin-induced ferroptosis. Therefore, lipid metabolism plays a critical role in determining cellular sensitivity to ferroptosis. Ferroptosis and lipid metabolism may cooperatively regulate multiple cancer-related processes, including carcinogenesis, progression, drug resistance, metastasis, and tumor immunity, thus providing various potential therapeutic strategies for cancer treatment. Consequently, further exploration of the interplay between ferroptosis and lipid metabolism from a multiomics perspective is warranted to elucidate the molecular mechanisms underlying their roles in colon cancer progression.

Immunotherapy has demonstrated substantial potential in improving cancer prognosis, among which T lymphocyte-based tumor immunotherapy has become an effective tool for cancer treatment (13). Promising strategies such as anti-PD-1, anti-PD-L1, anti-CTLA4, adoptive cell therapy (ACT), and chimeric antigen receptor (CAR) T-cell therapy are emerging as valuable approaches for treating colorectal cancer (CRC) (14). Notably, CRC patients with mismatch repair deficiency (dMMR) or high microsatellite instability (MSI-H) have shown remarkable therapeutic responses to anti-PD-1 therapies (15). Recent research (16) has indicated that immunotherapy-activated CD8+ T cells enhance ferroptosis in tumor cells, with increased ferroptosis contributing to the efficacy of antitumor immune therapies. Gui (17) et al. demonstrated that Ononin induces ferroptosis in colorectal cancer cells by increasing lipid ROS and downregulating GPX4 expression via the PI3K/AKT/Nrf2 pathway. Combination therapy with Ononin and anti–PD-L1 treatment exerts a synergistic antitumor effect, effectively inhibiting tumor growth and enhancing immune responses. However, other studies (18) revealed that CD36 could regulate fatty acid uptake in CD8+ T cells, thereby inducing lipid peroxidation and ferroptosis within CTLs, ultimately impairing cytokine production and antitumor efficacy. Thus, the roles of ferroptosis and lipid metabolism in colon cancer immunotherapy involve highly complex regulatory mechanisms, which require further in-depth investigation.

## 2 Methods

### 2.1 Collection and preprocessing of transcriptomic datasets

To address the imbalance between tumor and normal samples in the TCGA-COAD dataset, we utilized the TCGA-GTEx colon cancer cohort (344 normal samples and 289 tumor samples) from the UCSC Xena website (https://xenabrowser.net/) for differential expression analysis. mRNA-seq data and corresponding survival information for prognostic analysis were also obtained from UCSC Xena. Additionally, partial microarray datasets were retrieved from the Gene Expression Omnibus (GEO) database (http://www.ncbi.nlm.nih.gov/geo/). To ensure the quality of the RNA-seq data and clinical prognosis information, the following criteria were applied: (1) In gene expression matrices, genes were expressed in at least 30% of the samples (2); For survival analysis, only one sample per patient was retained, typically primary fresh-frozen tissues; (3) Samples with incomplete clinical information or survival times of less than 30 days were excluded. Ultimately, the TCGA-COAD training cohort included 371 colon cancer patients, whereas the validation cohorts included 477 (GSE39582), 144 (GSE17536), 232 (GSE17538), 124 (GSE72970), and 184 (GSE87211) colorectal cancer patients. Gene expression levels were normalized to transcripts per million (TPM) values and log2-transformed.

### 2.2 Identification of FRGs and LMGs

FerrDb (19) (http://www.zhounan.org/ferrdb/) is the first database dedicated to ferroptosis regulators and their associations with ferroptosis-related diseases. GeneCards (20) (version 5.18; https://www.genecards.org/) is a comprehensive database providing integrated information on all annotated and predicted human genes. FerrDb yielded 728 ferroptosis-related genes and GeneCards (relevance score > 1) provided 835 additional candidates; after merging and removing duplicates, 1093 FRGs were retained (**Supplementary File 1**). For lipid metabolism, Molecular Signatures Database (MSigDB, http://gsea-msigdb.org) (21) and relevant literature (22–24) identified 1164 genes, and GeneCards (relevance score > 10) contributed 1027; deduplication resulted in 1765 unique LMGs (**Supplementary File 2**).

### 2.3 Nonnegative matrix factorization clustering analysis

We performed nonnegative matrix factorization (NMF) clustering analysis to develop molecular subtypes on the basis of the expression profiles of 56 differentially expressed ferroptosis- and lipid metabolism-related genes (FRLMRGs) (**Supplementary File 3**). For the NMF method, the standard “brunet” algorithm was employed with 20 iterative runs. The number of clusters was evaluated from 2 to 10, and the minimum number of samples per subclass was set to 10. In addition, to evaluate resampling stability, we compared the consistency of sample cluster assignments across different random seeds. Five independent NMF runs were conducted using five distinct random seeds, each with 20 internal initializations, and pairwise adjusted Rand indices (ARI) were computed among the runs. The mean ARI at k = 3 was 0.8905, indicating excellent reproducibility across different seeds and independent initializations, thus confirming the robustness of the identified molecular subtype classification (−1 ≤ ARI ≤ 1; ARI = 1 represents identical partitions, ARI = 0 indicates random agreement). Consensus matrix, cophenetic coefficient, mean silhouette width, and the manually computed CDF/ΔCDF curves were generated to further validate the stability and distinctiveness of the identified clusters.

### 2.4 Gene set variation analysis

To explore the biological differences among various subgroups, we performed gene set variation analysis (GSVA) to quantify the differential activity scores of various gene pathways across the identified clusters. The nine ferroptosis- and lipid metabolism–related pathways analyzed in this study were obtained from the R package “msigdbr” (version 7.5.1), and the complete gene lists for these pathways have been provided in the **Supplementary File 4**. For comparisons across multiple pathways, we applied the Wilcoxon rank-sum test to evaluate differences in GSVA enrichment scores. All P values were adjusted using the Benjamini–Hochberg false discovery rate (FDR) correction to account for multiple pathway comparisons, and both the Cliff’s delta (δ) and the FDR-adjusted P values were reported to ensure statistical rigor. Ten tumor-associated pathways (Cell cycle, Hippo, MYC, Notch, Nrf2, PI3K_AKT, RTK_RAS, TGF-β, P53, and Wnt) were obtained from the Molecular Signatures Database (MSigDB) (25, 26). Furthermore, we assessed the correlations between risk scores and six specific pathways: HYPOXIA, TUMOR_ANGIOGENESIS, VEGF_VEGFR, CHEMOTAXIS, and PYROPTOSIS, which were also obtained from MSigDB, and STEMNESS, which was derived from datasets quantifying tumor sample stemness indices reported in previous studies (27, 28).

### 2.5 Weighted gene coexpression network analysis

We conducted weighted gene coexpression network analysis (WGCNA) to identify trait-associated gene modules. The median absolute deviation (MAD) was utilized for gene filtering to retain genes with substantial variability and potential biological significance, and the top 8000 genes were selected according to their MAD scores. The minimum number of genes per module was set to 80, facilitating the construction of the WGCNA network. Scale-free topology and mean connectivity analyses were conducted across different power values, followed by construction of the topological overlap matrix (TOM) and dissimilarity matrix (1-TOM). Correlations between gene modules and traits were then evaluated.

### 2.6 Derivation of the FRLM-related prognostic signature

To identify ferroptosis– and lipid metabolism–related prognostic biomarkers and construct a robust prognostic model, we applied an integrated strategy combining machine learning–based feature selection with LASSO and stepwise Cox regression analyses.

#### 2.6.1 Feature selection using multiple machine learning algorithms

Candidate prognostic genes were initially screened using three independent machine learning algorithms: Random Forest (RF), Support Vector Machine (SVM), and Extreme Gradient Boosting (XGBoost). Samples were randomly partitioned into training (70%) and testing (30%) cohorts using the createDataPartition function from the caret R package. Model performance was evaluated using 10-fold cross-validation (trainControl(method = “repeatedcv”, number = 10)).

For the RF model (method = “rf”), a parameter grid search was performed with mtry = {2, 4, 6, 8}, followed by tuning of the number of decision trees (ntree = {100, 200, 300, 500, 700}). The optimal parameters were mtry = 4 and ntree = 700. For the SVM model with a radial basis function kernel (method = “svmRadial”), the penalty parameter C and kernel width sigma were optimized with a grid search (C = {0.1, 1, 10} and sigma = {0.01, 0.1, 1}), and the final model used C = 1 and sigma = 0.01. For the XGBoost model (method = “xgbTree”), the best parameters were determined as nrounds = 200, max_depth = 5, eta = 0.1, gamma = 0, colsample_bytree = 1, min_child_weight = 1, and subsample = 1.

Feature importance was calculated for each algorithm using the DALEX package (variable_importance function), and the top-ranked genes across the three methods were integrated to generate the candidate gene set for subsequent analyses.

#### 2.6.2 Dimensionality reduction using LASSO-Cox regression

The candidate genes were further refined using least absolute shrinkage and selection operator (LASSO) Cox regression implemented in the glmnet R package. A 10-fold cross-validation procedure (cv.glmnet function) was used to optimize the regularization parameter λ. The optimal λ was determined based on the minimum partial likelihood deviance (lambda.min = 0.01528725, log λ ≈ −4.18). Only genes with nonzero regression coefficients were retained, yielding 24 prognostic genes.

#### 2.6.3 Final model construction using multivariate stepwise cox regression

Multivariate Cox proportional hazards regression analysis was then performed on the 24 LASSO-selected genes using the coxph function from the survival package. Bidirectional stepwise selection based on Akaike information criterion (AIC) was applied (step function, direction = “both”) to determine the optimal gene set. The final prognostic model consisted of 13 core genes, and the risk score for each patient was calculated as the sum of each gene’s expression level multiplied by its corresponding Cox regression coefficient.

### 2.7 Classification based on consensus molecular subtypes

The consensus molecular subtypes (CMS) classification, proposed by the Colorectal Cancer Subtyping Consortium (CRCSC) in 2015 (29), stratifies CRC into four subtypes (CMS1–CMS4) on the basis of multiomics data. In this study, tumor samples from 371 TCGA-COAD patients were classified into CMS subtypes via the CMScaller R package (version 2.0.1) (30), which employs a template-based algorithm with permutation testing (nPerm = 1000) and FDR control (FDR = 0.05). Samples (n=36, 9.70%) that failed to meet the FDR threshold were excluded. A Sankey diagram illustrating the CMS subtype distribution was generated via an online platform (https://www.bioinformatics.com.cn, accessed on 7 Jan 2025).

### 2.8 Real-world immunotherapy cohort datasets

We obtained real-world immunotherapy data from metastatic urothelial carcinoma patients treated with anti-PD-L1 therapy via the IMvigor210CoreBiologies R package (http://research-pub.gene.com/IMvigor210CoreBiologies) (31). In addition, five immunotherapy cohorts with varying therapeutic responses were downloaded from the TIGER database (http://tiger.canceromics.org/), including Melanoma-GSE78220 (melanoma treated with anti-PD-1), Melanoma-GSE91061 (melanoma treated with anti-PD-1), Melanoma-GSE100797 (melanoma treated with adoptive cell therapy [ACT]), Melanoma-PRJEB23709 (melanoma treated with anti-PD-1 combined with anti-CTLA4), and STAD-PRJEB25780 (gastric adenocarcinoma treated with anti-PD-1). Additionally, we retrieved the GSE35640 cohort from the GEO database, which includes metastatic melanoma patients who received MAGE-A3 immunotherapy (32).

### 2.9 Analysis of single-cell sequencing data

#### 2.9.1 Single-cell data quality control and processing

The 10x Genomics single-cell RNA-seq dataset of colon cancer, GSE132465 (from Samsung Medical Center [SMC]), was downloaded from the GEO database (https://www.ncbi.nlm.nih.gov/geo/), from which 19 colon cancer samples were selected. The Seurat R package (version 4.4.2) was used to create Seurat objects. The datasets were filtered using the following criteria: 200 < unique feature counts (nFeature_RNA) < 6000; each gene must be expressed in at least 3 cells; and mitochondrial gene expression accounting for <15% of total unique molecular identifier counts. After quality control, 36,590 cells were retained.

The NormalizeData function was used to normalize the raw data, and 2,000 highly variable genes were selected via the FindVariableFeatures function. The data were scaled via the ScaleData function, and principal component analysis (PCA) was performed via the RunPCA function, with 15 principal components selected. Batch effects among samples were corrected via the RunHarmony function. Cell clustering was performed via the FindClusters function and a resolution of 0.8 was ultimately used for clustering. To reduce dimensionality effectively and visualize the data, we performed t-distributed stochastic neighbor embedding (t-SNE) analysis via the RunTSNE function. We subsequently identified marker genes for each cluster via the FindAllMarkers function. Cell type annotation was conducted on the basis of reference annotation files from online resources such as CellMarker (http://biocc.hrbmu.edu.cn/CellMarker) and PanglaoDB (https://panglaodb.se/), as well as with the assistance of the SingleR package (version 2.8.0). The DimPlot function was used to visualize cell clusters and annotated cell types, whereas the DoHeatmap function was applied to generate expression heatmaps for selected cells and features. Copy number variations (CNVs) and the identification of aneuploid tumor cells were analyzed via the copykat R package.

#### 2.9.2 Scoring of M1 and M2 macrophages and the FRLM score (FRLM, ferroptosis and lipid metabolism)

The M1 macrophage-related signature gene set included: CD80, TNF, CD86, IL1B, IL6, TLR4, IRF1, and CCR7. The M2 macrophage-related signature gene set included: CCL22, TGFB1, MARCO, CCL18, CTSA, MMP9, CD163, and TGM2. The ferroptosis–lipid metabolism-related signature genes (FRLMRGs) are listed in **Supplementary File 3**. We used the AUCell R package (version 1.28.0) to calculate gene set activity scores.

### 2.10 Molecular docking

We performed protein–protein docking via the GRAMM online tool (https://gramm.compbio.ku.edu/) (33, 34), which performs rigid-body docking, meaning that the shapes of the ligand and receptor remain unchanged while potential binding sites are identified on the protein surfaces. On the basis of the genes SPP1 and CD44, we retrieved the corresponding protein sequences from the UniProtKB online database (https://www.uniprot.org/uniprotkb), and submitted the sequences to the SWISS-MODEL web tool (https://swissmodel.expasy.org/) to obtain the optimal 3D protein structures. The resulting PDB files were then used for protein–protein docking. A total of 10 docking results were generated. In general, a lower binding free energy indicates a more stable complex and better binding affinity; thus, we selected the result with the lowest binding free energy for visualization.

### 2.11 Immunohistochemistry

To validate the findings of this study, we collected 30 tumor tissue samples and 30 adjacent normal tissue samples from our institution for immunohistochemistry (IHC). The antibodies used included anti-SHH (YT6188, Immunoway), anti-EPOP (ab247125, Abcam), and anti-WDR72 (ab190268, Abcam) antibodies. The experimental procedure was as follows: Colon cancer and adjacent normal tissue sections were deparaffinized in xylene and rehydrated through a graded ethanol series in water. Antigen retrieval was performed via heat-induced epitope retrieval in EDTA buffer (1:50 dilution) (boiling method). Endogenous peroxidase activity was blocked by the addition of a peroxidase inhibitor to reduce nonspecific staining. After blocking with normal goat serum, the slides were incubated overnight at 4°C with the following primary antibodies: anti-SHH (1:100), anti-EPOP (1:500), and anti-WDR72 (1:1000). The sections were subsequently incubated with the secondary antibody at 37°C for 1 hour and developed with DAB solution for 30 seconds, followed by hematoxylin counterstaining. Finally, the slides were dehydrated with absolute ethanol and xylene and mounted. SHH was expressed mainly in the nucleus and cytoplasm, whereas WDR72 and EPOP were expressed primarily in the nucleus. Semi-quantitative evaluation of target protein expression was performed via the IHC score (the percentage of positive cells*the staining intensity) via ImageJ software (version v1.54.0, NIH, Bethesda, USA). For each sample, five random high-power fields (400× magnification) were selected, and the IHC scores were calculated and averaged. The scoring criteria for the percentage of positive cells were as follows: 0 points (0%–5%), 1 point (6%–25%), 2 points (26%–50%), 3 points (51%–75%), and 4 points (>76%). The staining intensity was scored as follows: 0 points (negative), 1 point (weakly positive), 2 points (positive), and 3 points (strongly positive).

### 2.12 Statistical analysis

All the statistical analyses were performed via R software (version 4.4.2, https://www.r-project.org/, accessed on October 31, 2024) and its associated packages. The Wilcoxon test was used to compare differences between two groups, whereas the Kruskal–Wallis test was applied for comparisons among multiple groups. For pairwise comparisons within multiple groups, p values were adjusted via the Benjamini–Hochberg method to control for false positives arising from multiple hypothesis testing. Spearman correlation analysis was used to determine correlation coefficients. Kaplan-Meier (K-M) survival curves were generated, and the log-rank test was used to evaluate differences in survival between groups. In this study, a two-sided p value < 0.05 was considered statistically significant. Statistical significance was annotated as follows: **** for p < 0.0001, *** for p < 0.001, ** for p < 0.01, and * for p < 0.05.

## 3 Results

### 3.1 Overall study flowchart

**Figure 1:**
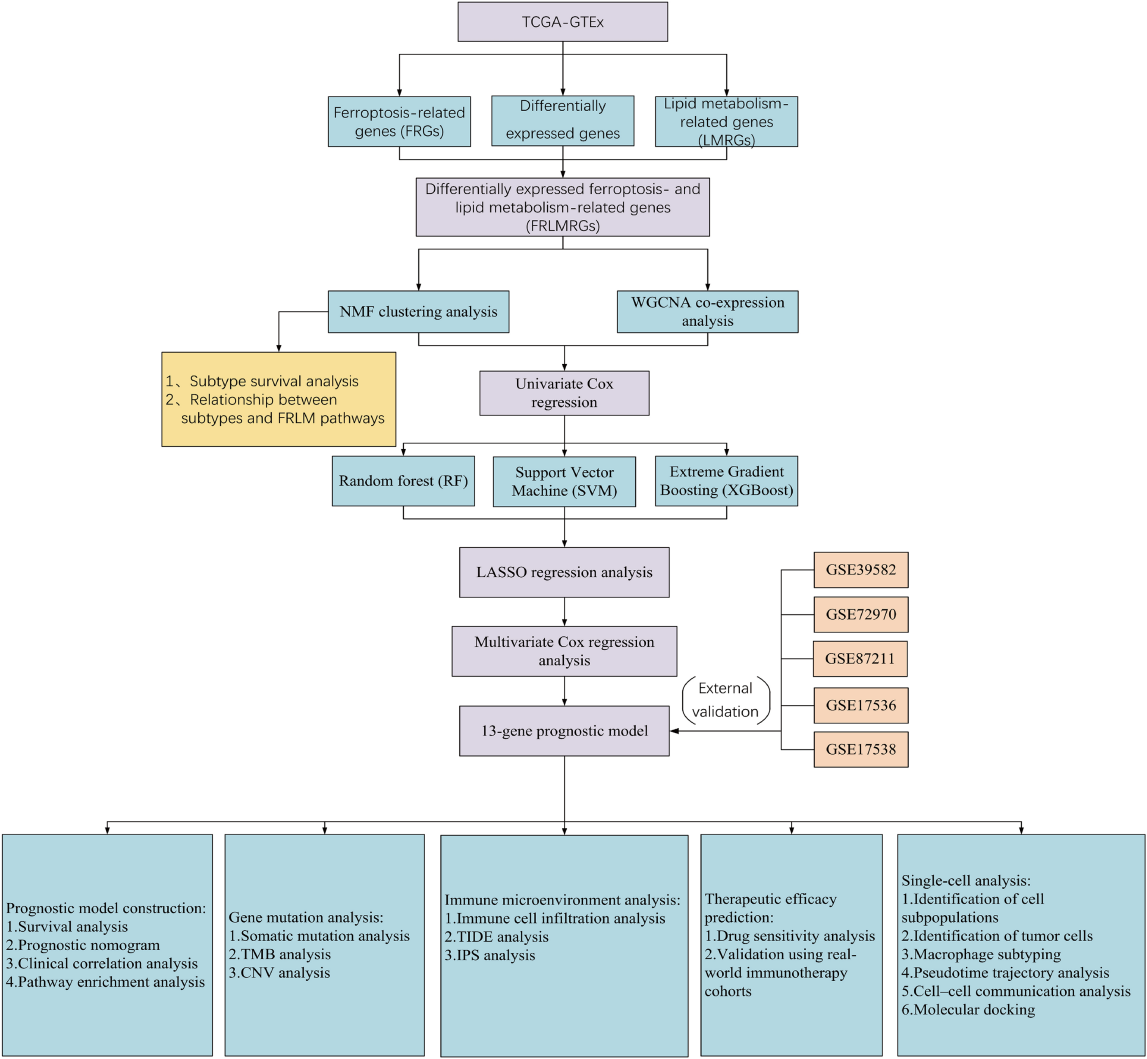
Flowchart showing the overall design and analytic procedure of this study.

An analysis flow chart of our bioinformatics workflow is shown in **Figure 1**.

### 3.2 Acquisition of ferroptosis-lipid metabolism related genes

**Figure 2:**
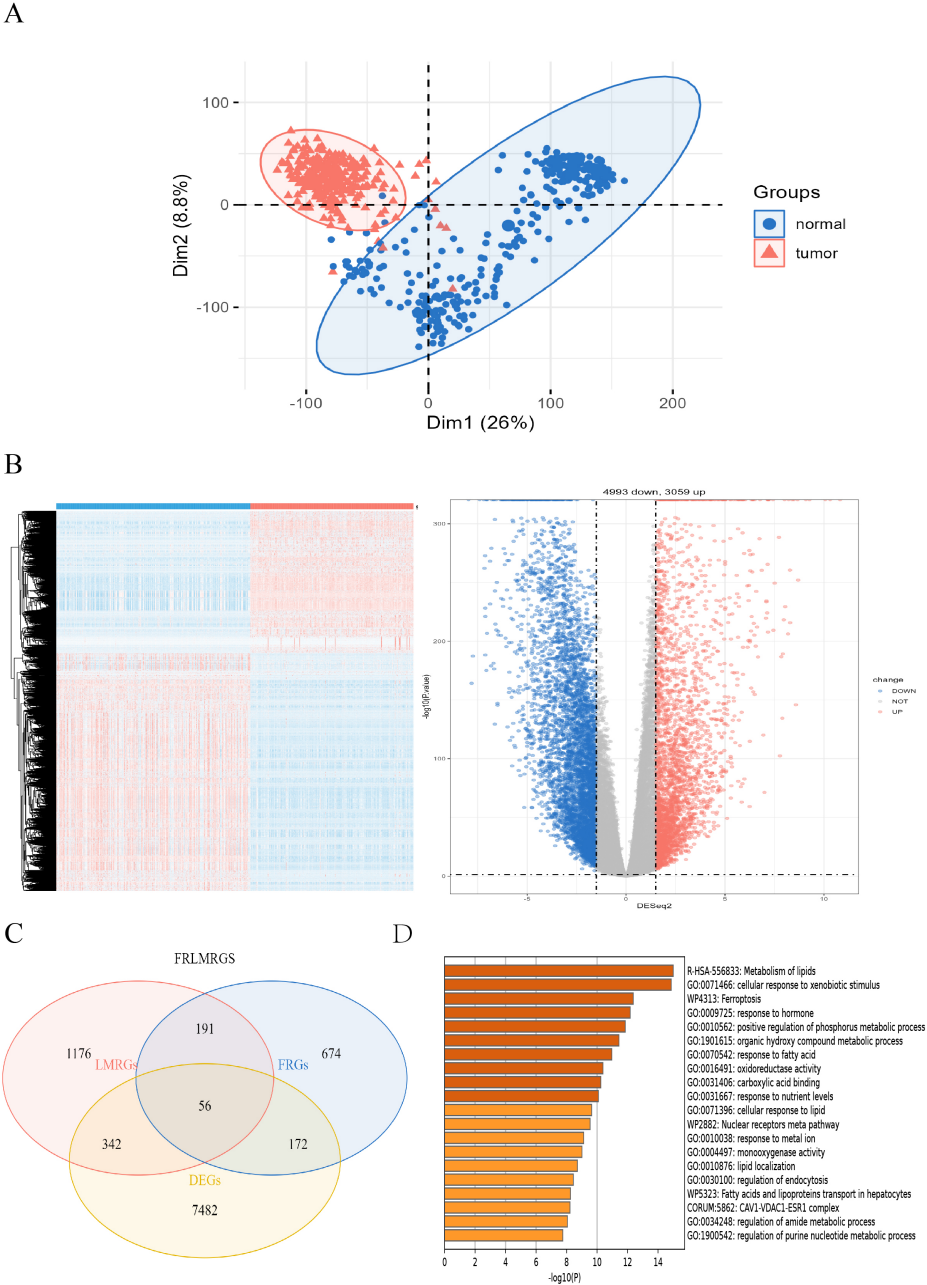
Sources of FRLMRGs. **(A)** PCA scatter plot showing the distribution of tumor and normal samples after dimensionality reduction. **(B)** Heatmap and volcano plot illustrating differential gene expression, with red indicating upregulation and blue indicating downregulation in tumors. **(C)** Venn diagram demonstrating the intersection of LMRGs, FRGs, and DEGs, identifying 56 overlapping genes defined as FRLMRGs. **(D)** Bar chart of the results of the Metascape enrichment analysis showing significant enrichment of FRLMRGs in lipid metabolism and ferroptosis.

Principal component analysis (PCA) of the TCGA-GTEx colon cancer dataset (normal samples: 344; tumor samples: 289) revealed a clear separation between tumor and normal tissues (**Figure 2A**). Differential expression analysis via the DESeq2 package(FDR = 0.05, |log2FC| > 1.5) identified 8,052 differentially expressed genes (DEGs) (**Supplementary Data 1**), which were visualized via volcano plots and heatmaps (**Figure 2B**). The intersection of these DEGs with 1,093 ferroptosis-related genes (FRGs) and 1,765 lipid metabolism-related genes (LMRGs) yielded 56 differentially expressed ferroptosis- and lipid metabolism-related genes (FRLMRGs) (**Supplementary File 3**), as shown in the Venn diagram (**Figure 2C**). Metascape enrichment analysis revealed that these 56 genes were predominantly involved in lipid metabolism and ferroptosis pathways, ranking first and third among the top enriched terms (**Figure 2D**), confirming their dual association.

### 3.3 NMF clustering analysis

**Figure 3:**
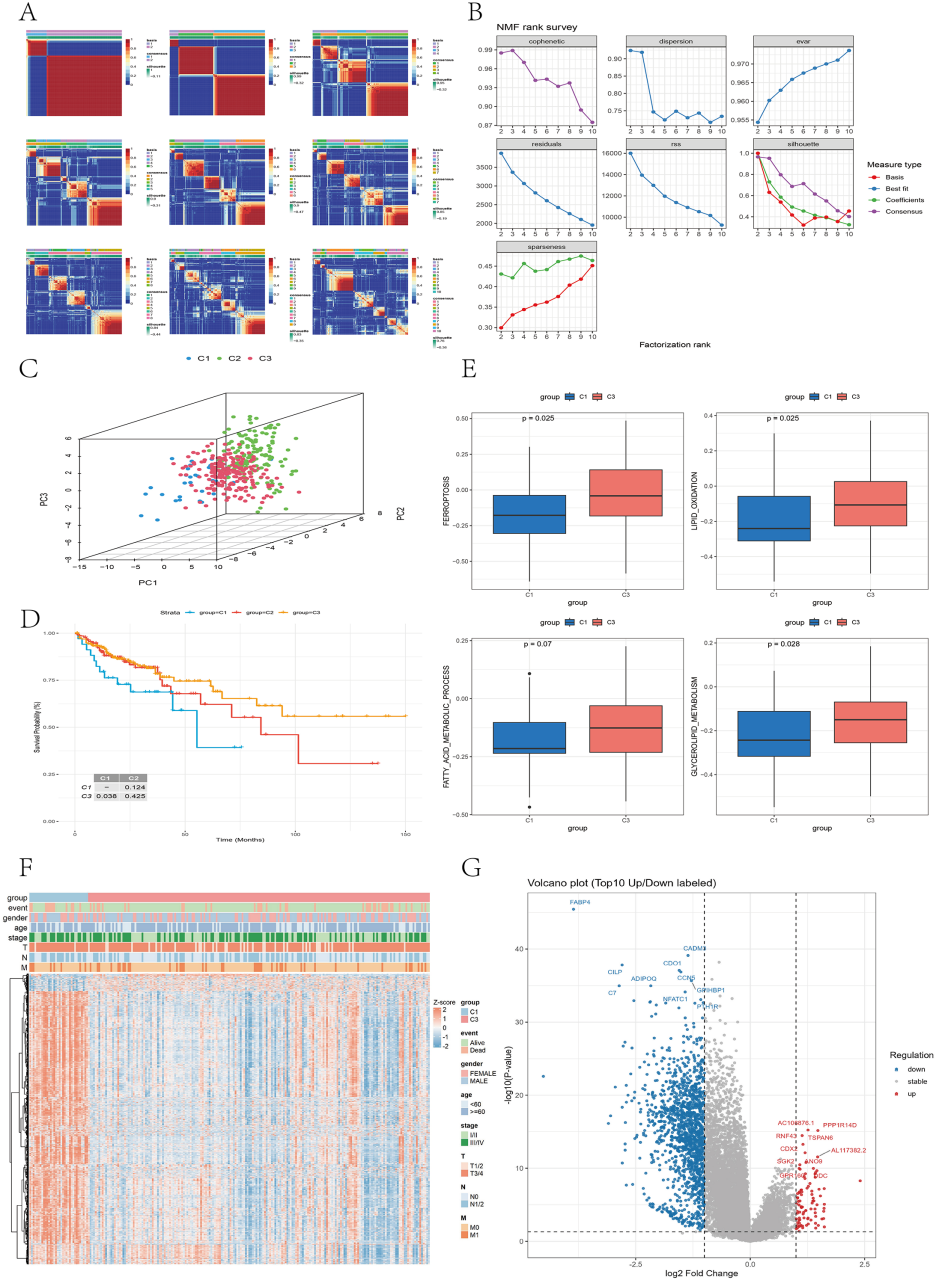
NMF cluster analysis. **(A)** NMF clustering heatmap showing the consensus matrix and silhouette coefficients for cluster numbers ranging from 2 to 10. **(B)** Line graph displaying metrics across factor ranks (2–10), including the cophenetic matrix, dispersion, and explained variance (evar), among others. **(C)** 3D PCA plot illustrating the distribution of the three subtypes after dimensionality reduction. **(D)** Survival curve showing significant differences in overall survival between Cluster-1 and Cluster-3 patients (P < 0.05). **(E)** Box plot comparing ferroptosis- and lipid metabolism-related pathway activities between Cluster-1 and Cluster-3. **(F)** Heatmap showing the expression profiles of differentially expressed genes (DEGs) between Cluster-1 and Cluster-3. Each column represents a patient sample, and each row represents a gene. Red indicates high expression, and blue indicates low expression. The upper annotation bars display clinical characteristics, including group, event, gender, age, stage, and T, N, and M classifications. Volcano plot illustrating the differentially expressed genes (DEGs) between the Cluster-1 and Cluster-3 subtypes. Red dots represent upregulated genes, blue dots represent downregulated genes. The top 10 significantly upregulated and downregulated genes are labeled in the plot.

On the basis of the 56 FRLMRGs, nonnegative matrix factorization (NMF) clustering was performed with k values ranging from 2 to 10. The consensus heatmaps for k = 2–10 (**Figure 3A**) illustrate the clustering stability across different ranks. At k = 2 or k = 3, the samples were clearly separated into distinct and homogeneous clusters, indicating strong within-cluster consistency and between-cluster independence. When k > 3, the consensus patterns became increasingly diffuse, suggesting a decline in clustering stability. As shown in **Figure 3B**, both the cophenetic coefficient and the mean silhouette width dropped sharply after k = 3, supporting this conclusion. To further evaluate clustering robustness, we manually computed the cumulative distribution function (CDF) and ΔCDF curves based on the consensus matrices across different k values. As shown in the **Supplementary Figure 1A**, the CDF curve increased rapidly from k = 2 to k = 3 and then plateaued, while the ΔCDF curve peaked at k = 3 and sharply declined thereafter. Taken together, these results indicate that k = 3 provides the most stable and parsimonious clustering solution, and was therefore selected as the optimal number of clusters. Cluster-1 included 34 CC samples, Cluster-2 included 139 samples, and Cluster-3 included 198 samples. 3D principal component analysis (PCA) confirmed clear spatial separation among the three clusters (**Figure 3C**). K–M survival analysis revealed that Cluster-1 patients had significantly worse overall survival (OS) than Cluster-3 patients did (P = 0.038) (**Figure 3D**), with no significant differences between Cluster-1 patients and Cluster-2 patients (P = 0.124) or between Cluster-2 patients and Cluster-3 patients (P = 0.425). Compared with Cluster 1, cluster 3 exhibited significantly higher activity scores for “ferroptosis” (adjusted P = 0.025, δ = −0.29), “lipid peroxidation” (adjusted P = 0.025, δ = −0.28), and “glycerolipid metabolism” (adjusted P = 0.028, δ = −0.26), along with an increasing trend for “fatty acid metabolism” (adjusted P = 0.07, δ = −0.22) (**Figure 3E**). Differential expression analysis is performed between Cluster-1 and Cluster-3 using the limma package in R (FDR = 0.05, |log_2_FC| > 1). A total of 90 upregulated genes and 1,427 downregulated genes were identified (**Supplementary Data 2**). The volcano plot (**Figure 3G**) and heatmap (**Figure 3F**) illustrate the overall distribution and expression patterns of these differentially expressed genes, highlighting distinct transcriptional landscapes between the two subtypes. As shown in the **Supplementary Figure 1B**, KEGG analysis primarily revealed key pathways such as chemokine signaling, phagosome system, and cell adhesion molecules. GO enrichment analysis further demonstrated biological processes including leukocyte-mediated adaptive immune response, extracellular matrix structural organization, and immunoglobulin-mediated immune response; cellular components such as the collagen-containing matrix, immunoglobulin complex, and basement membrane; and molecular functions involving antigen binding, glycosaminoglycan binding, and integrin binding. Collectively, these results suggest that inflammatory signaling, immune regulation, and extracellular matrix remodeling play critical roles in shaping the molecular distinctions between Cluster-1 and Cluster-3.

### 3.4 WGCNA and screening of ferroptosis- and lipid metabolism-related prognostic features

**Figure 4:**
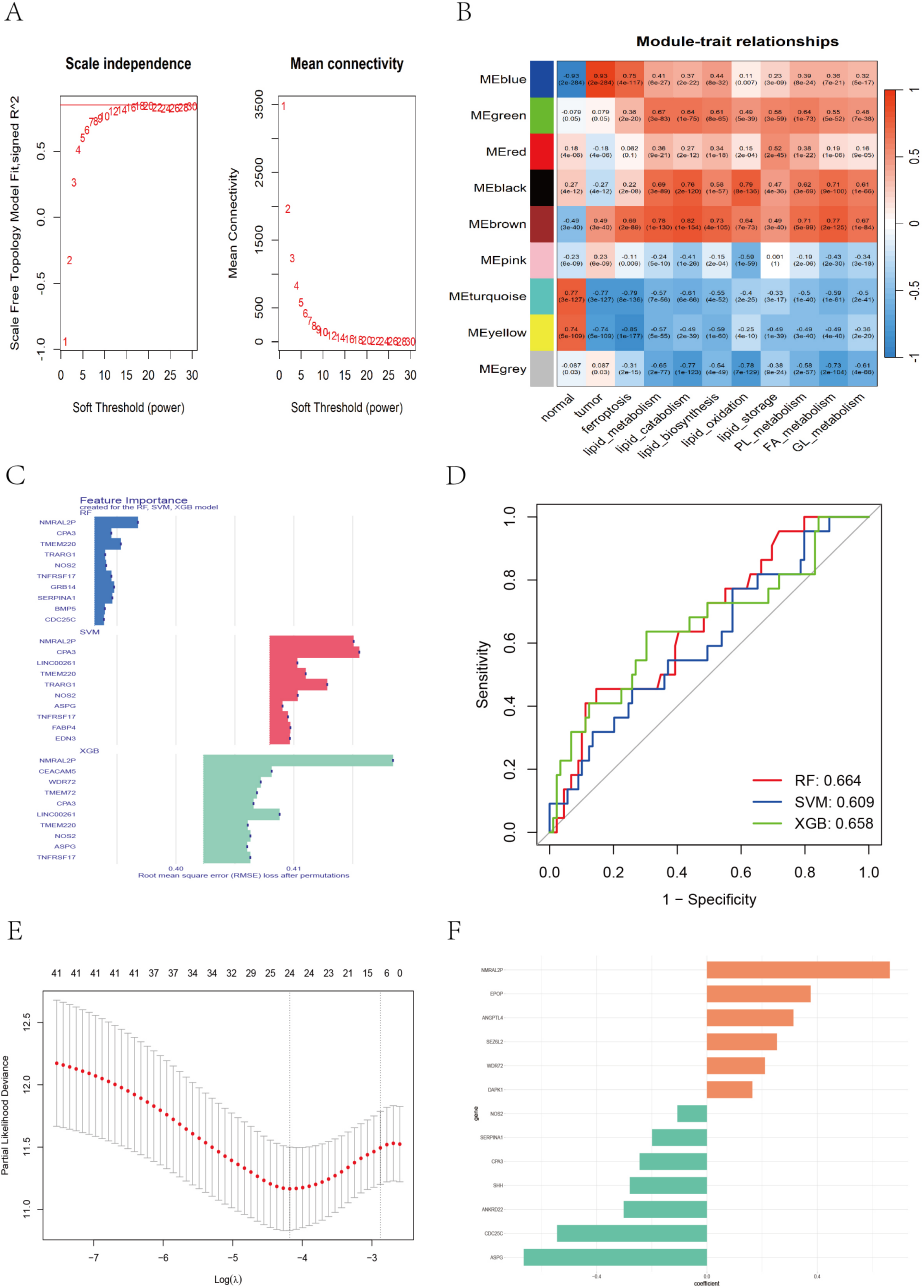
WGCNA and feature gene selection based on different machine learning models. **(A)** Soft threshold selection evaluation plot: the left plot showing the scale-free topology of the network, with the optimal soft threshold β = 16 determined on the basis of R^2 = 0.85. The right plot showing how the average connectivity between genes changes with the soft threshold. **(B)** The WGCNA heatmap displaying the correlation between different color modules and clinical traits. **(C)** Bar chart showing the top 10 genes ranked by importance across the RF, SVM, and XGB models. **(D)** ROC curve illustrating the classification performance of RF, SVM, and XGB. **(E)** LASSO cross-validation plot showing partial likelihood deviance across different log(λ) values. **(F)** Bar chart displaying feature coefficients from multivariate Cox regression.

To identify genes highly associated with tumorigenesis and ferroptosis-lipid metabolism, we performed weighted gene coexpression network analysis (WGCNA) to detect coexpression gene modules strongly correlated with these traits. During network construction, we determined that the optimal soft-thresholding power β was 16, as it achieved a scale-free topology fit index of 0.85 (**Figure 4A**), resulting in the identification of 9 modules. Among these, the blue module was strongly correlated with tumor status (r = 0.93, P < 0.001; **Figure 4B**). Additionally, ferroptosis and lipid metabolism traits were significantly correlated with the green (r_max = 0.67, P < 0.001), black (r_max = 0.79, P < 0.001), and brown (r_max = 0.82, P < 0.001) modules.

A total of 3,088 genes from the blue (1,476), green (202), black (151), and brown (1,259) modules were extracted. These genes were merged with DEGs between Cluster-1 and Cluster-3, and batch univariate Cox regression identified 60 genes (P < 0.05) (**Supplementary File 5**). Considering that the predictive performance of a single machine learning algorithm may be influenced by various factors, we applied three independent machine learning approaches—support vector machine (SVM), random forest (RF), and extreme gradient boosting (XGBoost)—to improve the accuracy and robustness of the prognostic model. Each method identified the top 30 genes most predictive of prognosis in CC patients (**Supplementary Files 6**-**8**). The top 10 genes contributing most to prognosis for each algorithm are shown in **Figure 4C**, and ROC curves for the three algorithms were plotted to evaluate performance (**Figure 4D**). We took the union of the top 30 genes identified by each algorithm, yielding a total of 42 candidate genes. We then applied LASSO regression to address multicollinearity and further reduce the number of genes in the risk model. The optimal lambda value was selected via 10-fold cross-validation. When lambda ≈ −4.18, a model with 24 genes was determined to be optimal (**Figure 4E**). Finally, we performed multivariate Cox regression with bidirectional stepwise selection, and when 13 genes were retained, the model achieved the lowest AIC value of 742.3 (**Supplementary File 9**), indicating an optimal balance between model complexity and goodness-of-fit. The regression coefficients of the 13 genes are shown in **Figure 4F**.

### 3.5 Establishment of the prognostic model and its association with other systems

In both the TCGA and GEO cohorts, the risk score for each patient was calculated via the following formula:

Risk score = (−0.244 × CPA3 mRNA) + (0.663 × NMRAL2P lncRNA) + (−0.543 × CDC25C mRNA) + (−0.280 × SHH mRNA) + (0.165 × DAPK1 mRNA) + (−0.302 × ANKRD22 mRNA) + (−0.199 × SERPINA1 mRNA) + (−0.664 × ASPG mRNA) + (0.210 × WDR72 mRNA) + (0.254 × SEZ6L2 mRNA) + (−0.107 × NOS2 mRNA) + (0.376 × EPOP mRNA) + (0.313 × ANGPTL4 mRNA).

**Figure 5:**
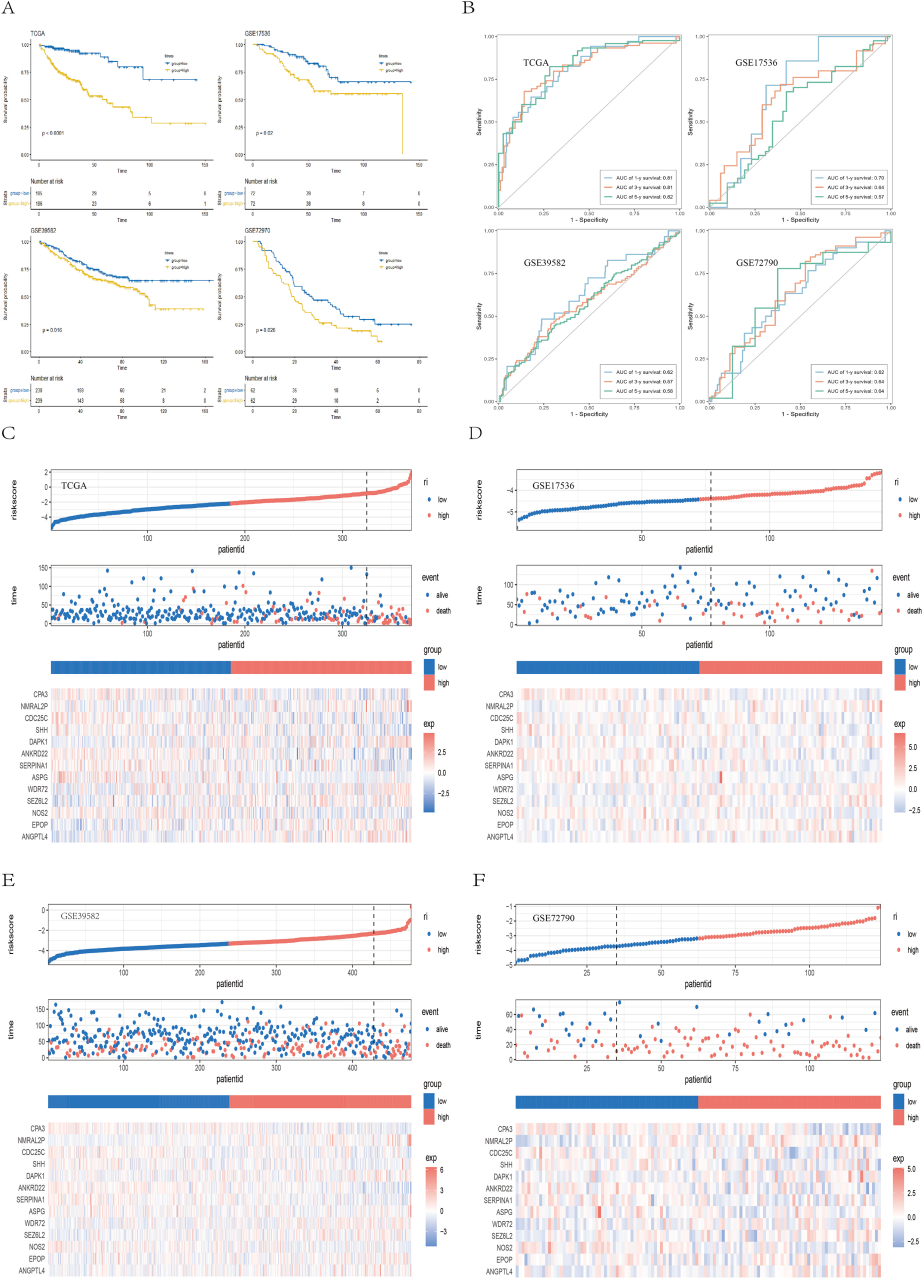
Validation of the prognostic model. **(A)** Kaplan–Meier survival curves showing survival differences between the high- and low-risk groups across the TCGA, GSE39582, GSE17536, and GSE72970 cohorts. **(B)** ROC curves demonstrating the predictive performance of the risk score for 1-, 3-, and 5-year survival in the TCGA and GEO cohorts. **(C–F)** Scatter plots of survival outcomes and heatmaps of gene expression data illustrating the distribution of different prognostic populations, as well as the expression patterns of the 13 prognostic genes.

Patients were classified into high-risk and low-risk groups according to the median risk score. Survival analyses performed in the TCGA training cohort and the GEO validation cohorts (GSE39582, GSE17536, and GSE72970) consistently revealed that patients in the high-risk group had significantly worse overall survival (OS) than those in the low-risk group did (TCGA: P < 0.001; GSE39582: P = 0.016; GSE17536: P = 0.020; GSE72970: P = 0.026) (**Figure 5A**). Receiver operating characteristic (ROC) curves indicated that the risk score exhibited strong predictive power for 1-, 3-, and 5-year survival in both the TCGA and GEO cohorts (**Figure 5B**). **Figures 5C–F** displays the detailed survival status of individual patients and heatmaps showing the expression profiles of the 13 core genes in the high- and low-risk groups. Additionally, high-risk patients also had worse disease-free survival (DFS) in the GSE17536 (P = 0.024) and GSE17538 (P = 0.011) datasets and worse disease-specific survival (DSS) in the GSE17536 (P = 0.016) and GSE87211 (P = 0.005) datasets (**Supplementary Figure 2**). We subsequently performed stratified analysis on the basis of clinical characteristics in the TCGA cohort to evaluate the applicability of the risk score across different subgroups. Patients were stratified by age (<60/≥60), sex (female/male), T stage (T1 + 2/T3 + 4), N stage (N0 + 1/N2), M stage (M0/M1), and TNM stage (I+II/III+IV). The results (**Supplementary Figures 3**, **4**) demonstrated that our risk model retained significant prognostic value across all subgroups (P < 0.05).

Among the three NMF-derived molecular subtypes, Cluster-1 (associated with the worst prognosis) had significantly higher risk scores than did Cluster-2 (P = 0.002) and Cluster-3 (P < 0.001) (**Supplementary Figure 5A**). In the consensus molecular subtype (CMS) classification system of colorectal cancer (CRC), CMS1 is referred to as the “immune subtype”, characterized by microsatellite instability (MSI) and infiltration of activated immune cells. CMS2, the “canonical subtype”, exhibits epithelial features with strong activation of the WNT and MYC pathways. CMS3, the “metabolic subtype”, is characterized by prominent metabolic dysregulation and reprogramming. CMS4 represents the “mesenchymal subtype”, characterized by epithelial-mesenchymal transition (EMT), stromal invasion, angiogenesis, and immunosuppressive properties and poor prognosis (29). Previous studies (29)have reported that patients with CMS1 tumors have poorer survival after recurrence, and those diagnosed at an early stage with CMS4 tumors have a significantly increased risk of distant relapse and death. We observed that CMS1 and CMS4 had significantly higher risk scores than did CMS2 and CMS3 (P < 0.001) (**Supplementary Figure 5B**). Survival analysis revealed a trend toward poorer overall survival (OS) in the CMS1 and CMS4 subtypes (**Supplementary Figure 5C**), although this difference was not statistically significant (P = 0.25). The Sankey diagram (**Supplementary Figure 5D**) revealed that the majority of Cluster-1 samples, which had the worst prognosis, were classified as CMS4, suggesting that Cluster-1 displays mesenchymal characteristics associated with tumor invasion, metastasis, and poor prognosis. Notably, most of the CMS1 and CMS4 samples were in the high-risk group, whereas the CMS2 and CMS3 samples were predominantly in the low-risk group. Therefore, both the poor-prognosis Cluster-1 and the CMS1/CMS4 subtypes were associated with higher risk scores, indicating that the risk score maintains robust prognostic value across different molecular classification systems.

### 3.6 Construction of the nomogram

**Figure 6:**
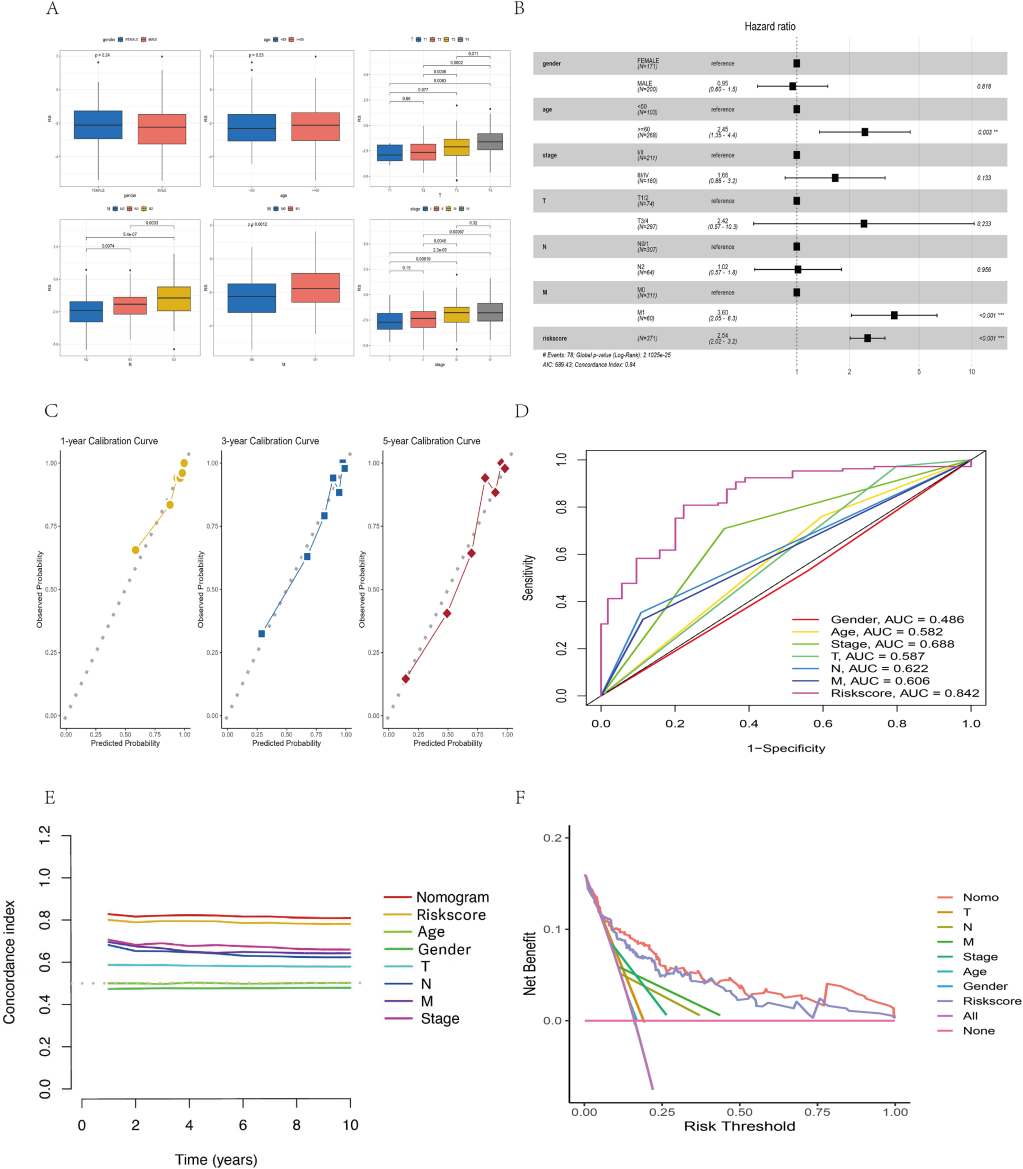
Construction and validation of the nomogram. **(A)** Box plot illustrating the relationships between the risk score (RS) and clinical features (sex, age, T stage, N stage, M stage, overall stage). **(B)** Forest plot showing multivariate Cox regression results, including hazard ratios (HRs), 95% confidence intervals (CIs), and p values for the risk score and clinical variables. **(C)** Calibration curves assessing the agreement between the predicted and observed survival outcomes. **(D)** ROC curves comparing the predictive performance of the nomogram and clinical parameters. **(E)** C-index line chart displaying predictive consistency across time points. **(F)** Decision curve analysis (DCA) evaluating the net clinical benefit of different predictors.

Next, we found that larger tumor size (P < 0.05), lymph node metastasis (P < 0.01), distant metastasis (P < 0.01), and advanced pathological stage (P < 0.01) were significantly associated with higher risk scores (**Figure 6A**). Multivariate Cox regression confirmed that the risk score based on 13 ferroptosis- and lipid metabolism-related genes was an independent prognostic factor (HR = 2.54, 95% CI: 2.02–3.20, P < 0.001) (**Figure 6B**). To improve predictive accuracy, we constructed a nomogram incorporating multiple factors, including age, sex, tumor stage, tumor size (T), lymph node involvement (N), distant metastasis (M), and the risk score (**Supplementary Figure 6**). The calibration curves for the 1-, 3-, and 5-year survival probabilities closely aligned with the actual outcomes (**Figure 6C**), indicating excellent predictive performance. To compare the sensitivity and specificity of the nomogram, risk score, and conventional clinicopathological features in predicting prognosis, we conducted time-dependent ROC analysis. The nomogram yielded the highest area under the curve (AUC = 0.882) (**Figure 6D**), outperforming both the risk score alone (AUC = 0.842) and all other individual clinical variables. Furthermore, the C-index (**Figure 6E**) and decision curve analysis (DCA) (**Figure 6F**) consistently demonstrated that our nomogram provided the greatest net benefit and surpassed traditional models, thereby offering more substantial value for clinical decision-making. These findings suggest that the nomogram is a superior prognostic tool and may serve as a sensitive indicator for predicting outcomes in CC patients.

### 3.7 Gene mutations and the tumor immune microenvironment

To investigate whether differences in gene mutations exist between the high- and low-risk groups, we downloaded and analyzed simple nucleotide variation data from the TCGA database. A summary of the mutation information is shown in the bar plot (**Supplementary Figure 7A**). Among the mutation types, missense mutations were the most common. Single nucleotide polymorphisms (SNPs) account for the majority of the mutations, with C>T transitions being the most frequent. Analysis of the tumor mutation burden (TMB) revealed that the high-risk group had significantly higher TMB values (P = 0.002) (**Supplementary Figure 7B**). The top 20 mutated genes were visualized for both groups (**Supplementary Figures 7C, D**), which revealed overall higher mutation frequencies in the high-risk group. The five most frequently mutated genes were APC, TP53, TTN, KRAS, and PIK3CA in both groups, with slightly different mutation rates. **Supplementary Figure 7E** display the distribution of copy number variations across different chromosomal regions. Notably, SHH, located on chromosome 7, presented the highest CNV gain rate (47.23%) and the lowest loss rate (2.57%). Similarly, SEZ6L2, NOS2, EPOP, CPA3, and ANGPTL4 had greater CNV gains than losses did, whereas WDR72, SERPINA1, ASPG, ANKRD22, and CDC25C presented more CNV losses than gains did. DAPK1, located on chromosome 9, presented nearly equal proportions of CNV gains and losses (**Supplementary Figure 7F**).

Among the 13 ferroptosis- and lipid metabolism–related signature genes, CPA3, DAPK1, and ANGPTL4 exhibited significant positive correlations with most immune checkpoint genes, suggesting that higher expression of these genes may be associated with an immune-activated tumor microenvironment. In contrast, NMRAL2P and SHH showed significant negative correlations with multiple immune checkpoint genes, indicating that tumors with higher expression of these genes tend to display a “cold tumor” immune microenvironment, which may correspond to a poorer response to immunotherapy(**Supplementary Figure 8**). However, it is important to note that this heatmap reflects only statistical co-expression relationships at the transcriptional level and does not imply direct regulatory interactions or causal signaling relationships among these genes.

**Figure 7:**
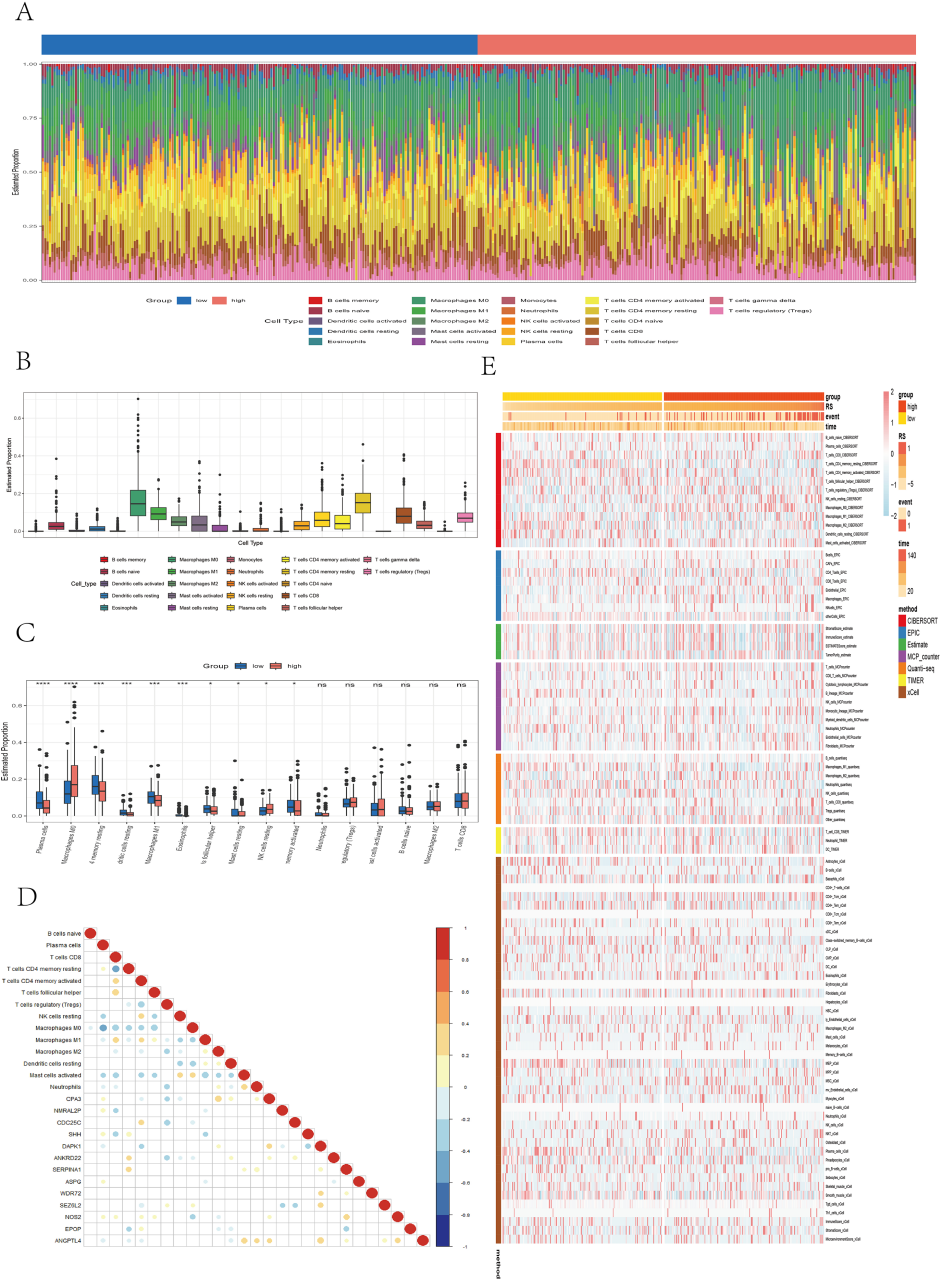
Analysis of immune microenvironment differences. **(A)** Stacked bar plot illustrating the immune cell infiltration landscape. **(B)** Box plot showing the estimated proportions of different immune cell types. **(C)** Box plot comparing immune cell infiltration between risk groups. **(D)** Correlation dot plot presenting the relationships between immune cells and core genes, with color depth and circle size indicating correlation strength and significance. **(E)** Heatmap displaying immune infiltration differences across multiple algorithms, including CIBERSORT, EPIC, ESTIMATE, MCPcounter, QUANTISEQ, TIMER, and xCell.

In the TCGA cohort, the infiltration levels of 22 immune cell types were compared between the high- and low-risk groups (**Figure 7A**). Among them, M0 macrophages and resting memory CD4+ T cells presented the highest levels of infiltration (**Figure 7B**). The low-risk group was associated with significantly higher levels of plasma cells (P < 0.0001), resting memory CD4+ T cells (P < 0.001), resting dendritic cells (P < 0.001), M1 macrophages (P < 0.001), eosinophils (P < 0.001), follicular helper T cells (P < 0.05), resting mast cells (P < 0.05), and activated memory CD4+ T cells (P < 0.05). In contrast, the high-risk group presented increased infiltration of M0 macrophages (P < 0.0001) and resting NK cells (P < 0.05) (**Figure 7C**). A correlation dot plot displaying the correlation between immune cell infiltration levels and the expression of the 13 core genes was also generated (**Figure 7D**). As shown, M0 macrophages were significantly negatively correlated (P < 0.01) with multiple immune cells, including B cells, plasma cells, CD4+ T cells, CD8+ T cells, and follicular helper T cells, suggesting that M0 macrophages in this context may exhibit immunosuppressive properties. In contrast, M1 macrophages were not only significantly negatively correlated with M0 macrophages (P < 0.01) but also positively correlated (P < 0.01) with CD8+ T cells, activated memory CD4+ T cells, and follicular helper T cells, indicating an immunoactivating role. **Figure 7E** presents immune infiltration heatmaps derived from various algorithms including CIBERSORT, EPIC, ESTIMATE, MCPcounter, QUANTISEQ, TIMER, and xCell. Notably, the low-risk group was associated with increased infiltration of B cells and CD4+ T cells, as determined by the EPIC, MCPcounter, and xCell algorithms (P < 0.05), whereas the high-risk group presented increased infiltration of fibroblasts (P < 0.05). In conclusion, significant immune profile differences between low- and high-risk groups highlight the need to further explore the immunological mechanisms of colorectal cancer and their implications for immunotherapy.

### 3.8 Impact of the prognostic model on therapeutic decision-making

**Figure 8:**
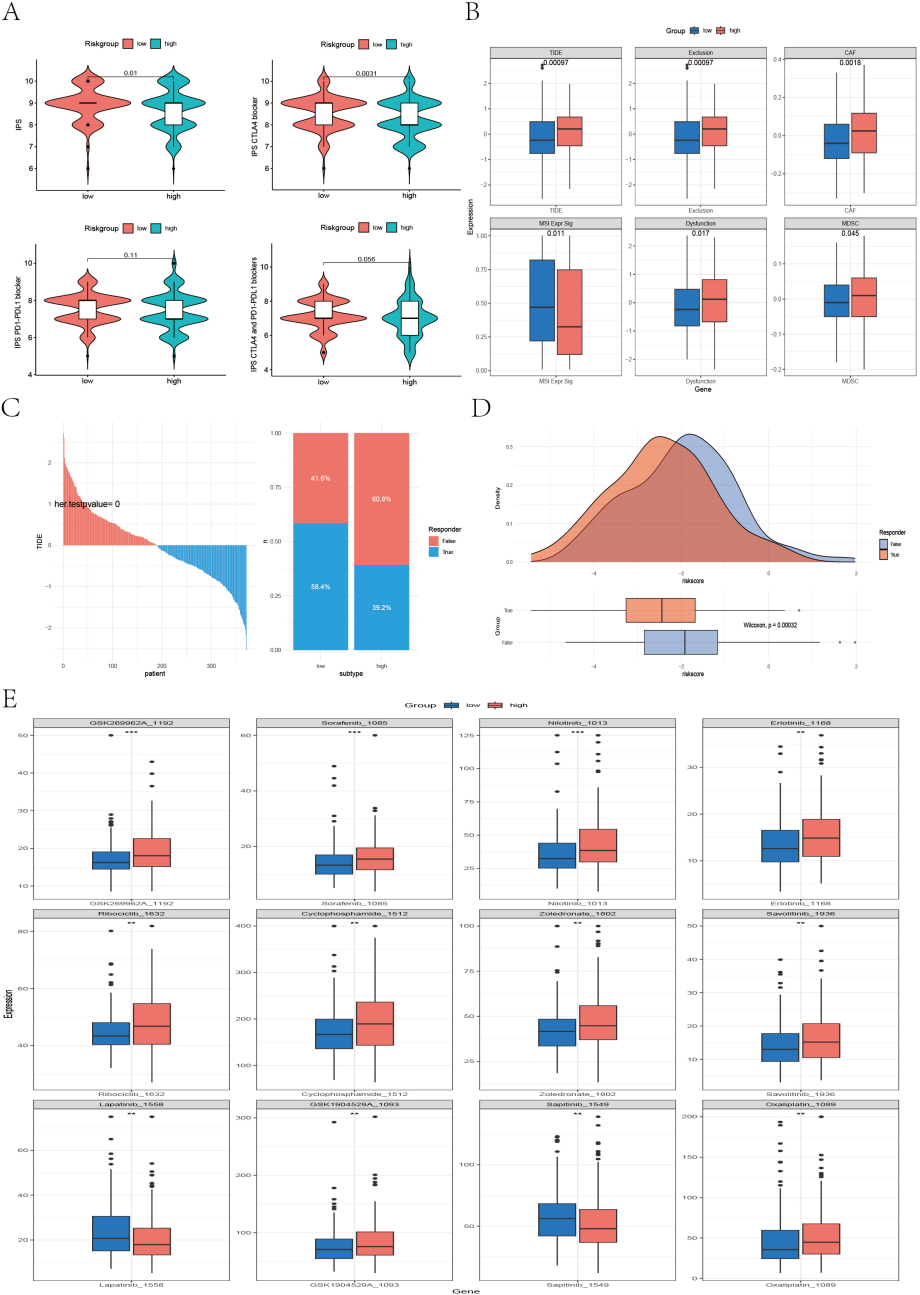
Differences between the high- and low-risk groups in IPS, TIDE, and drug sensitivity analyses. **(A)** Violin plots illustrating differences in the immunophenoscore (IPS) between different risk groups. **(B)** Boxplots illustrating differences in TIDE-related scores—including TIDE, CAF, MDSC, and MSI—between the high- and low-risk groups. **(C, D)** Bar plots, density plots, and boxplots depicting the differences in immunotherapy response between the high- and low-risk groups on the basis of the TIDE dataset. **(E)** Boxplots showing differences in the IC50 values of common chemotherapeutic and targeted drugs between the two risk groups.

We further evaluated the ability of the risk score to predict the clinical response to immunotherapy in colon cancer (CC) patients. First, we compared the immunophenoscore (IPS) between the high- and low-risk groups. The results revealed that the low-risk group had significantly higher IPS scores (P = 0.01). Notably, the IPS scores for anti-CTLA-4 therapy were also significantly elevated in the low-risk group (P = 0.0031), whereas the IPS scores for anti-PD-1/PD-L1 therapy (P = 0.11) and combined anti-CTLA4/PD-1/PD-L1 therapy (P = 0.056) also tended to be greater in the low-risk group, although the difference was not statistically significant. Overall, these findings suggest that tumors in the low-risk group exhibit greater immunogenicity and may be more responsive to immune checkpoint inhibitors (ICIs), such as CTLA-4 and PD-1/PD-L1 inhibitors (**Figure 8A**). We further analyzed the relationships between risk groups and tumor-infiltrating cytotoxic T lymphocyte (CTL) exclusion and dysfunction via the Tumor Immune Dysfunction and Exclusion (TIDE) scoring algorithm. We observed that the high-risk subgroup had significantly elevated TIDE scores (P < 0.001), T-cell exclusion (P < 0.001), and T-cell dysfunction (P = 0.017), as well as scores for cancer-associated fibroblasts (CAFs) (P = 0.002) and myeloid-derived suppressor cells (MDSCs) (P = 0.045), indicating that immune evasion is more prevalent in the high-risk subgroup (**Figure 8B**). In contrast, the low-risk subgroup had a significantly greater microsatellite instability (MSI) score (P = 0.011). Additionally, data from the TIDE platform indicated that the immune therapy response rate was significantly greater in the low-risk group and that the risk scores of responders were significantly lower than those of nonresponders (P < 0.001) (**Figures 8C, D**), suggesting that low-risk patients are more likely to benefit from anti-PD-1 and anti-CTLA-4 immunotherapy. In summary, the high-risk group shows a correlation with increased stromal infiltration and enhanced immunosuppressive features in the tumor microenvironment, which may be associated with immune escape and reduced efficacy of ICI therapy. To validate the irreplaceable role of the risk score in guiding chemotherapy and targeted therapy decisions, we evaluated predicted drug sensitivities across risk groups. The following drugs were predicted to be more effective in the low-risk group: sorafenib (P < 0.001), nilotinib (P < 0.001), erlotinib (P < 0.01), ribociclib (P < 0.01), cyclophosphamide (P < 0.01), zoledronic acid (P < 0.01), savolitinib (P < 0.01), and oxaliplatin (P < 0.01). In contrast, lapatinib (P < 0.01) and sapitinib (P < 0.01) may provide greater benefits to high-risk patients (**Figure 8E**). These findings highlight the potential of the risk score to inform precision chemotherapy strategies and promote personalized treatment approaches for CC patients.

**Figure 9:**
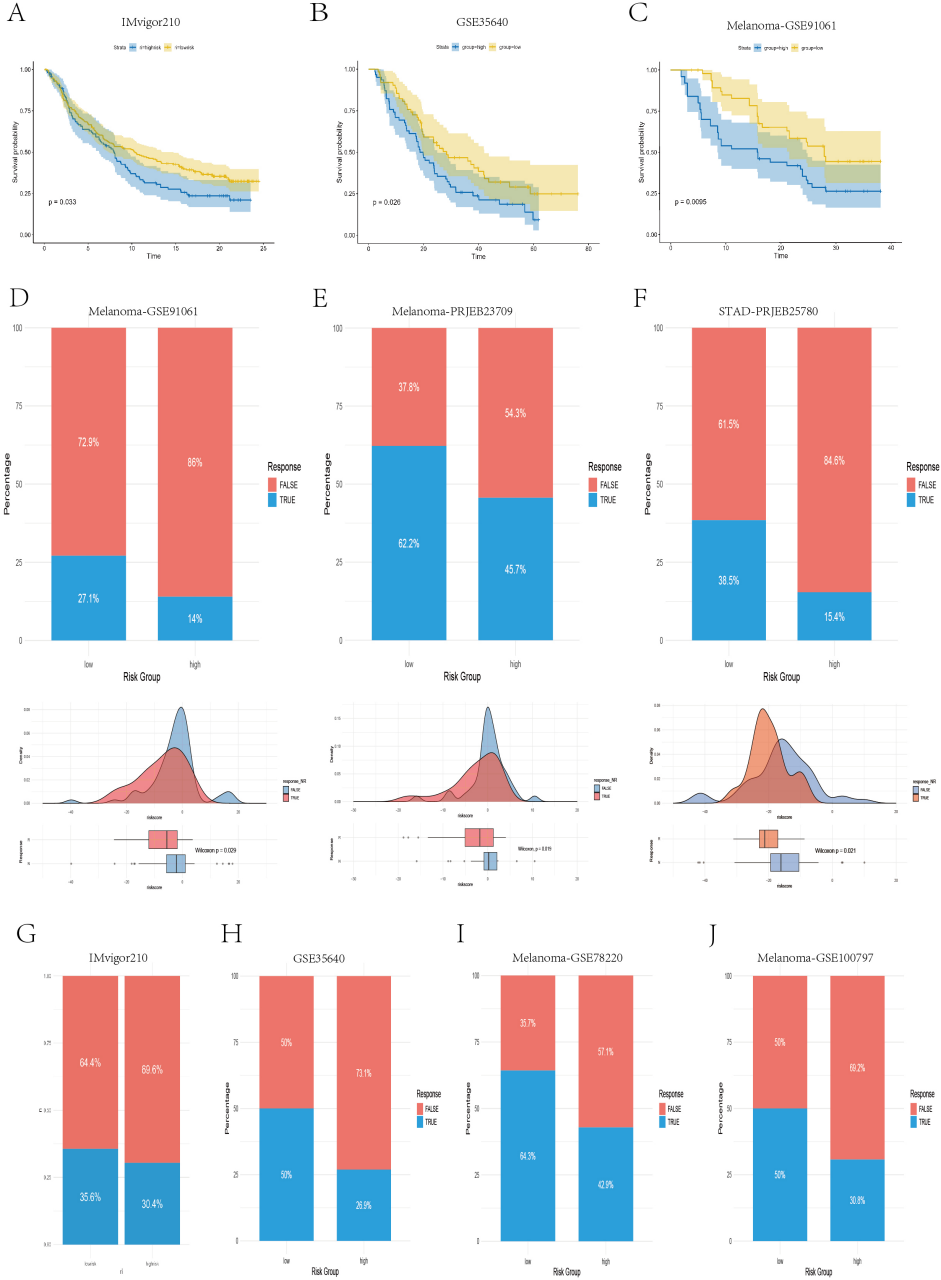
Real-world validation of immunotherapy efficacy across independent cohorts. **(A–C)** Kaplan–Meier survival curves illustrating the survival differences between the high- and low-risk groups in the IMvigor210, GSE35640, and GSE91061 cohorts. **(D–F)** Bar plots, density plots, and box plots displaying the differences in immunotherapy responses between risk groups across three datasets: Melanoma-GSE91061, Melanoma-PRJEB23709, and STAD-PRJEB25780. **(G–J)** Bar plots demonstrating the disparities in immunotherapy response between the high- and low-risk groups in four datasets: IMvigor210, GSE35640, Melanoma-GSE78220, and Melanoma-GSE100797.

Moreover, to further evaluate the ability of our model to predict immunotherapy efficacy, we applied the model to multiple real-world immunotherapy cohorts, ultimately confirming the reliability of the IPS- and TIDE-based conclusions. As shown in **Figures 9A–C**, in the IMvigor210 cohort (anti–PD-L1), GSE35640 cohort (MAGE-A3), and Melanoma-GSE91061 cohort (anti–PD-1), patients in the high-risk group exhibited significantly shorter overall survival than those in the low-risk group did (P < 0.05), which was consistent with the results previously observed in the TCGA and multiple GEO validation datasets. In the Melanoma-GSE91061 (anti–PD-1), Melanoma-PRJNA23709 (anti–PD-1 + anti–CTLA4), and STAD-PRJEB25780 (anti–PD-1) cohorts, the low-risk group had a higher immunotherapy response rate, and the risk scores of responders were significantly lower than those of nonresponders (P < 0.05) (**Figures 9D–F**), suggesting that these patients may benefit more from anti–PD-1 and anti–CTLA4 therapies. Similarly, in the IMvigor210 (anti–PD-L1), GSE35640 (MAGE-A3), Melanoma-GSE78220 (anti–PD-1), and Melanoma-GSE100797 (ACT) cohorts, the low-risk group also presented higher response rates to immunotherapy (**Figures 9G–J**); however, these differences did not reach statistical significance (P > 0.05).

### 3.9 Enrichment analysis results of the prognostic model

**Figure 10:**
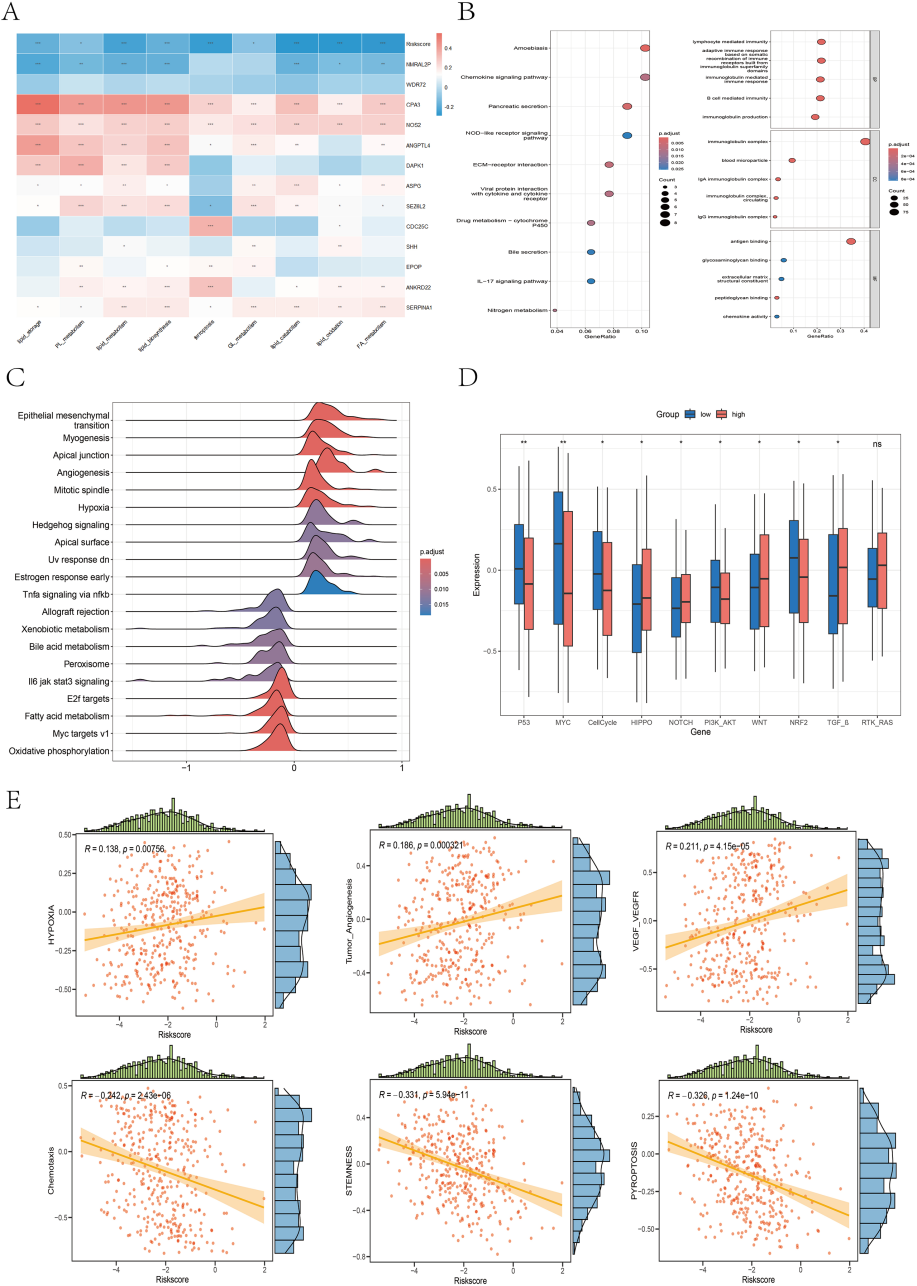
Differences in the enrichment or activity of various biological functions or pathways between the high- and low-risk groups. **(A)** Correlation heatmap illustrating the relationships between the 9 ferroptosis- and lipid metabolism-related pathways, the risk score, and the 13 core genes. **(B)** Bubble plots displaying the KEGG (left) and GO (right) enrichment analysis results of DEGs between the high- and low-risk groups. **(C)** Ridge plot presenting the GSEA enrichment results based on the hallmark gene sets; darker red denotes more significant adjusted P values. **(D)** Boxplot illustrating the differences in the activity levels of 10 classic carcinogenic signaling pathways between the high- and low-risk groups. **(E)** Scatter plots with marginal distributions depicting the correlation between the risk score and six representative biological pathways.

We constructed a heatmap illustrating the correlations between 9 ferroptosis–lipid metabolism-related pathways and the 13 core genes, as well as the risk score (**Figure 10A**). We found that two protective genes—CPA3 and NOS2—were significantly positively correlated with ferroptosis and multiple lipid metabolism pathways (P < 0.001). Among the two risk genes—NMRAL2P and WDR72—we observed varying degrees of negative correlations or negative trends with ferroptosis and lipid metabolism. Importantly, the risk score itself was significantly negatively correlated with ferroptosis and all lipid metabolism pathways (P < 0.05). Differential expression analysis of the high- versus low-risk groups was performed using the limma package with thresholds of FDR = 0.05 and |log2FC| > 0.585, resulting in 50 upregulated and 266 downregulated genes (**Supplementary Data 3**). Subsequent GO and KEGG enrichment analyses revealed that these DEGs were enriched primarily in immune regulation, inflammation, chemokine activity, and ECM composition and interactions, highlighting their potential roles in tumor signaling and progression (**Figure 10B**). GSVA of the hallmark gene set revealed that the high-risk group was enriched in epithelial–mesenchymal transition, apical junction, angiogenesis, hypoxia, and hedgehog signaling, whereas the low-risk group was enriched in bile acid metabolism, peroxisomes, fatty acid metabolism, and oxidative phosphorylation (**Figure 10C**). Previous studies (35–38) have reported strong links between peroxisomes, fatty acid metabolism, oxidative phosphorylation, and ferroptosis, supporting our hypothesis that the interplay among ferroptosis, lipid metabolism, and oxidative pathways in the low-risk group may favor an anti-tumor metabolic state, which is associated with improved prognosis. To assess the differential activation of cancer-related pathways between risk groups, we compared the activity of 10 classical oncogenic signaling pathways via boxplots (**Figure 10D**). We found significant differences in 9 of the 10 pathways, including the CellCycle, Hippo, MYC, Notch, Nrf2, PI3K–AKT, TGF-β, TP53, and Wnt pathways, which aligns with previous research (26). We also analyzed the correlations between the risk score and the six selected pathway activity scores. As shown in **Figure 10E**, the risk score was positively correlated with hypoxia, tumor angiogenesis, and VEGF–VEGFR signaling. In contrast, chemotaxis, pyroptosis, and stemness scores were negatively correlated with risk scores.

The GSEA results revealed that the high-risk group was significantly enriched in pathways associated with aggressive cancer features, including epithelial–mesenchymal transition (EMT), pseudopod chemotaxis, cell migration, and upregulation of metastatic activity (**Supplementary Figure 9A**), indicating a high propensity for invasion and metastasis in high-risk patients. Additionally, the high-risk group was enriched in extracellular matrix (ECM) biosynthesis processes (**Supplementary Figure 9B**), such as the synthesis of collagens, proteoglycans, glycoproteins, glycosaminoglycans, and chondroitin sulfate. These patients also showed enrichment in ECM-receptor interactions, integrin–cell surface interactions, focal adhesion, adhesion junctions, laminin interactions, and L1CAM interactions (**Supplementary Figure 9C**), further supporting their invasive potential and active remodeling of the tumor microenvironment. In contrast, the low-risk group was significantly enriched in biological processes related to fatty acid metabolism, cholesterol and oxysterol biosynthesis, mitochondrial fatty acid β-oxidation, mitochondrial function, respiratory electron transport, oxidative phosphorylation, and biological oxidation reactions (**Supplementary Figure 9D**).

### 3.10 Single-cell analysis

#### 3.10.1 Identification of single-cell subpopulations

**Figure 11:**
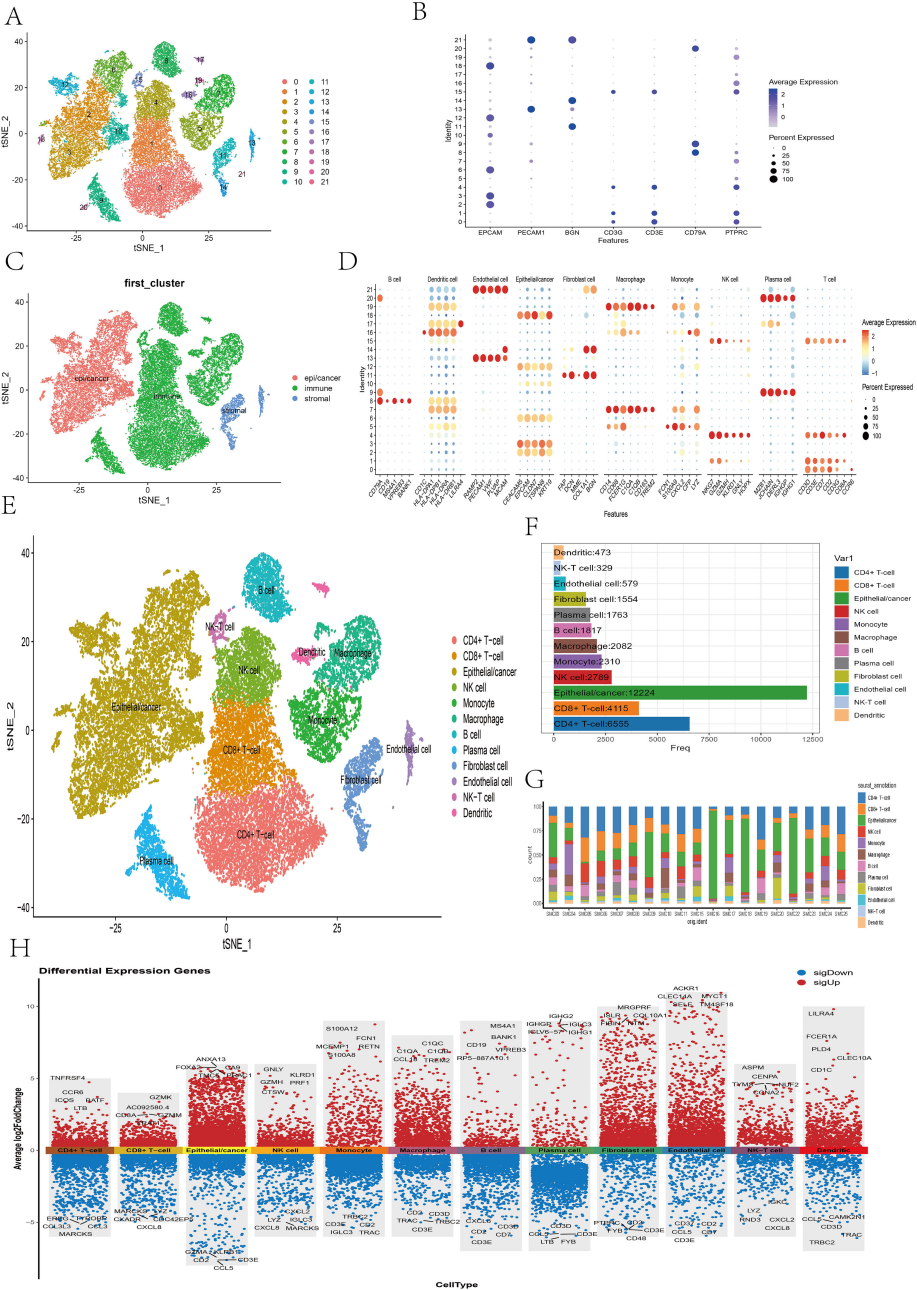
Identification of cell subpopulations on the basis of marker genes. **(A)** The t-SNE plot showing the clustering results of single-cell data after dimensionality reduction. **(B)** Bubble plot showing the expression levels of marker genes for three major cell types (epithelial/cancer cells, immune cells, and stromal cells) across cell clusters. **(C)** The t-SNE plot illustrating the first round of manual classification results. **(D)** Bubble plot presenting the expression patterns of marker genes for 10 cell types (B cells, plasma cells, T cells, NK cells, monocytes, dendritic cells, macrophages, fibroblasts, endothelial cells, and epithelial/cancer cells) across cell clusters. **(E)** The t-SNE plot showing the second round of manual classification results. **(F, G)** Bar plots displaying the proportion of each cell type and their distribution across individual samples. **(H)** Heatmap showing the differential gene expression analysis results across different cell types, with red indicating upregulated genes and blue indicating downregulated genes. The five genes whose expression was most significantly upregulated or downregulated in each cell type are highlighted.

To comprehensively investigate the heterogeneity of the tumor microenvironment (TME) in colon cancer, we analyzed the single-cell RNA-seq dataset GSE132465. After quality control and normalization, a total of 36,590 cells from 19 tumor samples were retained for further analysis. These cells were clustered into 22 subpopulations (**Figure 11A**). On the basis of reference marker genes (epithelial/cancer cell: EPCAM; immune cell: PTPRC, CD3G, CD3E, CD79A; stromal cell: BGN, PECAM1), the 22 clusters were broadly categorized into three major cell types: epithelial/cancer cells (clusters 2, 3, 6, 10, 12, 18), immune cells (clusters 0, 1, 4, 5, 7, 8, 9, 15, 16, 17, 19, 20), and stromal cells (clusters 11, 13, 14, 21) (**Figure 11B**). t-SNE visualization revealed the distributions of these three cell types (**Figure 11C**). On the basis of the expression of canonical marker genes, the 22 clusters were further annotated into 12 cell types: B cells, plasma cells, CD4+ T cells, CD8+ T cells, NK cells, NK-T cells, monocytes, dendritic cells, macrophages, fibroblasts, endothelial cells, and epithelial/cancer cells (**Figures 11D, E**). Notably, T cells were divided into CD4+ T cells and CD8+ T cells on the basis of CD8A expression, and cluster 15 coexpressed NK- and T- cell markers and was thus labeled NK-T cells. **Figure 11F** shows the proportion of each cell type, with epithelial/cancer cells being the most abundant, followed by CD4+ T cells. **Figure 11G** displays the distribution of cell types across individual samples. **Figure 11H** presents the top five upregulated and downregulated genes for each cell type.

#### 3.10.2 Identification of tumor cells and pseudotime analysis

**Figure 12:**
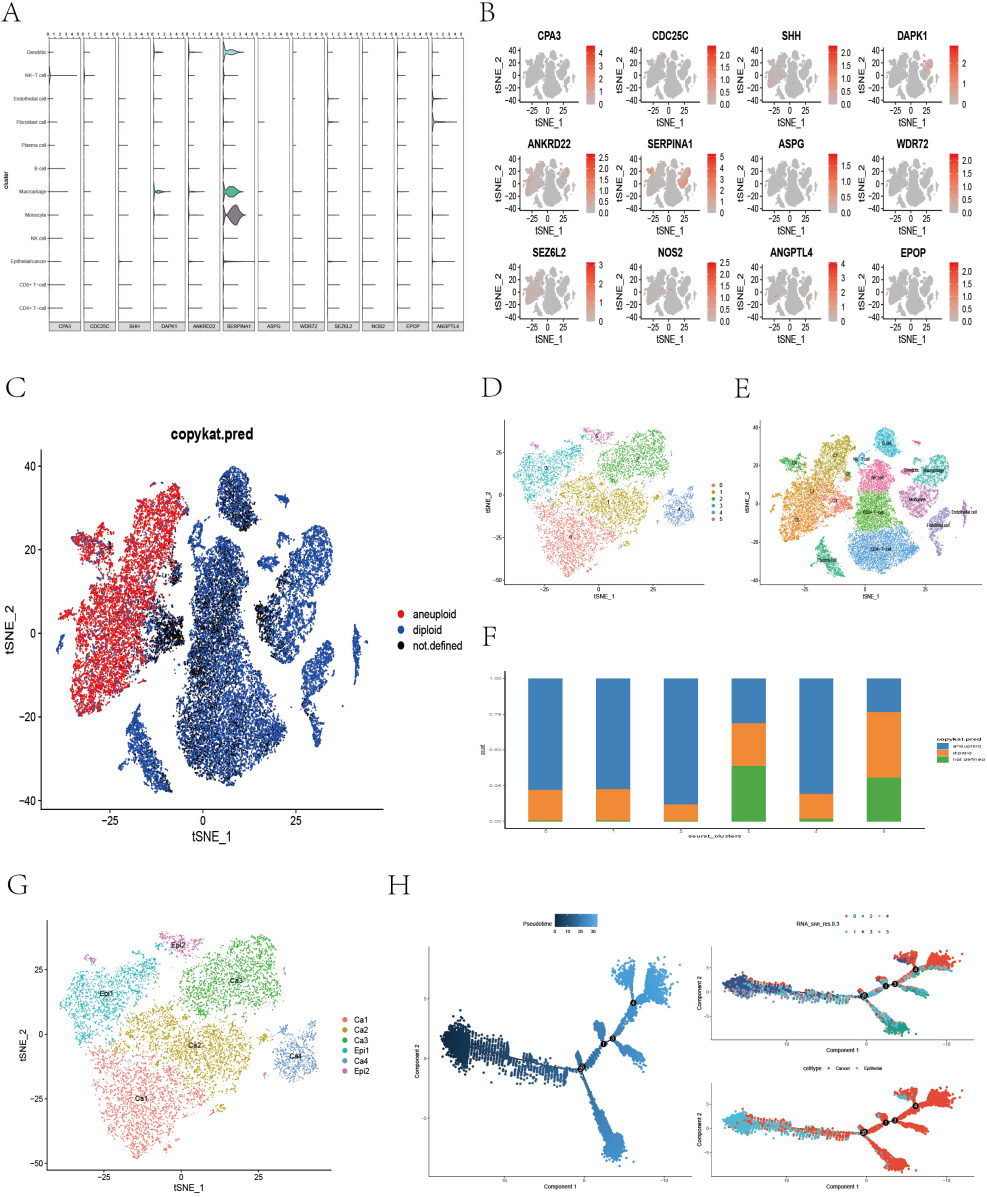
Distribution of model genes across different cell types, copycat analysis and pseudotime analysis. **(A, B)** Violin plots and t-SNE plots illustrating the expression patterns of 12 core genes across different cell types. **(C)** The t-SNE plot showing the distribution of diploid and aneuploid cells. **(D)** The t-SNE plot presenting the reclustering results of epithelial/cancer cell subpopulations. **(E)** This plot showing the mapping of reclustered epithelial/cancer subpopulations onto the second round of manually annotated t-SNE clusters. **(F)** A bar plot displaying the proportion of diploid and aneuploid cells within each reclustered epithelial/cancer cell subtype. **(G)** The t-SNE plot showing the reannotation of epithelial/cancer cell subpopulations. **(H)** The trajectory inference plot illustrating the pseudotime progression of epithelial and tumor clusters, with colors transitioning from dark blue to light blue indicating development from an initial to a mature cellular state.

We visualized the distribution of 12 core genes (excluding NMRAL2P) across different cell types (**Figures 12A, B**), revealing that CDC25C, SHH, ASPG, WDR72, and NOS2 were expressed mainly in epithelial/cancer cells; DAPK1, ANKRD22, and SERPINA1 were expressed primarily in epithelial/cancer cells, monocytes, and macrophages; and SEZ6L2 and ANGPTL4 were expressed predominantly in epithelial/cancer cells and fibroblasts. We then used the copykat R package to identify diploid (benign) and aneuploid (malignant) epithelial cells (**Figure 12C**). The epithelial/cancer cells were clustered into six subtypes (**Figures 1**, **2D**) and mapped to the t-SNE plot of the 12 cell types (**Figure 12E**). Clusters 0, 1, 2, and 4 had a greater proportion of aneuploid cells and were considered malignant subgroups, whereas clusters 3 and 5 had lower aneuploid proportions and were more likely epithelial subgroups (**Figure 12F**), as visualized via the t-SNE plot (**Figure 12G**). Pseudotime analysis of epithelial/cancer subpopulations in colon cancer was performed viaMonocle2. Clusters 3 and 5 were located mainly at the beginning of the pseudotime trajectory and gradually decreased, whereas clusters 0, 1, 2, and 4 increased as the pseudotime progressed (**Figure 12H**), suggesting a transition from an epithelial state to a malignant state. We further extracted all aneuploid cells within the epithelial/cancer subgroup and reclustered them into six tumor cell subtypes (**Supplementary Figure 10A**). Clusters 0, 1, and 2 had higher FRLM activity scores, whereas clusters 3, 4, and 5 had lower scores (**Supplementary Figure 10B**), which was visualized via t-SNE (**Supplementary Figure 10C**). To investigate functional and pathway differences between high- and low-FRLM-scoring tumor cells, GSVA enrichment analysis was conducted based on the hallmark gene sets. The low-score group was enriched in epithelial–mesenchymal transition (EMT), angiogenesis, apical junction, apical surface, Notch signaling, and the inflammatory response, whereas the high-score group was enriched in apoptosis, adipogenesis, fatty acid metabolism, peroxisome, oxidative phosphorylation, the reactive oxygen species pathway, MYC targets, p53 signaling, and Wnt/β-catenin signaling (**Supplementary Figure 10D**), which is consistent with the signaling pathways identified in the bulk transcriptome analysis.

#### 3.10.3 Reclustering of macrophages

**Figure 13:**
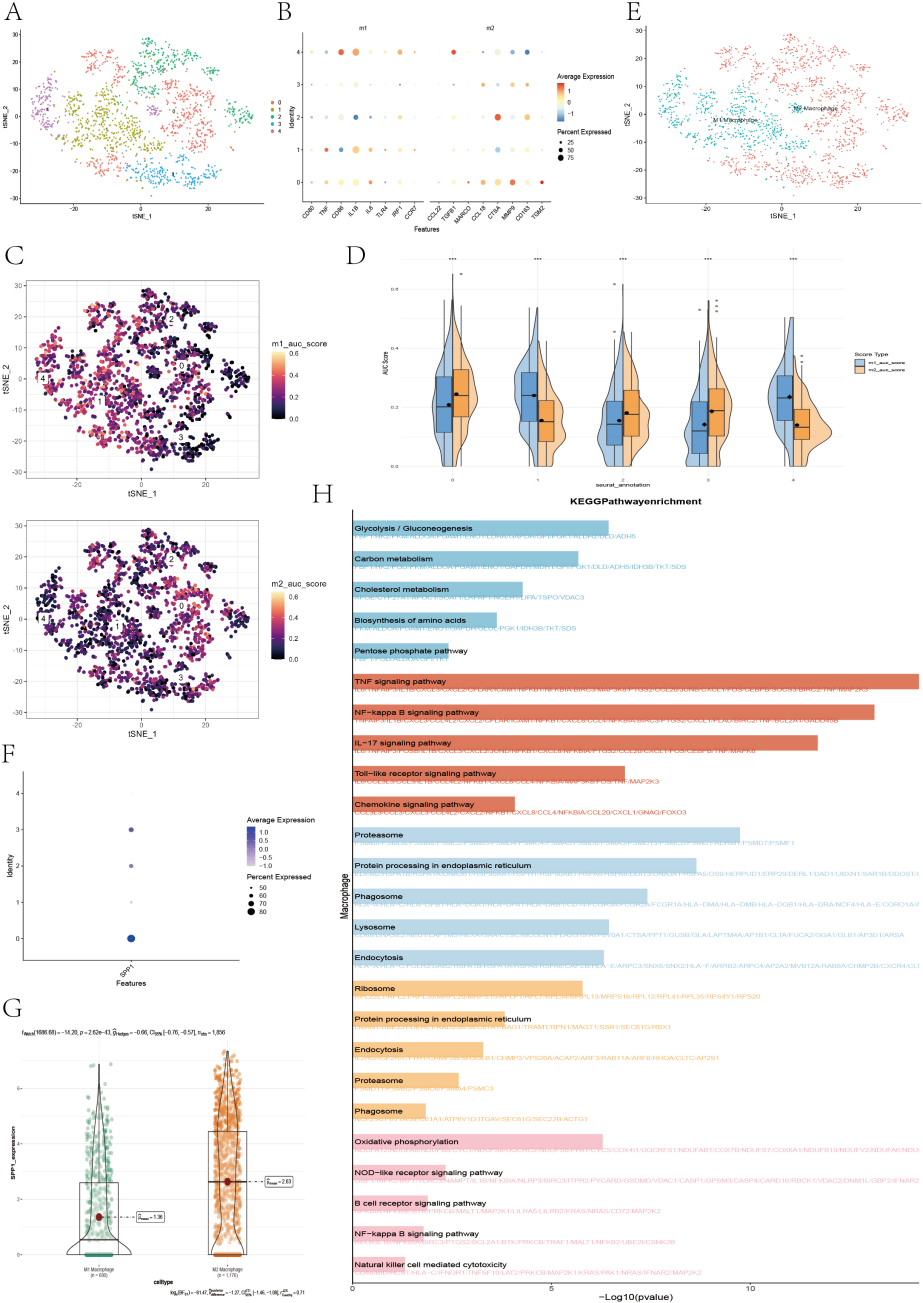
Reannotation of macrophage subpopulations on the basis of M1/M2 macrophage signature genes and KEGG pathway enrichment analysis. **(A)** The t-SNE plot displaying the results of the clustering of the macrophage populations. **(B)** Bubble plot illustrating the expression patterns of M1 and M2 macrophage marker genes across different subpopulations. **(C)** The t-SNE plot showing the distribution of AUCell scores calculated from M1/M2 marker gene sets across macrophage subclusters. **(D)** The half-violin and box plots presenting the differences in the AUCell scores of the M1 and M2 macrophage signature genes among the subclusters. **(E)** The t-SNE plot visualizing the reannotation of macrophage subclusters on the basis of M1/M2 signature scores. **(F)** Bubble plot showing the expression of the SPP1 gene in various macrophage subpopulations. **(G)** Violin plot depicting the differential expression of the SPP1 gene between the M1 and M2 macrophage subgroups. **(H)** Bar plot displaying the results of KEGG pathway enrichment analysis for different macrophage subtypes.

To explore macrophage subtype characteristics in the TME, macrophages were clustered into five subtypes (**Figure 13A**), and M1/M2 marker genes were visualized via bubble plots (**Figure 13B**). The t-SNE and violin plots (**Figures 13C, D**) revealed that clusters 1 and 4 had higher M1 scores, whereas clusters 0, 2, and 3 had higher M2 scores. The distributions of M1 and M2 macrophages are shown via a t-SNE plot (**Figure 13E**). Bubble plots revealed that SPP1 was expressed mainly in clusters 0, 2, and 3 (**Figure 13F**), and violin plots revealed significantly greater expression of SPP1 in M2 macrophages than in M1 macrophages (P<0.001) (**Figure 13G**), suggesting that these M2 macrophages may have functional roles similar to those of previously reported SPP1+ macrophages. KEGG enrichment analysis (**Figure 13H**) revealed that cluster 0 was enriched in glycolysis/gluconeogenesis, carbon metabolism, cholesterol metabolism, amino acid biosynthesis, and the pentose phosphate pathways, indicating a metabolic subtype. Clusters 2 and 3 were enriched in the proteasome, ribosome, endoplasmic reticulum protein processing, phagosome, lysosome, and endocytosis pathways, suggesting a phagocytic innate immune subtype. Cluster 1 was enriched in the TNF, NF-κB, IL-17, Toll-like receptor, and chemokine signaling pathways, which are indicative of an inflammatory subtype. Cluster 4 was enriched in oxidative phosphorylation, NOD-like receptor signaling, B-cell receptor signaling, NF-κB signaling, and natural killer cell-mediated cytotoxicity pathways, representing an immune subtype. Overall, the KEGG results supported the annotation of clusters 1 and 4 as M1 macrophages involved in inflammation and immune activation, whereas clusters 0, 2, and 3 presented M2-like characteristics related to metabolism and phagocytic innate immunity (39, 40).

#### 3.10.4 Cell communication and molecular docking

**Figure 14:**
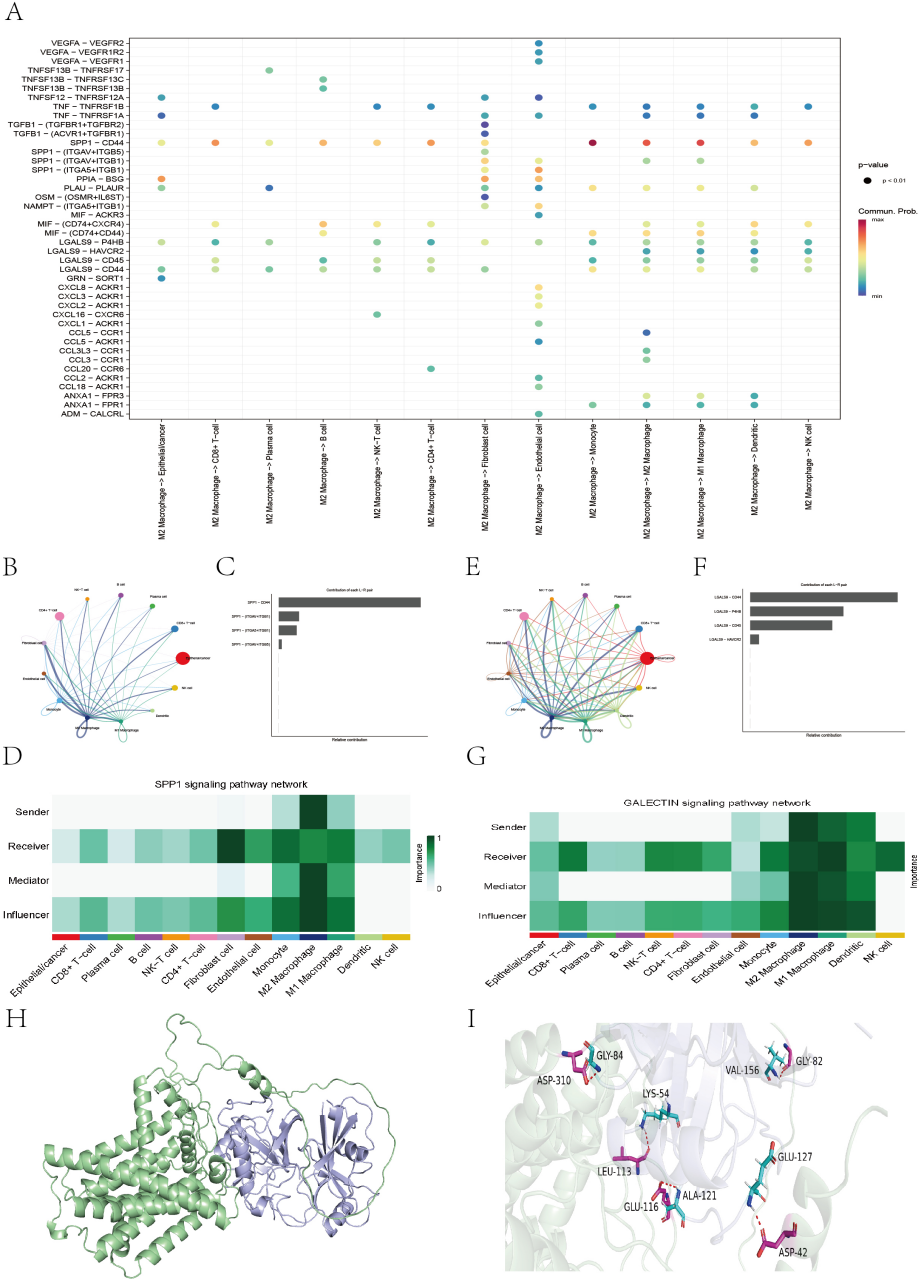
Cell-cell communication analysis of M2 macrophages and molecular docking of SPP1–CD44. **(A)** Bubble plot illustrating the interactions between M2 macrophages and other cell types mediated by secreted proteins. **(B, E)** Network diagram revealing the interaction relationships among different cell types based on SPP1 or GALECTIN signaling. **(C, F)** Bar chart showing the contribution of cell communication mediated by the binding of SPP1 or LGALS9 with various receptors. **(D, G)** Heatmap displaying the roles of different cell types in the SPP1 or LGALS9 signaling network, including senders, receivers, mediators, and influencers. **(H, I)** The protein structure diagram showing the molecular docking results of the SPP1 protein with the CD44 protein.

We specifically analyzed M2 macrophages as the signaling source, examining their outgoing interactions via secreted proteins and corresponding ligand–receptor pairs (**Figure 14A**). Taking the SPP1–CD44 pair as an example, we found that M2 macrophages acted as secreting cells and established strong connections with B cells, CD4+ T cells, CD8+ T cells, and NK cells—through SPP1–CD44 binding (**Figures 14B–D**). Similarly, in the GALECTIN signaling pathway, M2 macrophages serve as the primary outgoing signal source and interact with various immune cells—through the LGALS9–CD44 ligand–receptor pair (**Figures 14E–G**). To further explore the protein–protein interaction between SPP1 and CD44, we conducted molecular docking analysis. The optimal binding conformation exhibited a binding free energy of −11.0 kcal/mol. As shown in the surface model (**Figures 14H, I**), the proteins interact through hydrogen bonds formed by key amino acid residues, which increase the stability of the protein complex. Overall, the strong and stable binding between SPP1 and CD44 suggests that future therapeutic strategies could involve small molecules designed to block their interaction, potentially enhancing the efficacy of cancer immunotherapy.

### 3.11 Validation of model gene expression

**Figure 15:**
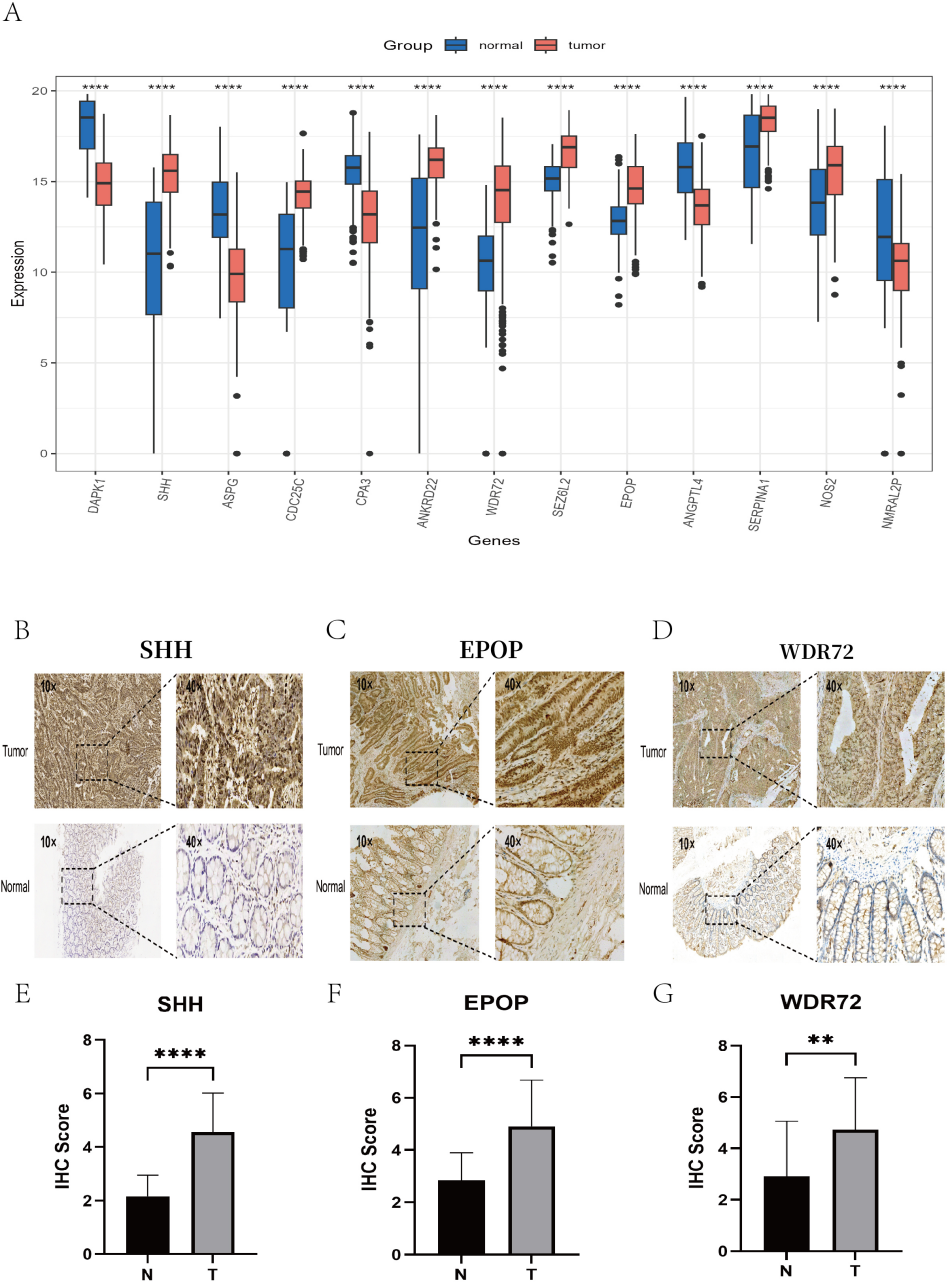
Validation of key gene expression via the HPA database and immunohistochemistry experiments. **(A)** Box plots showing the differential expression levels of the 13 key genes between normal tissues (blue) and colon cancer tissues (red). **(B–D)** Representative IHC images illustrating the protein expression of the key genes SHH, EPOP, and WDR72 in colon cancer tissues (top) and adjacent normal tissues (bottom), with each set displaying staining results at 10× and 40× magnifications. **(E–G)** Box plots presenting the differences in the IHC scores of the SHH, EPOP, and WDR72 proteins between colon cancer and corresponding normal tissues. ** P < 0.01, **** P < 0.0001.

We utilized immunohistochemistry (IHC) data from the Human Protein Atlas (HPA) database to validate the expression of prognostic model genes in tumor and normal tissues. In addition, we conducted independent IHC experiments for three genes—EPOP, WDR72, and SHH—which have rarely been validated by immunohistochemistry in previous studies of colorectal cancer. The IHC results from the HPA database largely aligned with our mRNA-level findings (**Figure 15A**). Specifically, CDC25C, ANKRD22, SEZ6L2, SERPINA1, and NOS2 were overexpressed in colon cancer tissues compared with normal tissues. In contrast, DAPK1, ASPG, and CPA3 were more highly expressed in normal tissues than in tumor tissues. Notably, no significant difference was detected in ANGPTL4 expression between colorectal cancer and normal tissues (**Supplementary Figures 11**, **12**). Our independent IHC experiments further confirmed that the IHC scores of SHH (4.56 ± 1.46 vs. 2.16 ± 0.79, p < 0.0001), EPOP (4.91 ± 1.76 vs. 2.84 ± 1.05, p < 0.0001), and WDR72 (4.73 ± 2.03 vs. 2.91 ± 2.15, p < 0.01) were significantly greater in tumor tissues than in normal tissues. (**Figures 15B–G**).

## 4 Discussion

In this study, we constructed a novel risk model based on ferroptosis and lipid metabolism in CC to predict the survival probability of CC patients and to provide insights for further research on the roles of ferroptosis and lipid metabolism in CC. The model consists of 13 genes: CPA3, NMRAL2P, CDC25C, SHH, DAPK1, ANKRD22, SERPINA1, ASPG, WDR72, SEZ6L2, NOS2, EPOP, and ANGPTL4. K–M curves revealed statistically significant differences in survival probability between the high-risk and low-risk groups across both the TCGA cohort and multiple GEO validation cohorts (P < 0.05). Multivariate Cox regression analysis suggested that the risk score derived from the model is an independent prognostic factor, irrespective of sex, age, and tumor TNM stage (P < 0.001). To increase predictive accuracy, we integrated the risk score with clinical and pathological features to construct a nomogram. Calibration curves for 1-, 3-, and 5-year survival, ROC curves, decision curve analysis (DCA), and the C-index consistently demonstrated the robustness of the nomogram in prognostic prediction, outperforming other clinical variables such as sex, age, and TNM stage. The immunohistochemistry (IHC) images of eight genes from the HPA database were consistent with our bioinformatics findings. Additionally, we collected 30 pairs of CC tissues and adjacent normal tissues for IHC experiments. The results revealed differential expression of SHH, WDR72, and EPOP between tumor and adjacent normal tissues (P < 0.05), which aligned with our expected findings. In conclusion, our model serves as a valuable prognostic tool for CC patients and holds promise as a potential therapeutic target for future CC treatment strategies.

CPA3, a member of the zinc metalloprotease carboxypeptidase family released by mast cells (41), may be involved in the inactivation of venom-associated peptides and the degradation of endogenous proteins (42). Although CPA3 has been extensively studied in asthma (43), COPD (44), and COVID-19 (45), its role in cancer remains largely unexplored. CDC25C is a key cell cycle regulator and a critical protein for G2/M transition and mitotic entry (46), potentially serving as a therapeutic target for CC patients and aiding in treatment decision-making (47). SHH, a secreted protein of the Hedgehog (HH) family, has been reported to promote endothelial cell growth, migration, and angiogenesis (48). Ghorbaninejad et al. (49) found that SHH signaling inhibitors exert anti-inflammatory effects on intestinal epithelial cells and maintain epithelial characteristics by restricting EMT induction. DAPK1 is a calmodulin-regulated, cytoskeleton-associated serine/threonine kinase (50). Wang et al. (51) reported that DAPK1 may act as an oncogene in gastric cancer (GC), promoting the invasion and migration of GC cells. The role of SERPINA1 in colorectal cancer (CRC) progression remains controversial. Some studies (52, 53) have shown that increased expression of SERPINA1 is associated with advanced disease, lymph node metastasis, and poor prognosis. However, others have reported that SERPINA1 may act as a protective factor in CRC (54), with its downregulation linked to the recurrence and distant metastasis of colon adenocarcinoma (55). ANKRD22, according to Yin et al. (56), is a novel tumor-associated gene in non-small cell lung cancer (NSCLC) that promotes tumor progression by upregulating E2F1 transcription and enhancing cell proliferation. However, the role of ANKRD22 in CRC has rarely been studied. ASPG is involved in the degradation of the nonessential amino acid asparagine. ASPG has also been identified as a prognostic biomarker for colon cancer in several studies (57, 58). SEZ6L2 has been identified as a potential prognostic biomarker (59) and therapeutic target (60) in CRC. An et al. (61) reported that the knockout of SEZ6L2 inhibits tumor growth in CRC by promoting caspase-dependent apoptosis. NOS2, a key inflammatory enzyme responsible for nitric oxide synthesis, is highly expressed in CRC according to Li et al. (62). However, NOS2 was shown to inhibit tumor growth and induce tumor cell death both *in vitro* and *in vivo*, potentially through suppression of the NF-κB pathway. Numerous studies (54, 63) have shown that ANGPTL4 is involved in tumor metastasis and angiogenesis. One study (64) reported that ANGPTL4 encodes a secreted glycoprotein that promotes angiogenesis and inhibits ferroptosis. Liefke et al. (65) reported that EPOP (C17orf96) participates in transcriptional processes similar to those of the oncogene MYC, suggesting that EPOP may be expressed in human cancer cells and exhibit oncogenic potential. They further demonstrated that EPOP expression in colon cancer is positively correlated with proliferation characteristics. NMRAL2P is a lncRNA reported to be significantly downregulated in CRC tissues compared with adjacent normal tissues (66). Wu et al. (67) found that NMRAL2P is amplified in gallbladder cancer (GBC) cells and is associated with invasiveness and epithelial–mesenchymal transition (EMT), making it a potential therapeutic target. Zhang et al. (68) reported that WDR72 expression was significantly elevated in colorectal cancer (CRC) patients with distant metastasis, lymph node involvement, and advanced clinical stage and was strongly associated with poor prognosis. This finding was largely consistent with our bioinformatic results, which revealed that high WDR72 expression was significantly correlated with lymph node metastasis (P<0.01), distant metastasis (P<0.01), and poor overall survival (P = 0.014) (**Supplementary Figures 13A, B**). Moreover, GSEA indicated that WDR72 is closely associated with oncogenic pathways, hypoxia, angiogenesis, proliferation, metastasis, and invasion (**Supplementary Figures 13C–G**). Stratified clinical analysis further revealed that the survival difference between the high and low WDR72 expression groups was particularly significant among elderly patients (≥60 years, P = 0.012), females (P<0.001), and those with T3/4 stage tumors (P = 0.009) (**Supplementary Figures 14**, **15**). In summary, WDR72 may serve as a novel therapeutic target for colon cancer (CC) in the future; however, its functional role and related pathways in CRC remain to be validated through further *in vivo* and *in vitro* experiments.

This study also revealed strong associations among lipid metabolism, ferroptosis, risk score, and prognosis. As demonstrated in earlier analyses, both NMF and CMS classifications revealed that subtypes with poorer prognoses tended to have higher risk scores. In our prognostic model constructed using 13 core genes, a higher risk score was consistently correlated with worse prognosis across both the TCGA training cohort and multiple GEO validation datasets. Moreover, the risk score was significantly negatively correlated with ferroptosis and nearly all lipid metabolism pathway activities. In the NMF classification, the C3 subtype presented lower risk scores and higher activity scores for ferroptosis, lipid oxidation, and glycerolipid metabolism, with a trend toward increased fatty acid metabolism activity. These findings collectively indicate that increased activity of lipid metabolism and ferroptosis pathways may be associated with lower risk scores and better prognosis. For single-cell analysis, we divided tumor cells into two groups on the basis of ferroptosis–lipid metabolism (FRLM) activity. Cells with low FRLM scores were enriched mainly in pathways such as epithelial–mesenchymal transition, angiogenesis, apical junction, and apical surface, further suggesting that tumor cells with low ferroptosis–lipid metabolism activity are associated with greater invasive and metastatic potential, which is linked to poorer outcomes.

We further explored the potential molecular mechanisms underlying the poor prognosis of the high-risk group through multiple enrichment analysis methods. First, our findings revealed that hypoxia, tumor angiogenesis, and the VEGF_VEGFR interaction pathway were positively correlated with the risk score. Previous study has reported that the hypoxic tumor microenvironment promotes abnormal angiogenesis, thereby contributing to tumor progression (69). GSEA indicated that the high-risk group was significantly enriched in processes such as epithelial–mesenchymal transition (EMT), pseudopod chemotaxis, and cell migration in colorectal cancer. EMT is characterized by the loss of epithelial traits and the gain of mesenchymal features, endowing cells with migratory and metastatic capabilities (70) and further supporting the aggressive and invasive features of the high-risk group. Additionally, the high-risk group was found to be involved in the synthesis of extracellular matrix (ECM) components, including collagen (71), proteoglycans (72), glycosaminoglycans (72), and chondroitin sulfate (73), as well as in ECM-related pathways such as ECM-receptor interactions (74), focal adhesion (75), laminin interactions (76), L1CAM interactions (77), and integrin signaling (78). In contrast, GSEA of the hallmark and C2 gene sets revealed that the low-risk group was enriched mainly in biological processes such as fatty acid metabolism, mitochondrial fatty acid β-oxidation, oxidative phosphorylation, peroxisome function, mitochondrial activity, the respiratory electron transport chain, and bio-oxidation. These biological processes have been widely reported to potentially induce ferroptosis (79–81). Taken together, these findings suggest that the high-risk group is characterized by increased invasive potential, which is associated with ECM remodeling and interactions and linked to tumor progression and metastasis. The low-risk group may benefit from increased lipid metabolism and oxidative phosphorylation, which may correlate with ferroptosis and tumor suppression.

We further investigated differences in the TME between the high- and low-risk groups. According to the transcriptomic analyses, the low-risk group was associated with increased levels of plasma cells, CD4+ memory T cells, M1 macrophages, and follicular helper T cells. In contrast, the high-risk group presented increased infiltration of M0 macrophages, fibroblasts, and resting NK cells. It’s suggested that the high-risk group may have a more immunosuppressive microenvironment. For single-cell analysis, we classified macrophages into the conventional M1 (classically activated or proinflammatory) and M2 (alternatively activated or anti-inflammatory) subtypes (82). M2 macrophages, often referred to as tumor-associated macrophages (TAMs) (39), show significantly greater expression of SPP1 than M1 macrophages do, which is consistent with previous findings (83, 84). Studies have demonstrated that SPP1-high TAMs represent a distinct immunosuppressive subset and are positively correlated with EMT markers (85, 86). Zhang et al. reported that SPP1 promotes M2 polarization and suppresses T-cell activation in lung cancer, thereby contributing to immune evasion (87), which further supports the immunosuppressive role of M2 macrophages. Yang et al. reported that iron overload can promote M1 polarization and that reactive oxygen species (ROS) produced during ferroptosis may also favor M1 macrophage polarization (88). This may explain why the low-risk group, which exhibited increased ferroptosis–lipid metabolism activity, also presented increased M1 macrophage infiltration according to CIBERSORT analysis. Cell–cell communication analysis revealed that M2 macrophages act as secretory cells, closely interacting with immune cells such as B cells, CD4+ T cells, CD8+ T cells, and NK cells via SPP1-CD44 and LGALS9-CD44 ligand–receptor pairs. Fan et al. reported that in the GALECTIN signaling pathway, LGALS9 secreted by CTSE+ tumor cells activated CD44 on MARCO+ TAMs, promoting tumor progression and immune escape (89). Previous studies also reported that TAMs characterized by exclusive SPP1 expression could potentially activate fibroblasts and induce T-cell exhaustion through the SPP1-CD44 interaction, thereby remodeling the metastatic lymph node microenvironment (90). He et al. further demonstrated that targeting the SPP1-CD44 axis restored T-cell function and enhanced anti-PD-1 therapy efficacy *in vitro*, significantly reducing tumor burden (91), suggesting that SPP1-CD44 may be a promising therapeutic target to enhance antitumor immunity. Moreover, we observed significantly elevated scores for T-cell dysfunction and exclusion (TIDE), cancer-associated fibroblasts (CAFs), and myeloid-derived suppressor cells (MDSCs) in the high-risk group, indicating more frequent immune escape, potentially due to greater stromal infiltration and enhanced immunosuppressive properties in the TME. Conversely, the low-risk group had higher microsatellite instability (MSI) scores and improved immunophenoscore (IPS), indicating greater tumor immunogenicity and a greater likelihood of responding to immune checkpoint inhibitors. This conclusion was further validated in multiple immunotherapy cohorts downloaded from the TIGER database. In summary, our prognostic model is closely related to immune infiltration in CC and has predictive value for immunotherapy efficacy in CC patients.

This study has several limitations. First, since the concept of ferroptosis was introduced in 2012 (92), research in this field has rapidly expanded, and multiple ferroptosis-related prognostic models have been proposed. The inclusion of different core genes in these models may lead to varying predictive outcomes. Like most bioinformatics studies, our analysis was retrospective, based on TCGA and GEO datasets, and lacked clinical validation. Although our model shows potential in predicting immunotherapy response in colon cancer, the validation cohorts were mainly from melanoma, bladder cancer, and gastric adenocarcinoma, with no colon cancer immunotherapy data, which may limit generalizability. In addition, we acknowledge that the lack of validation using independent institutional or multicenter clinical datasets may affect the robustness of our findings. At present, comprehensive colorectal cancer cohorts containing detailed clinicopathological, genomic, and immunotherapy-related information remain limited. However, we are actively establishing a biobank and conducting prospective clinical studies, continuously collecting samples to enable future validation of our model in real-world clinical settings. In addition, although we integrated single-cell transcriptomic data to identify major cell types, malignant epithelial cells, and macrophage subtypes, mechanistic insights at the single-cell level remain limited due to data availability and computational constraints. For example, we were unable to fully delineate, through a continuous chain of evidence—from gene expression and signaling activation to cell–cell communication, phenotypic transition, and functional validation—how the model genes function at the cellular level. Moreover, we were not able to connect large-scale immune subtypes with microscopic cellular interactions, which limited the full potential of our multi-omics integration. Future studies combining spatial transcriptomics, single-cell multi-omics, and functional experiments will be essential to elucidate the relationships between genes and cells, as well as the intercellular interactions within the tumor microenvironment. Finally, our study mainly established a correlative rather than mechanistic link between ferroptosis, lipid metabolism, and tumor biology. Although our analyses demonstrated that ferroptosis–lipid metabolism activity is closely associated with immune landscape heterogeneity and clinical outcomes in colorectal cancer, direct experimental evidence is still lacking to explain the underlying biological mechanisms. From a statistical perspective, this retrospective analysis—based primarily on existing public datasets—can only reveal associations rather than causal relationships between variables. Therefore, future studies incorporating a variety of *in vitro* and *in vivo* functional experiments are urgently needed to elucidate how key genes mechanistically bridge ferroptosis and lipid metabolism, regulate the tumor immune microenvironment, and influence tumor cell behavior.

## 5 Conclusion

On the basis of ferroptosis and lipid metabolism, we constructed a risk model using 13 core genes that demonstrated strong prognostic value for colon cancer (CC) patients. Furthermore, this risk model reveals the heterogeneity of the immune landscape associated with ferroptosis and lipid metabolism and validates the potential of immunotherapy in treating patients with colon cancer, providing valuable guidance for future clinical practice and the implementation of personalized treatment strategies.

## Data Availability

Publicly available datasets were analyzed in this study. This data can be found here: http://www.ncbi.nlm.nih.gov/geo; https://xenabrowser.net/.
