# Supplementary material for: Integration of single-cell and bulk RNA-seq via machine learning to reveal ferroptosis- and lipid metabolism-driven immune landscape heterogeneity and predict immunotherapy response in colon cancer
Source: Front Immunol. 2025 Dec 5;16:1699079. doi: 10.3389/fimmu.2025.1699079 (PMC12714941; doi:10.3389/fimmu.2025.1699079)
Supplement: Supplementary file 19 [file Table4.docx]

**Differential expression analysis of molecular subtypes and enrichment analysis**

Differential expression analysis for the TCGA-GTEx colon cancer dataset (raw counts) was performed via the DESeq2 R package, with significant DEGs defined as those with adjusted P value < 0.05 and |log2FC| > 1.5. For the TCGA cohort with survival data (TPM format), DEGs between groups were identified via the limma R package with thresholds of adjusted P value < 0.05 and |log2FC| > 1; if necessary, the |log2FC| threshold was relaxed to 0.585.

DEGs were visualized via the tinyarray R package (version 2.4.2) through heatmaps, volcano plots, and Venn diagrams generated via the draw_heatmap, draw_volcano, and draw_venn functions, respectively. Gene Ontology (GO) and Kyoto Encyclopedia of Genes and Genomes (KEGG) enrichment analyses were performed using the quick_enrich function implemented in the tinyarray package (v2.4.2), which internally calls clusterProfiler (v4.14.4). Differentially expressed genes were first converted to Entrez IDs using org.Hs.eg.db (v3.19.1). KEGG enrichment was conducted with enrichKEGG function and GO enrichment with enrichGO function (ontology = “all”), using the default hypergeometric test algorithm. The background was defined as all genes successfully mapped to Entrez IDs in the dataset. Multiple testing correction was performed using the Benjamini–Hochberg false discovery rate (FDR) method, and terms with adjusted P value (FDR) < 0.05 were considered significant. Additionally, Metascape (https://metascape.org/) was used for supplementary GO and KEGG analyses to further explore biological functions(1).

**Gene set enrichment analysis (GSEA)**

In this study, GSEA was performed via the GSEA function from the clusterProfiler R package. The reference gene sets utilized were obtained from the "H" (hallmark gene sets) and "C2" (curated gene sets) collections within the msigdbr R package.

**Mutation profiles and copy number variation（CNV）**

We employed the TCGAmutations R package (version 0.3.0) and the maftools R package (version 2.22.0)(2) to download and extract mutation annotation format (MAF) files from the TCGA database, enabling a detailed investigation into the differences in the mutation landscape between high-risk and low-risk groups of colon cancer patients. We downloaded and processed CNV data via the TCGAbiolinks R package (version 2.34.0), aligning them to the reference genome hg38. GISTIC 2.0 (<https://broadinstitute.github.io/gistic2/>) was used to analyze the CNV data online. The function differential_CNV from the GeoTcgaData R package (version 2.6.0) was applied to identify genes with significant differential CNVs. Visualization of CNV results was performed via maftools and ggplot2 R packages.

**Assessment of immune microenvironmental characteristics**

On the basis of the RNA-seq data, we employed the CIBERSORT algorithm to estimate the infiltration levels of 22 immune cell types in the high- and low-risk groups. Specifically, the LM22 signature matrix was utilized, and the results were generated via 1,000 permutations to calculate the relative proportions of each immune cell subtype. Additionally, we applied multiple algorithms, including EPIC, MCPcounter, QUANTISEQ, TIMER, ESTIMATE and xCELL, implemented within the IOBR R package (version 0.99.0), to assess the tumor immune microenvironment comprehensively.

**Immunophenoscore analysis and tumor immune dysfunction and exclusion analysis**

Immunophenoscore (IPS) has been identified as an excellent predictor of immunotherapy sensitivity and is used to evaluate key determinants of tumor immunogenicity (3). The IPS profiles for the CC cohort were obtained from The Cancer Immunome Atlas (TCIA, <https://tcia.at/>). Higher IPS values indicate a better predicted response to immunotherapy with CTLA-4 or PD-1 monoclonal antibodies.

Tumor immune dysfunction and exclusion (TIDE, <http://tide.dfci.harvard.edu/>) integrates two mechanisms of tumor immune evasion, T-cell dysfunction and T-cell exclusion (4), and serves to assess the predictive value of the risk score in the context of immunotherapy. A higher TIDE score suggests a greater likelihood of immune escape and a lower probability of benefit from immunotherapy.

**Drug sensitivity analysis**

Chemotherapy and targeted drug responses were predicted for TCGA patients via the OncoPredict R package (version 1.2) based on the Genomics of Drug Sensitivity in Cancer (GDSC) database. The training dataset for OncoPredict was downloaded from <https://osf.io/c6tfx/>. Specifically, we used GDSC2_Expr (RMA normalized and log-transformed) and GDSC2_Res as the training data. The TCGA expression data were log2-transformed as log2(TPM + 1) for subsequent analyses. Batch correction between the training and testing matrices was performed via the standardize function. Ridge regression was applied to estimate the half-maximal inhibitory concentration (IC50) of each drug for individual patients.

**Gene set variation analysis**

The “h.all.v2023.2.Hs.symbols.gmt” file containing the HALLMARK gene sets was downloaded from the Molecular Signatures Database (MSigDB). Subsequently, GSVA enrichment analysis was performed on the cell subpopulations via the GSVA R package (version 2.0.4). The AverageExpression function was used to calculate the average gene expression across all cells within each subcluster.

**Pseudotime trajectory analysis**

Monocle2 (version 2.34.0) was used for pseudotime analysis to determine the differentiation trajectory of cellular development. After the UMI matrix was extracted from the Seurat object, a new Monocle object was created via the newCellDataSet function. The differentialGeneTest function was used to identify DEGs among various cell types for trajectory inference. Dimensionality reduction was performed via the reduceDimension function, followed by cell ordering via the orderCells function. Finally, the plot_cell_trajectory function was used to visualize the pseudotime trajectory and cell state transitions.

**Cell–cell communication analysis**

Cell-cell communication among immune subpopulations in colon cancer was analyzed via the CellChat R package (version 2.1.2), with the CellChatDB.human database used as a reference. The Seurat object was converted into a CellChat object (createCellChat function), with a focus on "Secreted Signaling" interactions. The communication probabilities were calculated with the computeCommunProb function, and the interaction network was aggregated via the aggregateNet function to generate a global signaling map. The contributions of individual ligand-receptor pairs were assessed via the netAnalysis_contribution function.

**The Human Protein Atlas (HPA)**

The Human Protein Atlas (HPA) (5,6) is a freely accessible database that provides protein expression data for more than 40 types of normal human tissues and cancer tissues via immunohistochemistry. To validate the findings of our bioinformatics analysis, we searched the HPA database for immunohistochemical images of the corresponding genes included in our prognostic model.

**References**

1. Zhou, Zhou, Pache, Chang, Khodabakhshi, Tanaseichuk, et al. Metascape provides a biologist-oriented resource for the analysis of systems-level datasets. Nat Commun April 3, 2019; 10 :1523. doi: 10.1038/s41467-019-09234-6

2. Mayakonda, Lin, Assenov, Plass, Koeffler. Maftools: efficient and comprehensive analysis of somatic variants in cancer. Genome Res November 2018; 28(11) :1747–56. doi: 10.1101/gr.239244.118

3. Charoentong, Finotello, Angelova, Mayer, Efremova, Rieder, et al. Pan-cancer Immunogenomic Analyses Reveal Genotype-Immunophenotype Relationships and Predictors of Response to Checkpoint Blockade. Cell Rep January 3, 2017; 18(1) :248–62. doi: 10.1016/j.celrep.2016.12.019

4. Jiang, Gu, Pan, Fu, Sahu, Hu, et al. Signatures of T cell dysfunction and exclusion predict cancer immunotherapy response. Nat Med October 2018; 24(10) :1550–8. doi: 10.1038/s41591-018-0136-1

5. Uhlen, Zhang, Lee, Sjöstedt, Fagerberg, Bidkhori, et al. A pathology atlas of the human cancer transcriptome. Science August 18, 2017; 357(6352) :eaan2507. doi: 10.1126/science.aan2507

6. Uhlén, Fagerberg, Hallström, Lindskog, Oksvold, Mardinoglu, et al. Proteomics. Tissue-based map of the human proteome. Science January 23, 2015; 347(6220) :1260419. doi: 10.1126/science.1260419
