# Supplementary material for: Integration of single-cell and bulk RNA-seq via machine learning to reveal ferroptosis- and lipid metabolism-driven immune landscape heterogeneity and predict immunotherapy response in colon cancer
Source: Front Immunol. 2025 Dec 5;16:1699079. doi: 10.3389/fimmu.2025.1699079 (PMC12714941; doi:10.3389/fimmu.2025.1699079)
Supplement: Supplementary file 20 [file Table5.docx]

RPL8

IREB2

ATP5MC3

CS

EMC2

ACSF2

NOX1

CYBB

NOX3

NOX4

NOX5

DUOX1

DUOX2

G6PD

PGD

VDAC2

PIK3CA

FLT3

SCP2

TP53

ACSL4

LPCAT3

NRAS

KRAS

HRAS

TF

TFRC

TFR2

SLC38A1

SLC1A5

GLS2

GOT1

CARS1

ALOX5

KEAP1

HMOX1

ATG5

ATG7

NCOA4

ALOX12

ALOX12B

ALOX15

ALOX15B

ALOXE3

PHKG2

ACO1

G6PDX

ULK1

ATG3

ATG4D

BECN1

MAP1LC3A

GABARAPL2

GABARAPL1

ATG16L1

WIPI1

WIPI2

SNX4

ATG13

ULK2

SAT1

EGFR

MAPK3

MAPK1

BID

ZEB1

DPP4

CDKN2A

PEBP1

SOCS1

CDO1

MYB

MAPK8

MAPK9

CHAC1

MAPK14

LINC00472

PRKAA2

PRKAA1

ELAVL1

BAP1

ABCC1

MIR6852

ACVR1B

TGFBR1

EPAS1

HILPDA

HIF1A

IFNG

ANO6

LPIN1

HMGB1

TNFAIP3

TLR4

ATF3

ATM

YY1AP1

EGLN2

MIOX

TAFAZZIN

MTDH

IDH1

SIRT1

FBXW7

PANX1

DNAJB6

BACH1

LONP1

CD82

IL1B

CTSB

POR

CYB5R1

ELOVL5

FADS1

PTEN

NR1D1

NR1D2

TBK1

IL6

USP7

miR-182-5p

miR-378a-3p

ATF4

AQP3

AQP5

AQP8

LINC00618

MT1DP

PEX10

AGPAT3

PEX12

CHP1

GPAT4

BRPF1

OSBPL9

INTS2

MMD

CYP4F8

MLLT1

TTPA

GRIA3

EPT1

POM121L12

LIG3

AEBP2

AGPS

CDCA3

PEX2

PEX6

TIMM9

DCAF7

LCE2C

FAR1

PHF21A

SMAD7

LYRM1

AMN

PEX3

MTCH1

ACADSB

PVT1

hsa_circ_0008367

SLC39A14

MAP3K11

GSK3B

BRD7

SLC25A28

MFN2

SLC11A2

ZFAS1

TSC1

TGFB1

SNCA

SIRT3

CGAS

STING1

HDDC3

MIR761

MDM2

MDM4

MIR214

DLD

WWTR1

PRKCA

LGMN

SMPD1

MYCN

IFNA1

IFNA2

IFNA4

IFNA5

IFNA6

IFNA7

IFNA8

IFNA10

IFNA13

IFNA14

IFNA16

IFNA17

IFNA21

SMG9

PPARG

MIR335

SNX5

PAQR3

MICU1

TOR2A

MIR375

MAP3K14

CircKDM4C

MIR324

QSOX1

MIB2

CLTRN

KLF2

MIR5096

HOTAIR

H19

FOXO4

YTHDC2

DDR2

SLC39A7

TRIM46

ACSL1

KDM5A

TRIM21

DPEP1

CYGB

IDO1

GSTZ1

GJA1

SLC7A11

PGRMC1

CIRBP

circPSEN1

USP11

YAP1

MIR135B

TRIM26

NDRG1

MIR302A

ASMTL-AS1

FADS2

PIEZO1

LIFR

PTPN6

MIR15A

EGR1

ADAM23

ARHGEF26-AS1

CPEB1

COX4I2

lncRNA AABR07017145.1

TIMP1

KDM6B

METTL14

MIB1

KDM5C

MEG3

CCDC6

CFL1

MIR539

KMT2D

GPX4

AKR1C1

AKR1C2

AKR1C3

RB1

HSPB1

HSF1

GCLC

NFE2L2

SQSTM1

NQO1

FTH1

MUC1

SLC3A2

MT1G

SLC40A1

CISD1

FANCD2

FTMT

HSPA5

HELLS

SCD

SRC

STAT3

PML

MTOR

NFS1

TP63

CDKN1A

MIR137

ENPP2

FH

CISD2

MIR9-1

MIR9-2

MIR9-3

CBS

ISCU

ACSL3

OTUB1

CD44

LINC00336

BRD4

PRDX6

MIR17

SESN2

NF2

ARNTL

JUN

CA9

TMBIM4

PLIN2

MIR212

Fer1HCH

AIFM2

LAMP2

ZFP36

PROM2

CHMP5

CHMP6

CAV1

GCH1

DAZAP1

PIR

FTL

HCAR1

SLC16A1

RRM2

NR4A1

RPTOR

SREBF1

SREBF2

FZD7

P4HB

NT5DC2

BCAT2

PLA2G6

MIR424

PARK7

FXN

SUV39H1

ATF2

ACOT1

ALDH3A2

STK11

FNDC5

CircIL4R

CDH1

NEDD4L

BRD2

BRD3

BRDT

DECR1

GLRX5

NCOA3

NR5A2

PANX2

RHEBP1

TFAP2A

CP

ARF6

GDF15

ABHD12

PPP1R13L

TFAM

KDM3B

RNF113A

AHCY

circ-TTBK2

MIR522

IDH2

PPARA

NOS2

SIAH2

RELA

VDR

NEDD4

PRDX1

AR

MTF1

COPZ1

NUPR1

USP35

NEAT1

PARP1

PARP2

PARP3

PARP4

PARP6

PARP8

PARP9

PARP10

PARP11

PARP12

PARP14

PARP15

PARP16

PDSS2

TXN

SENP1

OIP5-AS1

MIR190A

FGF21

CREB1

CREB3

CREB5

MIR130B

BEX1

ASAH2

FABP4

AKT1S1

MLST8

TYRO3

SIRT6

TMSB4X

TMSB4Y

KIF20A

ECH1

circRHOT1

ETV4

MEG8

VCP

circ_0007142

RBMS1

KDM4A

MGST1

circKIF4A

miR-7-5p

circ_0067934

MPC1

CHMP1A

CAMKK2

SOX2

SRSF9

PROK2

MIR4443

SIRT2

circRNA1615

MIR27A

MIR670

MEF2C

EZH2

PEDS1

ADAMTS13

CDC25A

CircFNDC3B

PPARD

ENO3

LCN2

MARCHF5

TRIB2

DHODH

MIR545

PDK4

CircPVT1

MIR9-3HG

ADIPOQ

circDTL

mmu_circRNA_0000309

PTPN18

ABCC5

CISD3

MS4A15

FURIN

circRHBG

GALNT14

KLHDC3

LINC01833

circGFRA1

MAPKAP1

PRR5

RICTOR

GSTM1

TERT

circ0097009

TMEM161B-DT

circEPSTI1

MIR18A

RARRES2

PTGS2

HEPFAL

MIR7-3HG

GABPB1-AS1

SCARNA5

SOD2-OT1

TMEM164

LINC01672

RAB4B-EGLN2

TRIM7

PCBP2

VDAC3

BDNF-AS

PCBP1

TMX2-CTNND1

CERNA3

XIST

MAP1LC3B

PRNP

MAP1LC3C

STEAP3

EMSLR

GAS5

ZFPM2-AS1

MSC-AS1

MIR4435-2HG

HULC

MIR93

LINC01614

SNHG16

LINC01224

CYTOR

PLXNB2

TUG1

MIR494

SNHG7

TRC-GCA24-1

LINC00665

TRA-TGC7-1

LINC-ROR

SNORD46

EP300

SNHG14

SP1

MIR133B

KCNQ1OT1

FDFT1

TXNRD1

ACSL5

TMPO-AS1

SNORD118

LINC01503

MYC

TIGAR

TRU-TCA1-1

LINC01857

SNORD15A

TNF

MIR497

MIAT

PDIA4

DANCR

LINC01063

SNHG1

CTH

GSS

HMGCR

SLC39A8

COQ2

GCLM

ACSL6

SAT2

GRIK1-AS2

PKM

BMAL1

MIRLET7C

MIR4668

MIR758

RBMX

HNRNPC

MIR100HG

CCDC144NL-AS1

VDAC1

ARRDC1-AS1

HMGCS1

H1-3

MIR129-1

LDHA

MIR429

FBLN1

H2AX

GPX3

SND1

PAX8-AS1

ELF3-AS1

LINC01094

PSMA3-AS1

LINC02605

OTUD6B-AS1

HSPA8

HCG18

YY1

CUL4B

LASTR

CBSLR

HOTAIRM1

SNAI3-AS1

LIPE-AS1

LINC01871

SQLE

SOCS2

YTHDF2

EMP1

ACTB

KRT18

ALB

KRT14

EEF1A1

KRT16

KRT6B

MIR23A

KTN1-AS1

LINC00462

PGR-AS1

CASC9

DLEU1

LINC00324

MIR193A

LINC00205

SNHG10

EIF3J-DT

HNRNPA2B1

GDNF-AS1

RMST

SFTA1P

SLCO4A1-AS1

LINC02541

APOC1

MIR320A

SNHG17

ENO1

HOXB-AS3

MIR338

PELATON

C1RL-AS1

LINC02446

LINC01655

IRF1

DARS1-AS1

G3BP1

MIR142

HNRNPD

RNU6-1

MILIP

SREBF2-AS1

HSPA9

LINC00520

ZNF667-AS1

ASCL4

MAPT-AS1

VIM

ATP5F1A

ATP5F1B

PCBP3

IGKC

IGHG4

NAT10

MIR655

FSCN1

IFITM9P

XRCC6

PTBP1

CCT3

SKP2

MYLK-AS1

MIR181D

MIR193BHG

MIR373

LUCAT1

SCARNA22

NCK1-DT

MIR507

CKB

H2AC1

SLC25A5-AS1

HNF4A

ADCY10

ATL1

APOL3

CRMA

SSBP1

WTAP

C5orf64

MIR155HG

LINC00968

ERBB2

CTNNB1

YWHAE

NR1H4

BGN

RACK1

RPL7

RPS3A

TRIM54

SFTA3

CD300LD-AS1

MIR30E

MIR122

ZEB1-AS1

SLC7A11-AS1

MIR372

MIR4465

TMEM105

MIR155

PTGES2-AS1

LINC01436

MIR1273C

PRKCB

IRF8

HAMP

MBTPS1

HDLBP

SLC47A1

LRFN5

PNO1

LACTB

TRIM69

PRRX2

PRMT9

FOXC2-AS1

MIR362

MIR15B

CASC8

PSEN1

EIF2AK3

GGT1

SPARC

GPD2

MAT2A

USP14

FOXC1

STYK1

UBE2G2

UBE2J2

CERS6

NKAP

KLF11

MARCHF6

MIR19A

LINC00511

LINC01139

LOC654780

DUXAP8

MIR29A

NFKB1

PHGDH

CASK

KAT5

KDM1A

ALDH1A1

HNRNPA1

KRT1

CYP24A1

KRT5

SLC11A1

ARG2

ETFA

MCM5

STUB1

DKK1

KRT17

LTBP2

CSTA

KRT10

KRT6A

P4HA1

SHC1

SNAI2

TAGLN

TUBA1B

FFAR2

GRK4

KRT2

KRT9

CPLX2

ISYNA1

SLC6A14

SQOR

DCD

RETREG1

PM20D1

H4C1

IGHG1

SERPINB12

MITD1

NOL10

H2BC12

MYH16

LINC00092

MIR491

LINC00958

A2M-AS1

ADAMTS9-AS1

IRAG1-AS1

MIR147A

MIR587

DDX11-AS1

IGKV2D-40

IGKV3D-20

LINC01508

MIR365B

VTRNA1-1

IGKV2-40

LINC01836

PGM5P3-AS1

LINC02154

SOD1

BIRC5

SPHK1

XBP1

CELF2

EFEMP1

PLTP

WDR5

FGF4

CDCA7

SGK2

ANKRD1

ELOC

ISCA2

SYVN1

UCHL5

USF2

MAFG

CUL9

SHARPIN

HRNR

YY2

LINC02908

LINC02913

MIR10B

MIR30B

MIR370

MIR431

MIR217

MIRLET7B

SNHG6

HAGLROS

ILF3-DT

MIR1-2

BOLA3-DT

INKA2-AS1

LINC02716

MIR3200

SNORD95

LINC00616

LINC01579

MIR1287

SNHG21

LINC02080

FOXD1-AS1

LINC01956

PWAR1

ESR1

INSR

MMP2

APP

CD36

TYMS

HSPG2

SLC12A5

ALDH7A1

NAMPT

PSAP

ALAS2

CD59

COX4I1

DDB1

ERCC6

GSTM3

ITGB5

OGDH

SLC25A13

SPTLC1

TALDO1

APEX1

ATIC

MDH1

CTNND1

PLAUR

UBE2D3

ACTR2

ADAM15

ADSS2

CCL5

CUL4A

MYOF

NFE2L1

PPIF

RBX1

RUNX3

SETDB1

TECR

ANXA3

EIF5

FOSL2

H1-4

HMGB3

LTBP4

METTL3

NGLY1

NPEPPS

PCSK6

TMED10

CTSE

SEMA5A

TIMP2

H1-2

H1-5

IGF2BP1

PARL

RPA3

TES

ABCB10

COX5B

DNAJC7

SCARA5

TNFRSF19

DCAF8

ELOB

FKBP2

NSUN5

TBCA

TCEA1

ACTR1B

H1-1

MRPL19

PFDN6

COX7A1

CST1

IBA57

TRIM6

UFL1

ABCF3

CST4

PCDH20

SH3BGRL3

WDR76

COMMD10

HIVEP3

METTL16

SPCS2

FOXN4

MAL2

ZNF8

H1-10

SERF2

KPRP

LINC00473

MIR132

MIR326

MIR499A

CARMN

LINC-PINT

MIR31HG

MIR144

LINC00460

LINC00239

LINC00996

ARGLU1-DT

DNAJC3-DT

HEIH

ITGB2-AS1

LINC00673

MIR552

LINC00449

ZBTB40-IT1

MIR4735

MIR6825

MIR498

AKT1

DNMT1

CDH2

SMARCB1

ABCB6

LTF

FMR1

MEN1

PYCR1

S100A4

ADIPOR1

CX3CL1

JAM3

LGALS1

REST

CDH4

RHOT1

SLC25A10

YME1L1

CSRP2

IGF2BP3

SEC24B

SLC27A5

HPCAL1

STEAP1

STOML2

GABPB1

RBM15

SPINK2

TFCP2L1

BNC1

FBXO31

SLIRP

ZSCAN25

C19orf12

ALKBH5

ZNF350

SCGB1D2

TMED8

MIR127

LINC01558

MIR106A

LINC00240

LINC00342

BBOX1-AS1

TMEM254-AS1

OGFRP1

PWAR4

C8orf76

NOTCH2

ATF6

TRPV4

CEBPA

FPR2

HMGCL

BSG

FXR1

SLC27A4

TTBK2

LASP1

AIM2

CEP290

ITGB8

MELTF

EIF3H

FDX1

STC1

PCDH7

HLF

PGAM5

HECTD3

RNF182

TRIM59

UTP11

PCDHB14

ATXN8OS

TCL6

MIR17HG

CACNA1G-AS1

MIR29C

SNORD104

LINC00963

CERS6-AS1

MIR501

TRAM2-AS1

MIR223HG

TDRKH-AS1

PDGFRB

PCSK9

PIM1

UBE2N

ANO1

IGF2BP2

SETD7

FABP1

CDK14

ME1

ALG3

ARPC1A

RSL1D1

SOX15

MEX3A

OLMALINC

AGAP2-AS1

MIR1231

MIR509-1

MIR509-3

MIR509-2

MIR3938

ITGA6

ADAMTS9-AS2

ATP2A1-AS1

LINC00886

SOD2

ZC3H13

FGD5-AS1

MALINC1

GASAL1

RSF1-IT2

CASC2

FAM106A

LINC00242

LMO7DN

MIR152

PCAT1

PGM5-AS1

LINC03007

PXN-AS1

SCAT1

LINC00680
