# Supplementary material for: Integration of single-cell and bulk RNA-seq via machine learning to reveal ferroptosis- and lipid metabolism-driven immune landscape heterogeneity and predict immunotherapy response in colon cancer
Source: Front Immunol. 2025 Dec 5;16:1699079. doi: 10.3389/fimmu.2025.1699079 (PMC12714941; doi:10.3389/fimmu.2025.1699079)
Supplement: Supplementary file 21 [file Table6.docx]

CYP2E1

ESRRA

IDI2

CYP11B1

ACACB

ARSG

GRHL1

SLC44A5

PITPNM2

PIP4K2B

MOGAT1

STARD6

CDK8

THEM4

DGKH

PLB1

MFSD2A

DGKB

THRSP

PPARGC1B

SP1

CBR4

PIK3C2A

PIKFYVE

ACER1

CEL

CYP39A1

ARSB

SPTLC1

HSD17B12

ZNF638

CERS3

CYP19A1

NR1H4

HILPDA

SAR1B

MBTPS2

DGKE

OSBPL1A

PLA2G4C

CYP2U1

HMGCLL1

PRKD3

ESYT3

ACOT11

ACAT1

PTGS2

SYNJ2

LPGAT1

SLC44A4

B4GALNT1

ALOX15B

ASAH1

CYP4B1

GDPD1

AGK

TRIB3

ESYT2

AKR1C1

HEXA

ACSF3

PLD1

SQLE

PCCB

FAR2

FAR1

PRKAB2

UBE2I

KDSR

PPARA

PIK3R6

SREBF1

NFYC

CHPT1

AWAT1

PLEKHA8

TXNRD1

CEPT1

CCND3

CYP17A1

GPD1

OSBP

HSD17B4

INPP4B

ALB

ALDH3B1

FABP6

PLD4

ELOVL5

PIK3R2

NCOA6

ACBD5

INPP5D

CCNC

ARSK

PPARGC1A

PIK3C3

ABCA1

TBL1X

BDH1

ABCB4

ARF3

STARD7

PTGES3

CTSA

ACADVL

PPP1CC

RAN

SCD

PPP1CA

NCOR1

DHCR24

PSAP

RAB14

PPT1

HADHB

HADH

PLD3

GPX4

ACLY

ECHS1

LGMN

SREBF2

CDIPT

AKR1B1

FDPS

SCP2

CSNK2B

PPP1CB

ALDH2

PTDSS1

MAPKAPK2

INPPL1

ALDH9A1

MBTPS1

INSIG1

ACSL3

RELA

DHCR7

LBR

LPCAT1

PON2

AKR1A1

HEXB

GNPAT

ACSL1

MED13

ACAA2

ACAA1

ALDH3A2

NDUFAB1

TSPO

CPNE3

PLIN3

CREBBP

MTMR3

FADS2

EP300

LSS

CDK4

LYPLA2

GGPS1

SEC24C

ACADS

SEC24D

RXRA

DBI

CYP1B1

DECR1

LPIN2

ACADM

PITPNB

HMGCR

VAPB

CSNK1G2

GDE1

MED14

EBP

PRKACB

PIK3R3

HMGCL

OXCT1

INPP5K

LPCAT3

SACM1L

SEC24B

PRKACA

AHR

GPX2

AGT

FHL2

ARNT2

STARD3

LYPLA1

MVD

CAV1

TGFB1

SPTLC2

MTMR2

PHYH

DGKA

OCRL

MED1

GCDH

MED12

PMVK

LPL

ADIRF

CSNK2A2

INPP5F

CPT1A

FDX1

PLA2G2A

FIG4

SLC25A20

DGAT1

PNPLA6

STS

PITPNM1

PCCA

PIK3CD

HPGD

NR1H3

MORC2

CEBPD

CYP27A1

FABP4

CEBPA

PTEN

MVK

ME1

SMARCD3

MTM1

NFYA

HSD11B2

GSTM4

ALOX5AP

PTPN13

PCYT1A

ACOT8

GLIPR1

FAAH

CHKA

ACOX3

VDR

ELOVL6

CPT2

CYP11A1

SEC23A

MED7

PIK3CA

GALC

LCAT

ARSA

ALOX5

APOA1

PLA2G15

PIK3C2B

HSD3B1

STAR

INPP4A

ACOT13

CROT

HMGCS2

IDI1

CGA

NRF1

SRD5A1

PLA2G6

INPP5E

HSD17B2

MTMR9

UGCG

ALDH3B2

GC

CLOCK

FABP7

CYP2J2

SLC22A5

PLEKHA6

PTGS1

ABCD1

PEX11A

EHHADH

EGR2

MTF1

ACADSB

ACOX2

ACHE

AGPS

HSD11B1

BCHE

NPAS2

PIP4K2A

GGT5

SMPD2

PIP5K1B

ALAS1

CYP27B1

CDS1

LRP2

POMC

NCOA2

FABP3

CYP1A1

SLC27A2

HMGCS1

HSD17B1

CRAT

PRKD1

FABP1

PNLIP

PLIN1

ACSM3

DPEP1

CYP3A4

ANKRD1

ACADL

CSNK2A1

RAB5A

PI4KB

CYP4F11

PLA2G5

WNT10B

ADH1C

SULT2A1

HSD3B2

PLA2G1B

SERPINA6

LIPF

PON1

SLCO1B3

PIK3CG

DGKG

BMX

ACSBG1

LTC4S

CD36

CYP24A1

GLB1L

MED22

SLC2A4

LIPC

PNLIPRP1

HPGDS

PCYT1B

CUBN

DGKI

ARSD

BAAT

CPNE1

CH25H

SRD5A2

NEU3

MED20

HSD17B3

MED6

LEP

SLC10A2

AKR1D1

TNF

ALPI

ADIPOQ

SLC10A1

ALOX12

PRKACG

SLCO1A2

ALOX15

ALOX12B

CYP7B1

GK

PTGES

PIP5K1A

CYP7A1

PLA2R1

DEGS1

FABP2

MOGAT2

ADH6

DGKZ

DGKQ

SYNJ1

CYP1A2

PEMT

ACOX1

NCOA3

ALOXE3

PRKAA2

NCOR2

CYP2R1

FDXR

ADH1A

ACOT7

PPARD

DGKD

MED25

TBXAS1

PTGIS

CYP2C8

ABCC3

OSBPL7

LIPE

ABCB11

TNFAIP8

UGT8

SGPL1

PCK1

PPARG

WNT1

HADHA

FDFT1

VAPA

ADH5

ESYT1

NEU1

ALDH7A1

KPNB1

NCOA1

NR2F2

PLIN2

MSMO1

RXRB

AKR1C3

MBOAT7

KLF5

CBR1

OSBPL2

NFKB1

NSDHL

PRKD2

PI4K2A

GPS2

SIN3B

EPHX2

SMPD1

INSIG2

RGL1

PCYT2

ACAT2

ADH1B

OSBPL3

PLD2

ALDH1B1

GM2A

PNPLA4

ECI1

ARNTL

SPHK2

GPD2

SLC25A1

TM7SF2

PLA2G4A

SLCO1B1

CPNE6

RORA

CYP4F2

ADH7

AKR1C4

CYP4F8

AGPAT2

ARNT

MGLL

ACSL6

B3GALNT1

SC5D

PTGDS

CDK19

SLC25A17

PNLIPRP2

ACACA

MED13L

FASN

PIK3R1

LPIN1

MTMR4

SCAP

SBF1

CERS6

SPTSSA

CEBPB

GPD1L

PIP5K1C

OSBPL8

CHD9

PIK3CB

DDHD2

PNPLA2

PIK3R4

CDS2

PIAS4

SEC24A

SGMS1

ABHD3

LPCAT4

MED8

MCAT

MBOAT2

GDPD5

MTMR1

G0S2

HSD17B8

ZNF467

INPP5J

PON3

ELOVL2

ABHD5

STARD5

CYP2C9

MTMR6

GLA

ARSF

TNFRSF21

CYP11B2

PIK3C2G

PRKAG2

MED24

MED27

GK2

AGPAT1

MTMR7

NUDT7

CYP2C19

HDAC3

VAC14

OSBPL10

PLEKHA2

MED4

THRAP3

HSD17B11

ECI2

OSBPL9

PTGES2

AGPAT5

NFYB

NR1H2

RUFY1

MID1IP1

BDH2

ACSL5

MED9

CERK

AACS

MED28

PLBD1

ORMDL2

ABHD4

MECR

PCTP

ACP6

SRD5A3

ACSF2

MED23

MLYCD

CERS4

PIP4K2C

PLEKHA4

ETNK1

PLEKHA1

HSD17B14

LIPG

TGS1

SPHK1

GLTP

ETNK2

MED31

PGS1

CIDEC

ACBD4

FA2H

DPEP2

APOA2

ELOVL4

PLA1A

ACSS3

DECR2

AGPAT4

SMPD3

CPNE7

GDPD3

AGPAT3

SLC27A5

TIAM2

ARSJ

OLAH

ACAD10

DPEP3

TPTE

SCD5

KLF4

MTMR10

CYP46A1

PLA2G2D

SPTLC3

PIK3R5

ADPRM

PNPLA3

DHRS7B

CPTP

PLA2G3

HAO2

PLEKHA5

MTMR12

PTDSS2

ETNPPL

ANGPTL4

PLA2G12A

PECR

CHAT

PITPNM3

SGPP1

CIDEA

NEU2

PLA2G2E

PLA2G2F

MED16

TBL1XR1

MED17

ACOT9

ACSBG2

LDLRAP1

MTMR14

MED15

CERS2

SLC27A3

SLC44A1

ACER3

HSD3B7

LPCAT2

NEU4

STARD3NL

STARD10

ORMDL1

HACL1

ARV1

MED10

ORMDL3

PNPLA8

PLEKHA3

OSBPL5

TNFAIP8L2

ADH4

OSBPL6

GBA2

CRLS1

APOA5

DGAT2

PLA2G12B

SLC44A2

GPCPD1

CERS5

SUMF2

SIN3A

ACBD6

GPAM

SPNS2

MED29

PTPMT1

SAMD8

DDHD1

SBF2

MCEE

MED19

STARD4

SLC27A1

FAM120B

FITM2

SUMF1

MED11

LCLAT1

SGMS2

ELOVL7

MBOAT1

TNFAIP8L1

CPT1C

MED26

EBF1

MED30

PPM1L

SLC44A3

HELZ2

PNPLA7

PTGR1

SLC51A

AHRR

ACOT4

FITM1

TMEM86B

ENPP6

ARSI

PTGR2

FAAH2

SLC51B

ARF1

CYP8B1

PNPLA5

ELOVL3

MOGAT3

NUDT19

LIPH

PLA2G4F

PHOSPHO1

MMAA

ACOT12

ENPP7

GLYCTK

SGPP2

SPTSSB

TECRL

LIPI

PLD6

CYP4F22

ATP1A1

ATP1A2

ATP1A3

ATP1A4

ATP1B1

ATP1B2

ATP1B3

ATP1B4

FXYD2

FXYD4

IGF1

INS

INSR

IRS1

IRS2

IRS4

KCNJ1

KRAS

MAPK1

MAPK3

NEDD4L

NR3C2

PDPK1

PRKCA

PRKCB

PRKCG

SCNN1A

SCNN1B

SCNN1G

SFN

SGK1

SLC9A3R2

ALDH6A1

IMPA1

IMPA2

INPP1

INPP5A

INPP5B

IPMK

IPPK

ISYNA1

ITPK1

ITPKA

ITPKB

MINPP1

MIOX

PI4KA

PLCB1

PLCB2

PLCB3

PLCB4

PLCD1

PLCD3

PLCD4

PLCE1

PLCG1

PLCG2

PLCZ1

TPI1

AKR1B10

CYP2C18

CYP3A43

CYP3A5

CYP3A7

JMJD7-PLA2G4B

PLA2G10

PLA2G2C

PLA2G4B

PLA2G4E

ABAT

ACSS1

ACSS2

HIBCH

LDHA

LDHAL6A

LDHAL6B

LDHB

LDHC

MMUT

SUCLA2

SUCLG1

SUCLG2

SUCLG2P2

ACSL4

APOC3

AQP7

CPT1B

CYP4A11

CYP4A22

FABP5

ILK

MMP1

OLR1

PCK2

PLTP

RXRG

SLC27A4

SLC27A6

SORBS1

UBC

UCP1

UGDH

UGT1A1

UGT1A10

UGT1A3

UGT1A4

UGT1A5

UGT1A6

UGT1A7

UGT1A8

UGT1A9

UGT2A1

UGT2A3

UGT2B10

UGT2B11

UGT2B15

UGT2B17

UGT2B28

UGT2B4

UGT2B7

CBR3

CYP2B6

CYP4F3

GGT1

GGT6

GGT7

GPX1

GPX3

GPX5

GPX6

GPX7

LTA4H

AKT1

AKT2

AKT3

BTK

CSF2

FCER1A

FCER1G

FYN

GAB2

GRB2

HRAS

IL13

IL3

IL4

IL5

LAT

LCP2

LYN

MAP2K1

MAP2K2

MAP2K3

MAP2K4

MAP2K6

MAP2K7

MAPK10

MAPK11

MAPK12

MAPK13

MAPK14

MAPK8

MAPK9

MS4A2

NRAS

PRKCD

PRKCE

RAC1

RAC2

RAC3

RAF1

SOS1

SOS2

SYK

VAV1

VAV2

VAV3

AMDHD2

CHIA

CHIT1

CMAS

CYB5R1

CYB5R3

FCSK

FPGT

GALE

GALK1

GALK2

GALT

GCK

GFPT1

GFPT2

GFUS

GMDS

GMPPA

GMPPB

GNE

GNPDA1

GNPDA2

GNPNAT1

GPI

HK1

HK2

HK3

MPI

NAGK

NANP

NANS

NPL

PGM1

PGM2

PGM3

PMM1

PMM2

RENBP

UAP1

UGP2

UXS1

CHKB

GPAT2

GPAT3

GPAT4

PISD

PLPP1

PLPP2

PLPP3

TAZ

ACYP1

ACYP2

DLAT

DLD

GLO1

GRHPR

HAGH

HAGHL

LDHD

MDH1

MDH2

ME2

ME3

PC

PDHA1

PDHA2

PDHB

PKLR

PKM

DPM2

GPAA1

GPLD1

PGAP1

PIGA

PIGB

PIGC

PIGF

PIGG

PIGH

PIGK

PIGL

PIGM

PIGN

PIGO

PIGP

PIGQ

PIGS

PIGT

PIGU

PIGV

PIGW

PIGX

PIGY

PIGZ

ACO1

ACO2

AFMID

CS

HAO1

HYI

MTHFD1

MTHFD1L

MTHFD2

MTHFD2L

PGP

AOX1

BST1

CD38

ENPP1

ENPP3

NADK

NADSYN1

NAMPT

NMNAT1

NMNAT2

NMNAT3

NMRK1

NNMT

NNT

NT5C

NT5C1A

NT5C1B

NT5C2

NT5C3A

NT5E

NT5M

NUDT12

PNP

QPRT

AWAT2

TKFC

ENPP2

PAFAH1B1

PAFAH1B2

PAFAH1B3

PAFAH2

PLA2G7

ACSM1

ACSM2A

ACSM4

ACSM5

ALDH5A1

GAD1

GAD2

L2HGDH

OXCT2

ACP1

ACP2

ACP3

ACP4

ACP5

FLAD1

PHPT1

RFK

TYR

BPNT1

CHST11

CHST12

CHST13

PAPSS1

PAPSS2

SULT1A1

SULT1A2

SULT1A3

SULT1A4

SULT1E1

SULT2B1

SUOX

ADO

CDO1

CSAD

ACER2

ASAH2

B4GALT6

DEGS2

GAL3ST1

GBA

GLB1

SMPD4

ALDH1A1

ALDH1A2

BCO1

CYP26A1

CYP26B1

CYP26C1

CYP2A13

CYP2A6

CYP2A7

DHRS3

DHRS4

DHRS4L2

DHRS9

LRAT

RDH10

RDH11

RDH12

RDH16

RDH5

RDH8

RETSAT

RPE65

ABCD2

ABCD3

ABCD4

AGXT

AMACR

CAT

DAO

DDO

ECH1

GSTK1

IDH1

IDH2

MPV17

MPV17L

NOS2

PAOX

PEX1

PEX10

PEX11B

PEX11G

PEX12

PEX13

PEX14

PEX16

PEX19

PEX2

PEX26

PEX3

PEX5

PEX6

PEX7

PIPOX

PRDX1

PRDX5

PXMP2

PXMP4

SOD1

SOD2

XDH

AOC2

AOC3

CNDP1

DPYD

DPYS

SMS

SRM

UPB1

ALDH1A3

ALDH3A1

CYP2D6

FMO1

FMO2

FMO3

FMO4

FMO5

GSTA1

GSTA2

GSTA3

GSTA4

GSTA5

GSTM1

GSTM2

GSTM3

GSTM5

GSTO1

GSTO2

GSTP1

GSTT1

GSTT2

GSTZ1

MAOA

MAOB

MGST1

MGST2

MGST3

APOE

CETP

LDLR

APOB

ABCG5

ABCA3

ABCG8

MTR

MMACHC

FADS1

SLC19A3

MTTP

SFTPB

LINC01672

SFTPC

PCSK9

MTRR

IL6

TPK1

MMADHC

CRP

NPC1

MTHFR

LIPA

ACE

CSF2RA

ANGPTL3

OPA3

CBS

BSCL2

APOC2

MMAB

GHRL

GPT

SCARB1

LEPR

ETFDH

HNF4A

SLC25A19

GBA1

OTC

H19

SOD2-OT1

RETN

SIRT1

LPA

CES1

LMNA

BDNF-AS

GCG

SERPINE1

MIR122

MIR33A

SMAD5-AS1

TPMT

G6PD

TLR4

APOA4

NPC2

TAFAZZIN

SLC17A5

G6PC1

MLXIPL

TP53

ABCG1

BGLAP

MT-TP

IL1B

ESR1

LDAH

SHBG

CHKB-CPT1B

AUP1

COG2

CP

MIR7-3HG

ADRB3

CCL2

NOS3

APP

CNR1

SECISBP2

UCP2

LMBRD1

NPC1L1

SLC2A1

TMX2-CTNND1

ABCB1

ETFA

PRKAA1

PAH

IL10

FGF21

C3

COMMD1

MEG3

RBP4

STARD13

SLC25A13

MIR21

MTOR

NR1I2

DPP4

UCP3

TCF7L2

REN

HIF1A

HFE

SNCA

ERLIN2

CYP21A2

HCFC1

PRODH

SOAT2

ADRB2

SEC14L2

MT-ATP6

ETFB

GSR

F2

CFTR

SOAT1

ADSL

MIR33B

FGF23

MIR17

MIR126

TF

NAT2

ADA

MT-TL1

STARD8

HSD17B10

HNF1A

ERLIN1

SLC25A4

POR

BTD

PRKAG3

SERPINA3

ADIPOR2

MIR34A

TRU-TCA1-1

STARD9

APOC1

ASS1

MCOLN1

MT-CYB

LDAF1

PLIN5

CSF2RB

GCKR

NR1I3

EPHX1

CASR

CERNA3

FTO

COMT

PRKAB1

MIR155

QDPR

PTH

GALNT2

HMOX1

ACAD9

NR3C1

SCO2

EMSLR

CPS1

DIO1

BLTP1

SELE

ABCC8

TM6SF2

SI

MAT1A

FH

MB

SURF1

NQO1

GAA

ANGPTL8

BLTP3A

ACAD8

DHFR

PRL

LRP1

ASL

IVD

EDN1

TRMT10A

BCS1L

SLC19A1

MPO

PTPN1

HPRT1

OGDH

SLC37A4

LINC02605

NFE2L2

ICAM1

SLC16A1

BLTP3B

VEGFA

ATP7B

SEC14L3

ANO10

NAGLU

FBP1

VCAM1

AMPD1

AMPD3

POLG

SFTA3

BCKDHB

KCNJ11

SLC2A2

VKORC1

MC4R

ALPL

UNC119B

GSS

TFR2

CERT1

ABCC2

MIR27B

SDHA

APOH

TANGO2

ABCA4

FAH

MIR22

RFTN1

USH2A

AHCY

MIF4GD-DT

FAM3A

MIR140

HBB

IDUA

KL

SEC14L1

GAMT

ALDOB

NDUFS4

HSD17B13

PPIG

TTR

SERPINA1

MIR148A

SDHB

RYR1

ARG1

ILRUN

CYCS

GHR

MALAT1

SIRT6

ABCA12

GAPDH

ETHE1

PNPO

USF1

GH1

HAMP

GPIHBP1

H2AC18

TTPA

MIR125A

DDC

NPY

AGL

CCR6

DPAGT1

COX5A

TRA-TGC7-1

PDHX

NPPA

HSPG2

ATP5F1A

HJV

SEC14L4

TYMS

NR1D1

HPD

APBB1

PYGM

TFRC

APRT

PYY

SEC14L5

HLCS

PDK4

SLC5A2

PNPLA1

LRPPRC

MCCC1

SLC12A3

ADK

GNAS

PTS

CYP4V2

GLUL

CTH

ABCG2

BLTP2

GAS5

CD4

FABP12

KHK

ALG1

IGF2R

SEC14L6

MGAM

AR

FADS3

CD1A

CALCA

BMP6

TRIB1

MIR146B

NAGS

HP

IGFBP1

HLA-DRB1

IGFBP3

MCCC2

BHMT

GOT2

ACTB

GUSB

DOCK7

GLUD1

LEPQTL1

CLN3

PGR-AS1

LINC-ROR

ALDH4A1

FAM3B

TNFSF11

ECM1

VCP

TAT

MIR223

GLDC

ADIPOR1

MIR27A

SIRT3

DMGDH

CXCL8

FBN1

UGT1A

APOC4

BCKDHA

MIR483

SPP1

MIR144

SLC7A7

SCARB2

PDE5A

ST3GAL4

DDOST

XIST

FAS

LPXN

ALDH18A1

PDP1

MT-ND1

CA5A

MT-ATP8

DYRK1B

BDNF

MIR29A

RARRES2

VDAC1

ABCA2

TNFRSF11B

FAM3D

MYC

EPO

ALG8

PRKN

KCNQ1

SCARNA5

RRM2B

ELN

MT-CO1

H6PD

IAPP

DNM1L

PPP1R15B

COQ8A

ALG6

MIR199A1

APOM

FAM3C

FECH

MIR143

TCN2

STK11

PFKM

IGF2

SMARCA4

HYCC1

CLCNKB

FOXO1

ATP8B1

NDUFS2

COASY

CFH

PHKA2

ALPP

SLC34A1

OPA1

ANXA5

ABCC1

TLR2

TYMP

GALNS

CD1B

IFNG

BMAL1

MOCS2

CTSD

ZPR1

CIDEB

B2M

MIR23A

BCL2

GNPTAB

FOXC2

CYB5A

SLC40A1

ESR2

AOMS1

AHSG

MIR132

GATM

MIR30A

ALG2

NR1D2

THAP11

ATP7A

PANK2

VLDLR

HLA-B

CHROMR

NDUFB11

RAB7A

FOXO3

PVT1

SLC52A2

LCT

PHKB

SLC52A1

MIR145

SLC5A6

CACNA1S

CLPS

ATG2A

ODC1

EGFR

IL18

ATM

PEPD

GYS1

SLC12A1

MIR130A

EPRS1

STAT3

PRKAR1A

GNB3

FGFR4

SNORD15A

TMPRSS6

AGTR1

MIRLET7C

VPS13A

MT-RNR1

APOC4-APOC2

GALM

MIR192

DLEU2

FGFR1

GSK3B

PHKG2

OAT

DLG4

FTCD

SLC4A1

MSR1

TUG1

ATAD3A

MT-TE

SHMT2

GLP1R

COQ9

CYP4F12

MIR9-1

MIRLET7D

SHMT1

SLC11A2

APOD

PTPN11

CST3

BRAF

PGR

UMPS

FTH1

GLS

NR5A1

NDUFS1

UQCRFS1

SLC39A8

CELA2A

ERCC2

RPIA

MFN2

CD1D

TRC-GCA24-1

BLOC1S1

DBT

COX10

COSMOC

CUL3

MIA2

CAD

CAVIN1

AIFM1

MBP

GCLC

AASS

DNAH8

BRCA1

ABCA7

CD1C

SLC46A1

ZMPSTE24

AKR1C2

TOMM40

SERPINC1

GBE1

PHEX

PLIN4

NR0B2

SDHD

TH

MIR342

TPO

HSP90AA1

TTN

ALDOA

NAT1

DSP

BCAT2

MIR29C

STX1A

MYBPC3

MMP9

VWF

MIR142

KRT14

FTL

TRE-TTC3-1

SLC25A10

PDE3B

PRNP

DBH

SLC6A8

LBP

ALG13

SLC5A1

HTT

DYNC2LI1

CLU

HOGA1

ARG2

DNMT1

BSND

ATG7

FFAR4

COX4I1

GCH1

ISCU

CFHR1

SLC25A12

CYP2S1

PLAT

PARP1

APOL1

ERCC6

XK

COQ2

BMP1

CD44

MT-TK

VPS13C

CAV2

LRP5

MT-ND3

SLC25A15
