# Supplementary material for: Integration of single-cell and bulk RNA-seq via machine learning to reveal ferroptosis- and lipid metabolism-driven immune landscape heterogeneity and predict immunotherapy response in colon cancer
Source: Front Immunol. 2025 Dec 5;16:1699079. doi: 10.3389/fimmu.2025.1699079 (PMC12714941; doi:10.3389/fimmu.2025.1699079)
Supplement: Supplementary file 22 [file Table7.docx]

GPX3,PDK4,DPEP1,AHCY,EMSLR,MEG3,TFR2,ACSL6,CAV1,PKM,ARNTL,SNCA,CD36,PRKCB,SQLE,PVT1,CERS6,TP53,CDO1,VDAC1,SCD,PRKAA2,TF,RARRES2,PGR-AS1,AKR1C1,NQO1,AR,MYC,APOC1,TYMS,AKR1C2,SCARNA5,TFRC,FABP4,PRNP,CYP4F8,PCSK9,ESR1,IL1B,CP,ALOXE3,CYP24A1,ANKRD1,TTPA,SLC25A10,ALB,NR1H4,MIOX,NOS2,MIR7-3HG,H19,KRT14,IFNG,ADIPOQ,SFTA3
