# Supplementary material for: Integration of single-cell and bulk RNA-seq via machine learning to reveal ferroptosis- and lipid metabolism-driven immune landscape heterogeneity and predict immunotherapy response in colon cancer
Source: Front Immunol. 2025 Dec 5;16:1699079. doi: 10.3389/fimmu.2025.1699079 (PMC12714941; doi:10.3389/fimmu.2025.1699079)
Supplement: Supplementary file 23 [file Table8.docx]

**WP_FERROPTOSIS：**ACSL1, ACSL3, ACSL4, ACSL5, ACSL6, AIFM2, AKR1C1, AKR1C2, AKR1C3, ALOX15, ATG5, ATG7, BACH1, CBS, CHMP5, CHMP6, CISD1, COQ2, CP, CTH, CYBB, DPP4, FDFT1, FDFT1, FTH1, FTL, FTMT, GCH1, GCLC, GCLM, GPX4, GSS, HMGCR, HMOX1, HSPB1, IREB2, LPCAT3, MAP1LC3A, MAP1LC3B, MAP1LC3C, MIR4651, NCOA4, NOX1, NOX4, PCBP1, PCBP2, PHKG2, POR, PRNP, SAT1, SAT2, SLC11A2, SLC1A5, SLC38A1, SLC39A14, SLC39A8, SLC3A2, SLC40A1, SLC7A11, STEAP3, TF, TFRC, TP53, TXNRD1, VDAC2, VDAC3

**GOBP_LIPID_METABOLIC_PROCESS：**A3GALT2, A4GALT, AACS, AADAC, AASDH, ABCA1, ABCA12, ABCA2, ABCA3, ABCA4, ABCA5, ABCA7, ABCA8, ABCB11, ABCB11, ABCB4, ABCD1, ABCD2, ABCD3, ABCD4, ABCG1, ABCG4, ABHD1, ABHD12, ABHD12B, ABHD15, ABHD16A, ABHD16A, ABHD16A, ABHD16A, ABHD16A, ABHD16A, ABHD16A, ABHD16B, ABHD2, ABHD3, ABHD4, ABHD5, ABHD6, ABHD8, ABO, ABO, ABO, ACAA1, ACAA2, ACACA, ACACA, ACACB, ACAD10, ACAD11, ACAD8, ACAD9, ACADL, ACADM, ACADS, ACADSB, ACADVL, ACAT1, ACAT2, ACBD3, ACBD4, ACBD5, ACBD7, ACER1, ACER2, ACER3, ACER3, ACLY, ACOT1, ACOT11, ACOT12, ACOT13, ACOT2, ACOT4, ACOT6, ACOT7, ACOT8, ACOX1, ACOX2, ACOX3, ACOXL, ACP3, ACP6, ACSBG1, ACSBG2, ACSF2, ACSF3, ACSL1, ACSL3, ACSL4, ACSL5, ACSL6, ACSM1, ACSM2A, ACSM2B, ACSM3, ACSM4, ACSM5, ACSM6, ACSS1, ACSS2, ACSS3, ADGRF5, ADH1A, ADH1B, ADH1C, ADH4, ADH5, ADH6, ADH7, ADHFE1, ADIPOQ, ADIPOR1, ADIPOR2, ADIPOR2, ADM, ADORA1, ADRA2A, ADTRP, AFP, AGAP2, AGK, AGK, AGMO, AGPAT1, AGPAT1, AGPAT1, AGPAT1, AGPAT1, AGPAT1, AGPAT1, AGPAT2, AGPAT3, AGPAT4, AGPAT5, AGPAT5, AGPS, AGT, AGTR1, AIG1, AJUBA, AKR1A1, AKR1B1, AKR1B10, AKR1B15, AKR1C1, AKR1C2, AKR1C3, AKR1C4, AKR1D1, AKR7A2, AKT1, AKT2, ALDH1A1, ALDH1A2, ALDH1A3, ALDH1L2, ALDH3A1, ALDH3A2, ALDH3B1, ALDH3B2, ALDH8A1, ALG1, ALG10, ALG10B, ALG11, ALG12, ALG14, ALG2, ALG3, ALG6, ALG8, ALG9, ALK, ALKBH7, ALOX12, ALOX12B, ALOX15, ALOX15B, ALOX5, ALOX5, ALOX5AP, ALOXE3, AMACR, AMBRA1, ANG, ANGPTL3, ANGPTL4, ANGPTL8, ANO9, ANXA1, AOAH, AOX1, APOA1, APOA2, APOA4, APOA5, APOB, APOBEC1, APOBR, APOC1, APOC2, APOC3, APOC4, APOD, APOE, APOF, APOH, APOL1, APOL2, APOL4, APOL5, APOL5, APP, APPL2, ARMC5, ARSA, ARV1, ASAH1, ASAH2, ASAH2B, ASMT, ASPG, ASXL3, ATF2, ATG14, ATM, ATP1A1, ATP5F1A, ATP5F1B, ATP6V1B1, ATP8B1, AUH, AVIL, AVP, AVPR1A, AWAT1, AWAT2, B3GALNT1, B3GALT1, B3GALT2, B3GALT4, B3GALT4, B3GALT4, B3GALT4, B3GALT4, B3GALT5, B3GNT5, B4GALNT1, B4GALNT2, B4GALT1, B4GALT3, B4GALT4, B4GALT5, B4GALT6, BAAT, BAAT, BAX, BBS4, BCAT1, BCAT2, BCKDHB, BCL11B, BCO1, BCO2, BDH1, BDH1, BDH2, BECN1, BLOC1S6, BMP2, BMP5, BMP6, BMX, BPNT1, BPNT2, BRCA1, BSCL2, BTN2A1, C1QTNF2, C3, CACNA1H, CAPN2, CAT, CAV1, CAV3, CBR1, CBR4, CCDC3, CCDC71L, CCL19, CCL21, CCN1, CCR7, CD19, CD36, CD74, CD81, CDC42, CDIPT, CDK4, CDS1, CDS2, CEACAM1, CEBPA, CEL, CEPT1, CERK, CERKL, CERS1, CERS2, CERS3, CERS4, CERS5, CERS6, CERT1, CES1, CES1, CES2, CETP, CFTR, CGA, CH25H, CHAT, CHKA, CHKB, CHP1, CHPT1, CHRM5, CHST10, CIDEA, CISH, CLCN2, CLN3, CLN6, CLN8, CLN8, CLPS, CLPSL1, CLPSL2, CLU, CNEP1R1, CNR1, COMT, COQ2, COX10, CPNE1, CPNE7, CPS1, CPT1A, CPT1B, CPT1C, CPT2, CPTP, CRABP1, CRABP2, CRAT, CREB1, CREBL2, CRH, CRK, CRKL, CRLS1, CROT, CRPPA, CRTC3, CRYL1, CSF1R, CSNK1G2, CTDNEP1, CTDNEP1, CTH, CUBN, CWH43, CYB5R1, CYB5R2, CYB5R3, CYGB, CYP11A1, CYP11A1, CYP11B1, CYP11B2, CYP17A1, CYP19A1, CYP1A1, CYP1A2, CYP1B1, CYP21A2, CYP21A2, CYP21A2, CYP21A2, CYP21A2, CYP21A2, CYP24A1, CYP26A1, CYP26B1, CYP26C1, CYP27A1, CYP27B1, CYP27C1, CYP2A13, CYP2A6, CYP2A7, CYP2B6, CYP2B6, CYP2C18, CYP2C19, CYP2C8, CYP2C9, CYP2D6, CYP2D6, CYP2D6, CYP2D6, CYP2D6, CYP2D6, CYP2D7, CYP2D7, CYP2D7, CYP2D7, CYP2E1, CYP2F1, CYP2G1P, CYP2J2, CYP2R1, CYP2S1, CYP2U1, CYP2W1, CYP39A1, CYP3A4, CYP3A43, CYP3A5, CYP3A7, CYP46A1, CYP4A11, CYP4A22, CYP4B1, CYP4F11, CYP4F12, CYP4F2, CYP4F22, CYP4F3, CYP4F8, CYP4V2, CYP4X1, CYP4Z1, CYP51A1, CYP7A1, CYP7B1, CYP8B1, DAB2, DAB2IP, DAGLA, DAGLB, DBI, DDHD1, DDHD2, DDX20, DECR1, DECR2, DECR2, DEGS1, DEGS2, DGAT1, DGAT1, DGAT1, DGAT2, DGAT2L6, DGKA, DGKB, DGKD, DGKD, DGKE, DGKG, DGKH, DGKI, DGKK, DGKQ, DGKZ, DHCR24, DHCR7, DHDDS, DHH, DHRS11, DHRS11, DHRS13, DHRS2, DHRS3, DHRS4, DHRS4, DHRS4L1, DHRS4L1, DHRS4L1, DHRS4L1, DHRS4L2, DHRS7, DHRS7B, DHRS9, DIO2, DISP3, DKK3, DKKL1, DNAJC15, DNAJC19, DOLK, DOLPP1, DPAGT1, DPEP1, DPEP2, DPM1, DPM2, DPM3, EBP, EBPL, ECH1, ECH1, ECHDC1, ECHDC2, ECHDC3, ECHS1, ECI1, ECI2, EDF1, EDN1, EDN2, EDNRB, EEF1A2, EFR3A, EFR3B, EGR1, EHHADH, EIF6, ELOVL1, ELOVL2, ELOVL3, ELOVL4, ELOVL5, ELOVL6, ELOVL7, ENPP2, ENPP6, ENPP7, ENSG00000205794, EPHA8, EPHX1, EPHX2, EPHX3, ERFE, ERG28, ERLIN1, ERLIN2, ESR1, ETFA, ETFB, ETFBKMT, ETFDH, ETNK1, ETNK2, F2, FA2H, FAAH, FAAH2, FABP1, FABP2, FABP3, FABP5, FABP6, FADS1, FADS2, FADS2B, FADS3, FADS6, FAH, FAM126A, FAM126B, FAM135A, FAM135B, FAM3A, FAR1, FAR2, FASN, FAXDC2, FBXW7, FDFT1, FDFT1, FDPS, FDX1, FDXR, FGF1, FGF19, FGF2, FGF21, FGF23, FGFR3, FGFR4, FGR, FIG4, FITM1, FITM1, FITM2, FLT1, FLT3, FMC1, FMO5, FNTB, FPR2, FSHB, FUCA1, FUT1, FUT2, FUT3, FUT5, FUT6, FUT7, FUT9, G6PC1, G6PD, GAL, GAL3ST1, GAL3ST2, GAL3ST2, GAL3ST3, GAL3ST4, GALC, GALR2, GATA6, GBA, GBA, GBA2, GBA3, GBGT1, GC, GCDH, GDE1, GDPD1, GDPD2, GDPD3, GDPD4, GDPD5, GFI1, GGPS1, GGT1, GGT5, GHSR, GIP, GK, GK2, GK5, GLA, GLB1, GLT6D1, GLTP, GLYATL2, GLYATL3, GM2A, GNB3, GNPAT, GOLM1, GPAA1, GPAM, GPAT2, GPAT3, GPAT4, GPCPD1, GPD1, GPER1, GPIHBP1, GPLD1, GPR31, GPS2, GPS2, GPX1, GPX4, GPX5, GPX5, GSTA1, GSTA3, GSTM1, GSTM2, GSTM4, GSTP1, H6PD, HACD1, HACD2, HACD3, HACD4, HACL1, HADH, HADHA, HADHB, HAO1, HAO2, HCAR1, HCAR2, HDHD5, HDLBP, HEXA, HEXB, HINT2, HMGCL, HMGCLL1, HMGCR, HMGCS1, HMGCS2, HNF4A, HPGD, HPGDS, HSD11B1, HSD11B2, HSD17B1, HSD17B10, HSD17B11, HSD17B12, HSD17B13, HSD17B14, HSD17B2, HSD17B3, HSD17B4, HSD17B6, HSD17B7, HSD17B8, HSD17B8, HSD17B8, HSD17B8, HSD17B8, HSD17B8, HSD3B1, HSD3B2, HSD3B7, HSPG2, HTD2, HTR2A, HTR2B, HTR2C, HTRA2, HULC, IAH1, ID2, IDH1, IDI1, IDI2, IFNG, IGF1R, IGFBP7, IL1A, IL1B, IL1RN, IL4, ILVBL, IMPA1, IMPA2, INPP1, INPP4A, INPP4B, INPP5A, INPP5B, INPP5D, INPP5D, INPP5E, INPP5F, INPP5J, INPP5K, INPPL1, INS, INSIG1, INSIG2, IP6K1, IP6K2, IP6K3, IPMK, IRS1, IRS2, ISYNA1, ITGB8, ITPKA, ITPKB, ITPKC, IVD, JAZF1, KBTBD2, KDSR, KIT, LACTB, LAMTOR1, LARGE1, LBR, LCAT, LCLAT1, LDAH, LDLR, LDLRAP1, LEP, LEPR, LGALS12, LGALS13, LGMN, LHB, LIAS, LIMA1, LIPA, LIPC, LIPE, LIPF, LIPG, LIPH, LIPI, LIPJ, LIPK, LIPM, LIPN, LIPT1, LMF1, LONP2, LPA, LPCAT1, LPCAT1, LPCAT2, LPCAT3, LPCAT4, LPGAT1, LPIN1, LPIN2, LPIN3, LPL, LRAT, LRCOL1, LRP1, LRP10, LRP2, LRP5, LRP8, LSS, LSS, LTA4H, LTC4S, LTC4S, LYN, LYPLA1, LYPLA2, MACROH2A1, MALRD1, MAPK14, MBLAC2, MBOAT1, MBOAT2, MBOAT4, MBOAT7, MBOAT7, MBOAT7, MBOAT7, MBOAT7, MBOAT7, MBOAT7, MBOAT7, MBOAT7, MBOAT7, MBTPS1, MBTPS2, MCAT, MCEE, MECP2, MECR, MED1, MFSD2A, MGLL, MGST2, MGST3, MID1IP1, MIF, MIF, MIR132, MIR182, MIR185, MIR204, MIR27A, MIR27A, MIR27B, MIR29B1, MIR30C1, MIR33A, MIR548P, MIR766, MIR9-1, MIR96, MLXIPL, MLYCD, MOGAT1, MOGAT2, MOGAT3, MORC2, MPDU1, MPPE1, MSMO1, MT3, MTLN, MTM1, MTMR1, MTMR10, MTMR10, MTMR11, MTMR11, MTMR12, MTMR14, MTMR2, MTMR3, MTMR4, MTMR6, MTMR7, MTMR8, MTMR9, MTMR9, MTOR, MTTP, MVD, MVK, NAA40, NAAA, NAGA, NAPEPLD, NAPEPLD, NCEH1, NCOR1, NDUFAB1, NEU1, NEU1, NEU1, NEU1, NEU1, NEU1, NEU1, NEU1, NEU2, NEU3, NEU4, NEU4, NFE2L1, NFKB1, NKX1-1, NKX2-3, NOD2, NPC1, NPC1L1, NPC2, NR0B1, NR0B2, NR1D1, NR1D2, NR1H2, NR1H3, NR1H4, NR1I2, NR4A3, NR5A1, NR5A2, NSDHL, NSMAF, NUDT19, NUDT7, NUDT8, NUS1, OC90, OCRL, OLAH, OMA1, OPA3, ORMDL1, ORMDL2, ORMDL3, OSBP, OSBPL10, OSBPL1A, OSBPL2, OSBPL3, OSBPL5, OSBPL6, OSBPL7, OSBPL8, OSBPL9, OXSM, P2RX1, P2RX7, P2RY12, P3R3URF, PAFAH1B1, PAFAH1B2, PAFAH1B3, PAFAH2, PAM, PANK2, PBX1, PC, PCCA, PCCB, PCK1, PCK2, PCK2, PCSK9, PCYT1A, PCYT1B, PCYT2, PDE3A, PDE3B, PDGFA, PDGFB, PDGFRA, PDGFRB, PDK1, PDK2, PDK3, PDK4, PDSS1, PDSS2, PECR, PEDS1, PEMT, PER2, PEX13, PEX2, PEX5, PEX5, PEX7, PGAP1, PGAP2, PGAP3, PGAP4, PGAP6, PGP, PGS1, PHB2, PHYH, PI4K2A, PI4K2B, PI4KA, PI4KAP1, PI4KAP2, PI4KB, PIAS4, PIBF1, PIGA, PIGB, PIGC, PIGF, PIGG, PIGG, PIGH, PIGK, PIGL, PIGM, PIGN, PIGO, PIGP, PIGQ, PIGS, PIGT, PIGU, PIGV, PIGW, PIGW, PIGX, PIGY, PIGZ, PIK3C2A, PIK3C2B, PIK3C2G, PIK3C3, PIK3CA, PIK3CB, PIK3CD, PIK3CG, PIK3IP1, PIK3R1, PIK3R2, PIK3R3, PIK3R4, PIK3R6, PIKFYVE, PIP4K2A, PIP4K2B, PIP4K2B, PIP4K2C, PIP4P1, PIP4P2, PIP5K1A, PIP5K1B, PIP5K1C, PIP5KL1, PIPSL, PISD, PITPNA, PITPNB, PITPNM1, PITPNM2, PITPNM3, PLA1A, PLA2G10, PLA2G10, PLA2G12A, PLA2G12B, PLA2G15, PLA2G1B, PLA2G2A, PLA2G2C, PLA2G2D, PLA2G2E, PLA2G2F, PLA2G3, PLA2G4A, PLA2G4B, PLA2G4C, PLA2G4D, PLA2G4E, PLA2G4F, PLA2G5, PLA2G6, PLA2G7, PLAA, PLAAT1, PLAAT2, PLAAT3, PLAAT4, PLAAT5, PLAGL2, PLB1, PLBD1, PLBD2, PLCB1, PLCB2, PLCB3, PLCB4, PLCD1, PLCD3, PLCD4, PLCE1, PLCG1, PLCG2, PLCH1, PLCH2, PLCH2, PLCL1, PLCL2, PLCL2, PLCXD1, PLCXD2, PLCXD3, PLCZ1, PLD1, PLD2, PLD6, PLEK, PLEKHA1, PLGLB1, PLGLB2, PLIN1, PLIN5, PLP1, PLPP1, PLPP2, PLPP3, PLPP4, PLPP5, PLPP6, PLPPR1, PLPPR1, PLPPR2, PLPPR3, PLPPR3, PLPPR4, PLPPR5, PLSCR1, PLSCR3, PLSCR3, PLTP, PM20D1, PMVK, PNLIP, PNLIPRP1, PNLIPRP2, PNLIPRP3, PNPLA1, PNPLA2, PNPLA3, PNPLA4, PNPLA5, PNPLA6, PNPLA7, PNPLA8, PON1, POR, PORCN, PPARA, PPARD, PPARG, PPARGC1A, PPM1L, PPP2CA, PPP2R1A, PPP2R5A, PPT1, PRDX6, PRKAA1, PRKAA2, PRKAB1, PRKAB2, PRKAG1, PRKAG2, PRKAG3, PRKAR2B, PRKAR2B, PRKCD, PRKCE, PRKD1, PRKD2, PRKD3, PRLH, PRLR, PRMT3, PROCA1, PROX1, PRPF19, PRXL2B, PRXL2B, PSAP, PSAPL1, PTDSS1, PTDSS2, PTDSS2, PTEN, PTEN, PTGDS, PTGES, PTGES2, PTGES3, PTGIS, PTGR1, PTGR2, PTGS1, PTGS2, PTK2, PTK2B, PTPMT1, PTPMT1, PTPN11, PTPN22, PTPRN2, PTPRN2, QKI, RAB38, RAB7A, RARRES2, RBP1, RBP2, RBP3, RBP4, RDH10, RDH11, RDH12, RDH13, RDH13, RDH13, RDH13, RDH13, RDH13, RDH13, RDH13, RDH13, RDH13, RDH14, RDH16, RDH5, RDH8, REST, RETSAT, RFT1, RGN, RIDA, RLBP1, RNF213, RORA, RORC, RPE65, RUBCN, RUBCNL, RXRA, SACM1L, SAMD1, SAMD1, SAMD8, SC5D, SCAP, SCARB1, SCARF1, SCARF1, SCCPDH, SCD, SCD5, SCNN1B, SCP2, SCP2D1, SCPEP1, SCT, SCT, SDR16C5, SDR42E1, SDR42E2, SDS, SDSL, SEC14L2, SEL1L, SELENOI, SERAC1, SERINC1, SERINC2, SERINC3, SERINC4, SERINC5, SERPINA12, SERPINA3, SERPINA6, SERPINA6, SESN2, SESN2, SF1, SFTPB, SGMS1, SGMS2, SGPL1, SGPP1, SGPP1, SGPP2, SH3YL1, SHH, SIK1, SIRT1, SIRT2, SIRT2, SIRT3, SIRT4, SLA2, SLC16A1, SLC16A1, SLC16A11, SLC22A13, SLC22A24, SLC22A4, SLC25A17, SLC27A1, SLC27A2, SLC27A3, SLC27A3, SLC27A4, SLC27A5, SLC27A6, SLC35C1, SLC44A1, SLC44A2, SLC44A3, SLC44A4, SLC44A4, SLC44A4, SLC44A4, SLC44A4, SLC44A4, SLC44A4, SLC44A4, SLC44A5, SLC45A3, SMG1, SMPD1, SMPD2, SMPD3, SMPD4, SMPDL3A, SMPDL3B, SNAI1, SNAI2, SNCA, SNX17, SOAT1, SOAT2, SOCS1, SOCS2, SOCS3, SOCS4, SOCS5, SOCS6, SOCS7, SOCS7, SOD1, SORBS1, SORL1, SOX9, SPHK1, SPHK2, SPNS2, SPP1, SPTLC1, SPTLC2, SPTLC3, SPTSSA, SPTSSB, SQLE, SRC, SRD5A1, SRD5A2, SRD5A3, SREBF1, SREBF2, ST3GAL1, ST3GAL2, ST3GAL3, ST3GAL4, ST3GAL5, ST3GAL6, ST6GALNAC3, ST6GALNAC4, ST6GALNAC5, ST6GALNAC6, ST8SIA1, ST8SIA2, ST8SIA3, ST8SIA4, ST8SIA5, ST8SIA6, STAR, STARD3, STARD4, STAT5B, STOML2, STS, STUB1, SULT1A1, SULT1A2, SULT1A3, SULT1A4, SULT1C3, SULT1E1, SULT2A1, SULT2B1, SULT4A1, SUMF1, SYNJ1, SYNJ2, TAFAZZIN, TAMM41, TAMM41, TBL1XR1, TBXAS1, TECR, TECRL, TEK, TEX2, TGFB1, TH, THEM4, THEM5, THNSL2, THRA, THRSP, TIPARP, TLCD3B, TM6SF2, TM7SF2, TM9SF2, TMEM150A, TMEM38B, TMEM86B, TNF, TNF, TNF, TNF, TNF, TNF, TNF, TNF, TNFAIP8L3, TNFRSF1A, TNXB, TNXB, TNXB, TNXB, TNXB, TNXB, TNXB, TNXB, TPP1, TPRA1, TPTE, TPTE2, TREM2, TREX1, TRIB3, TRPV1, TSKU, TSPO, TTC39B, TTC7A, TTC7B, TTPA, TTR, TWIST1, TYRP1, TYSND1, UCP3, UGCG, UGT1A1, UGT1A10, UGT1A3, UGT1A4, UGT1A7, UGT1A8, UGT1A9, UGT2A1, UGT2A2, UGT2B10, UGT2B10, UGT2B11, UGT2B15, UGT2B15, UGT2B17, UGT2B28, UGT2B4, UGT2B7, UGT8, UVRAG, VAC14, VAPA, VAV2, VAV3, VLDLR, VSIG2, WASHC1, WDTC1, WNT10B, WNT4, XBP1, YWHAH, ZADH2, ZBTB20, ZFP69, ZMPSTE24, ZNF202, ZNF670, ZPBP2

**GOBP_LIPID_CATABOLIC_PROCESS：**AADAC, ABCB11, ABCB11, ABCD1, ABCD2, ABCD3, ABCD4, ABHD1, ABHD12, ABHD12B, ABHD16A, ABHD16A, ABHD16A, ABHD16A, ABHD16A, ABHD16A, ABHD16A, ABHD16B, ABHD2, ABHD3, ABHD4, ABHD5, ABHD6, ACAA1, ACAA2, ACACB, ACAD10, ACAD11, ACADL, ACADM, ACADS, ACADVL, ACAT1, ACAT2, ACER1, ACER2, ACER3, ACER3, ACOT8, ACOX1, ACOX2, ACOX3, ACOXL, ADIPOQ, ADORA1, ADRA2A, ADTRP, AIG1, AKR1B10, AKR1C3, AKR1D1, AKT1, AKT2, ALDH1L2, ALK, AMACR, ANGPTL3, AOAH, APOA1, APOA2, APOA4, APOA5, APOB, APOC1, APOC2, APOC3, APOE, ASAH1, ASAH2, ASAH2B, ASPG, AUH, BCO1, BCO2, BDH2, BSCL2, CDK4, CEL, CES1, CES1, CIDEA, CLPS, CLPSL1, CLPSL2, CNR1, CPS1, CPT1A, CPT1B, CPT1C, CPT2, CRABP1, CRAT, CROT, CRTC3, CYP19A1, CYP1A2, CYP1B1, CYP24A1, CYP26A1, CYP26B1, CYP26C1, CYP27A1, CYP27B1, CYP2W1, CYP39A1, CYP3A4, CYP46A1, CYP4A11, CYP4F11, CYP4F12, CYP4F2, CYP4F3, CYP7A1, DAGLA, DAGLB, DDHD1, DDHD2, DECR1, DECR2, DECR2, ECH1, ECH1, ECHDC1, ECHDC2, ECHS1, ECI1, ECI2, EHHADH, ENPP2, ENPP6, ENPP7, ETFA, ETFB, ETFBKMT, ETFDH, FAAH, FAAH2, FABP1, FGF21, FGF23, FMC1, FUCA1, GALC, GBA, GBA, GBA2, GBA3, GCDH, GDE1, GDPD1, GDPD3, GLA, GLYATL2, GM2A, GPCPD1, GPIHBP1, GPLD1, HACL1, HADH, HADHA, HADHB, HAO1, HCAR1, HCAR2, HEXA, HEXB, HINT2, HSD11B1, HSD17B10, HSD17B11, HSD17B14, HSD17B4, HSD17B6, IAH1, IDH1, IL1B, ILVBL, INPP5F, INS, IRS1, IRS2, IVD, LDAH, LDLR, LEP, LGALS12, LIPA, LIPC, LIPE, LIPF, LIPG, LIPH, LIPI, LIPJ, LIPK, LIPM, LIPN, LONP2, LPIN1, LPIN2, LPIN3, LPL, LRCOL1, LYPLA2, MCAT, MCEE, MFSD2A, MGLL, MGST2, MLYCD, MT3, MTLN, NAAA, NAGA, NAPEPLD, NAPEPLD, NCEH1, NEU1, NEU1, NEU1, NEU1, NEU1, NEU1, NEU1, NEU1, NEU2, NEU3, NEU4, NEU4, NUDT19, NUDT7, NUDT8, OC90, PAFAH1B1, PAFAH1B2, PAFAH1B3, PAFAH2, PCCA, PCCB, PCK1, PCK2, PCK2, PDE3B, PEX13, PEX2, PEX5, PEX5, PEX7, PHYH, PIK3CG, PLA1A, PLA2G10, PLA2G10, PLA2G12A, PLA2G12B, PLA2G15, PLA2G1B, PLA2G2A, PLA2G2C, PLA2G2D, PLA2G2E, PLA2G2F, PLA2G4A, PLA2G4B, PLA2G4C, PLA2G4D, PLA2G4E, PLA2G4F, PLA2G5, PLA2G6, PLA2G7, PLAAT1, PLAAT2, PLAAT3, PLAAT4, PLB1, PLBD1, PLBD2, PLCB1, PLCB2, PLCB3, PLCB4, PLCD1, PLCD3, PLCD4, PLCE1, PLCG1, PLCG2, PLCH1, PLCH2, PLCH2, PLCXD2, PLCXD3, PLCZ1, PLD1, PLD2, PLD6, PLIN1, PLIN5, PLPP6, PNLIP, PNLIPRP2, PNLIPRP3, PNPLA1, PNPLA2, PNPLA3, PNPLA4, PNPLA5, PNPLA6, PNPLA7, PNPLA8, PPARA, PPARD, PPT1, PRDX6, PRKAA1, PRKCD, PRKCE, RAB7A, RARRES2, SCARB1, SCARF1, SCARF1, SCP2, SCT, SCT, SESN2, SESN2, SGPL1, SIRT2, SIRT2, SLC25A17, SLC27A2, SLC27A4, SMPD1, SMPD2, SMPD3, SMPD4, SMPDL3A, SMPDL3B, SNX17, SORL1, SPHK1, SPP1, SRD5A1, SRD5A2, SRD5A3, STS, SULT1E1, SULT2A1, TBL1XR1, THRA, TNF,, TWIST1, TYSND1, YWHAH, ZPBP2

**GOBP_LIPID_BIOSYNTHETIC_PROCESS：**A3GALT2, A4GALT, ABCA2, ABCA3, ABCA8, ABCB11, ABCB11, ABCD1, ABCD2, ABCD3, ABCG1, ABCG4, ABHD1, ABHD2, ABHD3, ABHD4, ABHD5, ABHD6, ABHD8, ACAA2, ACACA, ACACA, ACACB, ACADL, ACADVL, ACAT1, ACBD3, ACER1, ACER2, ACER3, ACER3, ACLY, ACOT4, ACOT7, ACOT8, ACP6, ACSBG1, ACSBG2, ACSF3, ACSL1, ACSL3, ACSL5, ACSL6, ACSM1, ACSM2A, ACSM2B, ACSM3, ACSM4, ACSM5, ACSM6, ACSS1, ACSS2, ADGRF5, ADM, AGK, AGK, AGMO, AGPAT1, AGPAT1, AGPAT1, AGPAT1, AGPAT1, AGPAT1, AGPAT1, AGPAT2, AGPAT3, AGPAT4, AGPAT5, AGPAT5, AGPS, AJUBA, AKR1B1, AKR1B15, AKR1C3, AKR1C4, AKR1D1, AKT1, ALDH1A2, ALDH1A3, ALDH3B1, ALDH3B2, ALDH8A1, ALOX12, ALOX12B, ALOX15, ALOX15B, ALOX5, ALOX5, ALOXE3, AMACR, ANG, ANXA1, APOA1, APOA2, APOA4, APOA5, APOB, APOC1, APOC2, APOC3, APOE, ARMC5, ARV1, ASAH1, ASAH2, ASAH2B, ASXL3, ATG14, ATM, ATP1A1, AVIL, AVP, AVPR1A, AWAT2, B3GALT1, B3GALT2, B3GALT4, B3GALT4, B3GALT4, B3GALT4, B3GALT4, B3GNT5, B4GALNT1, B4GALT3, B4GALT4, B4GALT5, B4GALT6, BAAT, BAAT, BCO1, BECN1, BMP2, BMP5, BMP6, BMX, BPNT1, BPNT2, BRCA1, C3, CACNA1H, CAPN2, CBR1, CBR4, CCDC3, CCN1, CD74, CDIPT, CDK4, CDS1, CDS2, CEACAM1, CEPT1, CERKL, CERS1, CERS2, CERS3, CERS4, CERS5, CERS6, CES1, CES1, CFTR, CGA, CH25H, CHAT, CHKA, CHKB, CHP1, CHPT1, CISH, CLCN2, CLN3, CLN8, CLN8, CNEP1R1, COQ2, CRABP2, CREB1, CREBL2, CRH, CRLS1, CRPPA, CSNK1G2, CTDNEP1, CTDNEP1, CWH43, CYB5R1, CYB5R2, CYB5R3, CYP11A1, CYP11A1, CYP11B1, CYP11B2, CYP17A1, CYP19A1, CYP1A1, CYP1A2, CYP21A2, CYP21A2, CYP21A2, CYP21A2, CYP21A2, CYP21A2, CYP27A1, CYP27B1, CYP2C8, CYP2C9, CYP2D6, CYP2D6, CYP2D6, CYP2D6, CYP2D6, CYP2D6, CYP2E1, CYP2R1, CYP39A1, CYP3A4, CYP3A7, CYP46A1, CYP4F22, CYP51A1, CYP7A1, CYP7B1, CYP8B1, DAB2, DAGLB, DDX20, DECR2, DECR2, DEGS1, DEGS2, DGAT1, DGAT1, DGAT1, DGAT2, DGAT2L6, DGKA, DGKB, DGKD, DGKD, DGKE, DGKG, DGKH, DGKQ, DGKZ, DHCR24, DHCR7, DHDDS, DHH, DHRS11, DHRS11, DHRS7B, DHRS9, DKK3, DKKL1, DOLK, DOLPP1, DPM1, DPM2, DPM3, EBP, EDN1, EDN2, EFR3A, EFR3B, EGR1, EIF6, ELOVL1, ELOVL2, ELOVL3, ELOVL4, ELOVL5, ELOVL6, ELOVL7, ENPP7, ENSG00000205794, ERG28, ERLIN1, ERLIN2, ETNK1, ETNK2, FA2H, FABP3, FABP5, FADS1, FADS2, FADS2B, FADS3, FADS6, FAM126A, FAM126B, FAM3A, FAR1, FASN, FAXDC2, FBXW7, FDFT1, FDFT1, FDPS, FDX1, FDXR, FGF1, FGF19, FGF2, FGFR4, FIG4, FITM1, FITM1, FITM2, FSHB, FUT6, FUT9, G6PD, GAL3ST1, GAL3ST2, GAL3ST2, GAL3ST3, GAL3ST4, GBA, GBA, GBGT1, GFI1, GGPS1, GIP, GK, GNPAT, GPAA1, GPAM, GPAT2, GPAT3, GPAT4, GPER1, GPLD1, GPX4, GSTM1, GSTM2, GSTM4, GSTP1, H6PD, HACD1, HACD2, HACD3, HACD4, HDHD5, HEXB, HINT2, HMGCR, HMGCS1, HMGCS2, HPGDS, HSD17B1, HSD17B10, HSD17B11, HSD17B12, HSD17B13, HSD17B2, HSD17B3, HSD17B6, HSD17B7, HSD17B8, HSD17B8, HSD17B8, HSD17B8, HSD17B8, HSD17B8, HSD3B1, HSD3B2, HSD3B7, HTR2A, HTR2B, HTR2C, HULC, IDH1, IDI1, IDI2, IFNG, IGF1R, IGFBP7, IL1A, IL1B, IMPA1, IMPA2, INPP1, INPP4A, INPP4B, INPP5D, INPP5D, INPP5E, INPP5F, INPP5J, INPP5K, INPPL1, INS, INSIG1, INSIG2, IP6K1, IP6K2, IP6K3, IPMK, ISYNA1, ITPKA, ITPKB, ITPKC, KDSR, LARGE1, LBR, LCAT, LCLAT1, LDLR, LEP, LHB, LIAS, LIPC, LIPG, LIPH, LIPI, LPCAT1, LPCAT1, LPCAT2, LPCAT3, LPCAT4, LPGAT1, LPIN1, LPIN2, LPIN3, LPL, LSS, LSS, LTC4S, LTC4S, MALRD1, MBOAT1, MBOAT2, MBOAT7, MBOAT7, MBOAT7, MBOAT7, MBOAT7, MBOAT7, MBOAT7, MBOAT7, MBOAT7, MBOAT7, MBTPS1, MBTPS2, MCAT, MECR, MED1, MFSD2A, MGLL, MID1IP1, MIF, MIF, MIR132, MIR182, MIR185, MIR204, MIR29B1, MIR30C1, MIR33A, MIR548P, MIR766, MIR9-1, MIR96, MLXIPL, MLYCD, MOGAT1, MOGAT2, MOGAT3, MPPE1, MSMO1, MTM1, MTMR1, MTMR14, MTMR2, MTMR3, MTMR4, MTMR6, MTMR7, MTOR, MVD, MVK, NDUFAB1, NFKB1, NPC1L1, NR0B1, NR1D1, NR1H2, NR1H3, NR1H4, NR5A1, NSDHL, NUS1, OCRL, OLAH, ORMDL1, ORMDL2, ORMDL3, OSBP, OSBPL1A, OSBPL2, OSBPL3, OSBPL6, OSBPL7, OSBPL9, OXSM, P2RX1, P2RX7, P3R3URF, PAM, PBX1, PCK1, PCK2, PCK2, PCYT1A, PCYT1B, PCYT2, PDGFA, PDGFB, PDK4, PDSS1, PDSS2, PECR, PEDS1, PEMT, PEX7, PGAP1, PGAP2, PGAP3, PGAP4, PGS1, PI4K2A, PI4K2B, PI4KA, PI4KAP1, PI4KAP2, PI4KB, PIBF1, PIGA, PIGB, PIGC, PIGF, PIGG, PIGG, PIGH, PIGK, PIGL, PIGM, PIGN, PIGO, PIGP, PIGQ, PIGS, PIGT, PIGU, PIGV, PIGW, PIGW, PIGX, PIGY, PIGZ, PIK3C2A, PIK3C2B, PIK3C2G, PIK3C3, PIK3CA, PIK3CB, PIK3CD, PIK3CG, PIK3R1, PIK3R2, PIK3R3, PIK3R4, PIK3R6, PIKFYVE, PIP4K2A, PIP4K2B, PIP4K2B, PIP4K2C, PIP5K1A, PIP5K1B, PIP5K1C, PIP5KL1, PIPSL, PISD, PITPNM1, PITPNM2, PITPNM3, PLA2G10, PLA2G10, PLA2G15, PLA2G1B, PLA2G3, PLA2G4A, PLA2G4C, PLA2G4F, PLA2G6, PLAAT3, PLCE1, PLCG2, PLD1, PLD2, PLIN5, PLP1, PLPP1, PLPP2, PLPP3, PLPP6, PLSCR1, PLSCR3, PLSCR3, PMVK, PNPLA1, PNPLA2, PNPLA3, PNPLA8, POR, PPARA, PPARD, PPM1L, PRKAA1, PRKAA2, PRKAB1, PRKAB2, PRKAG1, PRKAG2, PRKAG3, PRKCD, PRKD1, PRKD2, PRKD3, PRLR, PRMT3, PROX1, PRPF19, PRXL2B, PRXL2B, PTDSS1, PTDSS2, PTDSS2, PTEN, PTEN, PTGDS, PTGES, PTGES2, PTGES3, PTGIS, PTGS1, PTGS2, PTPMT1, PTPMT1, QKI, RAB38, RBP1, RDH10, RDH8, REST, RGN, RPE65, SACM1L, SAMD8, SC5D, SCAP, SCARB1, SCCPDH, SCD, SCD5, SCP2, SCP2D1, SDR42E1, SDR42E2, SEC14L2, SELENOI, SERAC1, SERINC1, SERINC4, SERINC5, SERPINA12, SF1, SGMS1, SGMS2, SGPL1, SGPP1, SGPP1, SGPP2, SH3YL1, SIK1, SIRT1, SIRT3, SIRT4, SLA2, SLC27A1, SLC27A2, SLC27A5, SLC44A1, SLC44A2, SLC44A3, SLC44A4, SLC44A4, SLC44A4, SLC44A4, SLC44A4, SLC44A4, SLC44A4, SLC44A4, SLC44A5, SLC45A3, SMG1, SMPD1, SMPD2, SMPD4, SNAI1, SNAI2, SOCS1, SOCS2, SOCS3, SOCS4, SOCS5, SOCS6, SOCS7, SOCS7, SOD1, SORBS1, SPHK1, SPHK2, SPNS2, SPTLC1, SPTLC2, SPTLC3, SPTSSA, SPTSSB, SQLE, SRD5A1, SRD5A2, SRD5A3, SREBF1, ST3GAL1, ST3GAL2, ST3GAL3, ST3GAL4, ST3GAL5, ST6GALNAC3, ST6GALNAC4, ST6GALNAC5, ST6GALNAC6, ST8SIA1, ST8SIA2, ST8SIA3, ST8SIA4, ST8SIA5, ST8SIA6, STAR, STARD3, STARD4, SYNJ1, SYNJ2, TAFAZZIN, TAMM41, TAMM41, TBXAS1, TECR, TECRL, THNSL2, THRSP, TLCD3B, TM7SF2, TM9SF2, TMEM150A, TMEM38B, TNF, TNF, TNF, TNF, TNF, TNF, TNF, TNF, TPTE2, TREX1, TRIB3, TSPO, TTC7A, TTC7B, UGCG, UGT8, UVRAG, VAC14, VAPA, WDTC1, WNT4, XBP1, ZBTB20

**GOBP_LIPID_OXIDATION：**ABCB11, ABCB11, ABCD1, ABCD2, ABCD3, ABCD4, ACAA1, ACAA2, ACACB, ACAD10, ACAD11, ACADL, ACADM, ACADS, ACADVL, ACAT1, ACAT2, ACOT8, ACOX1, ACOX2, ACOX3, ACOXL, ACSM1, ADH4, ADH5, ADH7, ADIPOQ, ADIPOR1, ADIPOR2, ADIPOR2, AKT1, AKT2, ALDH1L2, ALOX12, ALOX12B, ALOX15, ALOX15B, ALOX5, ALOX5, AMACR, APOD, APPL2, AUH, BDH2, CNR1, CPT1A, CPT1B, CPT1C, CPT2, CRAT, CROT, CYGB, CYP24A1, CYP4F2, CYP4V2, DECR1, DECR2, DECR2, DGAT2, ECH1, ECH1, ECHDC1, ECHDC2, ECHS1, ECI1, ECI2, EHHADH, ETFA, ETFB, ETFBKMT, ETFDH, FABP1, FABP3, GCDH, HACL1, HADH, HADHA, HADHB, HAO1, HAO2, HSD17B10, HSD17B4, ILVBL, IRS1, IRS2, IVD, LEP, LONP2, MAPK14, MCAT, MFSD2A, MLYCD, MTLN, NR4A3, PDK4, PEX13, PEX2, PEX5, PEX5, PEX7, PHYH, PLA2G7, PLIN5, POR, PPARA, PPARD, PPARGC1A, PRKAA1, PRKAG2, SAMD1, SAMD1, SCP2, SESN2, SESN2, SIRT4, SLC25A17, SLC27A2, SOX9, TWIST1, TYSND1

**GOBP_LIPID_STORAGE：**ABCA1, ABCG1, ABHD5, ACACB, ACVR1C, ALKBH7, ANGPTL3, APOA1, APOB, APOC4, APOE, AUP1, B4GALNT1, BSCL2, C3, CAV1, CD36, CDS1, CDS2, CES1, CES1, CIDEA, CPT1A, CRP, CRY1, CRY2, DGAT1, DGAT1, DGAT1, DGAT2, EHD1, ENPP1, FBXW7, FFAR2, FITM1, FITM1, FITM2, FTO, GBA, GBA, GM2A, HEXB, HILPDA, IKBKE, IL1B, IL6, ITGAV, ITGB3, LDAF1, LDAF1, LDAH, LEP, LPL, MEST, MIR10B, MIR144, MIR146A, MIR34A, MSR1, NFKB1, NFKBIA, NR1H2, NR1H3, NRIP1, OSBPL11, OSBPL8, PLA2G10, PLA2G10, PLA2G4C, PLIN2, PLIN3, PLIN5, PNPLA2, PPARA, PPARD, PPARG, PTPN2, RNF213, SCARB1, SIRT1, SMIM22, SOAT1, SQLE, SREBF1, SREBF2, STARD4, STAT5B, TNF, TNF, TNF, TNF, TNF, TNF, TNF, TNF, TREM2, TTC39B, VSTM2A, ZC3H12A, ZFYVE1

**GOBP_PHOSPHOLIPID_METABOLIC_PROCESS：**ABCA2, ABCA3, ABCA8, ABHD12, ABHD12B, ABHD16A, ABHD16A, ABHD16A, ABHD16A, ABHD16A, ABHD16A, ABHD16A, ABHD16B, ABHD3, ABHD4, ABHD5, ABHD6, ABHD8, ACP6, ACSL3, ADGRF5, AGPAT1, AGPAT1, AGPAT1, AGPAT1, AGPAT1, AGPAT1, AGPAT1, AGPAT2, AGPAT3, AGPAT4, AGPAT5, AGPAT5, AJUBA, ALOX15, ALOX15B, ANGPTL3, APOA1, APOA2, APOA4, APOA5, APOC1, APOC2, ATG14, ATM, BECN1, BLOC1S6, BMX, BPNT1, BPNT2, CAPN2, CDIPT, CDS1, CDS2, CEPT1, CETP, CHAT, CHKA, CHKB, CHP1, CHPT1, CHRM5, CISH, CLN3, CLN8, CLN8, CRLS1, CSF1R, CWH43, CYP2W1, DBI, DGKA, DGKB, DGKD, DGKD, DGKE, DGKG, DGKH, DGKQ, DGKZ, DHDDS, DHRS7B, DNAJC19, DOLK, DOLPP1, DPM1, DPM2, DPM3, EFR3A, EFR3B, ENPP2, ENPP6, ENPP7, ETNK1, ETNK2, FABP3, FABP5, FADS1, FAM126A, FAM126B, FAR1, FDFT1, FDFT1, FDPS, FGF2, FIG4, FITM1, FITM1, FITM2, GALR2, GATA6, GDE1, GDPD1, GDPD3, GGPS1, GNB3, GNPAT, GPAA1, GPAM, GPAT2, GPAT3, GPAT4, GPCPD1, GPLD1, GPX4, HADHA, HDHD5, HEXB, HMGCS1, HMGCS2, HTR2A, HTR2B, HTR2C, IDH1, IDI1, IDI2, IMPA1, IMPA2, INPP1, INPP4A, INPP4B, INPP5A, INPP5B, INPP5D, INPP5D, INPP5E, INPP5F, INPP5J, INPP5K, INPPL1, IP6K1, IP6K2, IP6K3, IPMK, ISYNA1, ITPKA, ITPKB, ITPKC, LCAT, LCLAT1, LDLR, LGALS13, LIPC, LIPG, LIPH, LIPI, LPCAT1, LPCAT1, LPCAT2, LPCAT3, LPCAT4, LPGAT1, LPIN1, LPL, MBOAT1, MBOAT2, MBOAT7, MBOAT7, MBOAT7, MBOAT7, MBOAT7, MBOAT7, MBOAT7, MBOAT7, MBOAT7, MBOAT7, MECP2, MFSD2A, MIR30C1, MPPE1, MTM1, MTMR1, MTMR10, MTMR10, MTMR11, MTMR11, MTMR12, MTMR14, MTMR2, MTMR3, MTMR4, MTMR6, MTMR7, MTMR8, MTMR9, MTMR9, MVD, MVK, NAAA, NAPEPLD, NAPEPLD, NR1H2, NR1H3, NR1H4, NUS1, OC90, OCRL, ORMDL3, OSBP, OSBPL10, OSBPL5, OSBPL8, P3R3URF, PAFAH1B1, PCSK9, PCYT1A, PCYT1B, PCYT2, PDGFA, PDGFB, PDGFRB, PEMT, PGAP1, PGAP2, PGAP3, PGAP4, PGP, PGS1, PHB2, PI4K2A, PI4K2B, PI4KA, PI4KAP1, PI4KAP2, PI4KB, PIGA, PIGB, PIGC, PIGF, PIGG, PIGG, PIGH, PIGK, PIGL, PIGM, PIGN, PIGO, PIGP, PIGQ, PIGS, PIGT, PIGU, PIGV, PIGW, PIGW, PIGX, PIGY, PIGZ, PIK3C2A, PIK3C2B, PIK3C2G, PIK3C3, PIK3CA, PIK3CB, PIK3CD, PIK3CG, PIK3R1, PIK3R2, PIK3R3, PIK3R4, PIK3R6, PIKFYVE, PIP4K2A, PIP4K2B, PIP4K2B, PIP4K2C, PIP4P1, PIP4P2, PIP5K1A, PIP5K1B, PIP5K1C, PIP5KL1, PIPSL, PISD, PITPNM1, PITPNM2, PITPNM3, PLA1A, PLA2G10, PLA2G10, PLA2G12A, PLA2G12B, PLA2G15, PLA2G1B, PLA2G2A, PLA2G2C, PLA2G2D, PLA2G2E, PLA2G2F, PLA2G3, PLA2G4A, PLA2G4B, PLA2G4C, PLA2G4D, PLA2G4E, PLA2G4F, PLA2G5, PLA2G6, PLA2G7, PLAA, PLAAT1, PLAAT2, PLAAT3, PLAAT4, PLAAT5, PLB1, PLBD1, PLBD2, PLCB1, PLCB2, PLCB3, PLCD1, PLCD4, PLCG1, PLCG2, PLCH2, PLCH2, PLD1, PLD2, PLEK, PLPP1, PLPP2, PLPP3, PLPP4, PLPP5, PLPP6, PLPPR1, PLPPR1, PLPPR2, PLPPR3, PLPPR3, PLPPR4, PLPPR5, PLSCR1, PLSCR3, PLSCR3, PMVK, PNLIPRP2, PNPLA3, PNPLA6, PNPLA7, PNPLA8, PON1, PPARD, PRDX6, PRKCD, PROCA1, PTDSS1, PTDSS2, PTDSS2, PTEN, PTEN, PTPMT1, PTPMT1, RAB38, SACM1L, SAMD8, SCARB1, SELENOI, SERAC1, SERINC1, SERINC2, SERINC3, SERINC4, SERINC5, SGMS1, SGMS2, SGPP1, SGPP1, SH3YL1, SLA2, SLC27A1, SLC44A1, SLC44A2, SLC44A3, SLC44A4, SLC44A4, SLC44A4, SLC44A4, SLC44A4, SLC44A4, SLC44A4, SLC44A4, SLC44A5, SMG1, SMPD1, SMPD2, SMPD3, SMPD4, SMPDL3A, SMPDL3B, SNCA, SOCS1, SOCS2, SOCS3, SOCS4, SOCS5, SOCS6, SOCS7, SOCS7, SPHK2, SPTLC1, SPTLC2, SRD5A3, STOML2, SYNJ1, SYNJ2, TAFAZZIN, TAMM41, TAMM41, THEM5, TMEM150A, TMEM38B, TMEM86B, TNFAIP8L3, TPTE, TPTE2, TTC7A, TTC7B, UVRAG, VAC14, VAPA

**GOBP_FATTY_ACID_METABOLIC_PROCESS：**AACS, AASDH, ABCB11, ABCB11, ABCD1, ABCD2, ABCD3, ABCD4, ABHD1, ABHD12, ABHD2, ABHD3, ABHD5, ABHD6, ACAA1, ACAA2, ACACA, ACACA, ACACB, ACAD10, ACAD11, ACAD9, ACADL, ACADM, ACADS, ACADSB, ACADVL, ACAT1, ACAT2, ACBD4, ACBD5, ACBD7, ACLY, ACOT1, ACOT11, ACOT12, ACOT2, ACOT4, ACOT6, ACOT7, ACOT8, ACOX1, ACOX2, ACOX3, ACOXL, ACSBG1, ACSBG2, ACSF2, ACSF3, ACSL1, ACSL3, ACSL4, ACSL5, ACSL6, ACSM1, ACSM2A, ACSM2B, ACSM3, ACSM4, ACSM5, ACSM6, ACSS1, ADH4, ADH5, ADH7, ADIPOQ, ADIPOR1, ADIPOR2, ADIPOR2, ADTRP, AIG1, AKR1B1, AKR1C1, AKR1C2, AKR1C3, AKR1C4, AKT1, AKT2, ALDH1L2, ALDH3A2, ALKBH7, ALOX12, ALOX12B, ALOX15, ALOX15B, ALOX5, ALOX5, ALOX5AP, ALOXE3, AMACR, ANGPTL3, ANXA1, AOAH, APOA1, APOA4, APOA5, APOC1, APOC2, APOC3, APPL2, ASAH1, ASAH2, ASAH2B, ATP6V1B1, AUH, AVP, AVPR1A, AWAT1, BAAT, BAAT, BDH2, BRCA1, C3, CAV1, CBR1, CBR4, CD36, CD74, CEACAM1, CEL, CES1, CES1, CES2, CNR1, CPT1A, CPT1B, CPT1C, CPT2, CRAT, CROT, CRYL1, CYGB, CYP1A1, CYP1A2, CYP1B1, CYP24A1, CYP2A13, CYP2A6, CYP2A7, CYP2B6, CYP2B6, CYP2C18, CYP2C19, CYP2C8, CYP2C9, CYP2D6, CYP2D6, CYP2D6, CYP2D6, CYP2D6, CYP2D6, CYP2D7, CYP2D7, CYP2D7, CYP2D7, CYP2E1, CYP2F1, CYP2G1P, CYP2J2, CYP2S1, CYP2U1, CYP3A4, CYP4A11, CYP4A22, CYP4B1, CYP4F11, CYP4F12, CYP4F2, CYP4F3, CYP4F8, CYP4V2, CYP4Z1, CYP7A1, DAGLA, DAGLB, DBI, DECR1, DECR2, DECR2, DEGS1, DGAT2, ECH1, ECH1, ECHDC1, ECHDC2, ECHDC3, ECHS1, ECI1, ECI2, EDN1, EDN2, EHHADH, EIF6, ELOVL1, ELOVL2, ELOVL3, ELOVL4, ELOVL5, ELOVL6, ELOVL7, ENSG00000205794, EPHX1, ERFE, ERLIN1, ERLIN2, ETFA, ETFB, ETFBKMT, ETFDH, FA2H, FAAH, FAAH2, FABP1, FABP2, FABP3, FABP5, FADS1, FADS2, FADS2B, FADS3, FADS6, FASN, GCDH, GGT1, GGT5, GHSR, GIP, GLYATL2, GNPAT, GPAM, GPAT2, GPAT4, GPX1, GPX4, GSTA1, GSTM1, GSTM2, GSTM4, GSTP1, HACD1, HACD2, HACD3, HACD4, HACL1, HADH, HADHA, HADHB, HAO1, HAO2, HPGD, HPGDS, HSD17B10, HSD17B12, HSD17B4, HSD17B8, HSD17B8, HSD17B8, HSD17B8, HSD17B8, HSD17B8, HTD2, IL1B, ILVBL, INS, INSIG1, INSIG2, IRS1, IRS2, IVD, LEP, LIAS, LIPC, LIPG, LIPH, LIPI, LONP2, LPGAT1, LPIN1, LPIN2, LPIN3, LPL, LTC4S, LTC4S, LYPLA1, LYPLA2, MAPK14, MBLAC2, MCAT, MCEE, MECR, MFSD2A, MGLL, MID1IP1, MIF, MIF, MIR132, MIR182, MIR204, MIR30C1, MIR33A, MIR548P, MIR766, MIR96, MLXIPL, MLYCD, MORC2, MSMO1, MTLN, NAAA, NCOR1, NDUFAB1, NR1H2, NR1H3, NR4A3, NUDT19, NUDT7, NUDT8, OLAH, OXSM, PAM, PANK2, PCCA, PCCB, PCK1, PCK2, PCK2, PDK1, PDK2, PDK3, PDK4, PECR, PEDS1, PER2, PEX13, PEX2, PEX5, PEX5, PEX7, PHYH, PIBF1, PLA2G10, PLA2G10, PLA2G15, PLA2G1B, PLA2G2F, PLA2G3, PLA2G4A, PLA2G4B, PLA2G4C, PLA2G4D, PLA2G4F, PLA2G5, PLAA, PLIN5, PLP1, PM20D1, PNPLA3, PNPLA8, POR, PPARA, PPARD, PPARG, PPARGC1A, PRKAA1, PRKAA2, PRKAB1, PRKAB2, PRKAG1, PRKAG2, PRKAG3, PRKAR2B, PRKAR2B, PRXL2B, PRXL2B, PTGDS, PTGES, PTGES2, PTGES3, PTGIS, PTGR1, PTGR2, PTGS1, PTGS2, QKI, RGN, SCAP, SCD, SCD5, SCP2, SESN2, SESN2, SGPL1, SIRT1, SIRT4, SLC22A13, SLC25A17, SLC27A1, SLC27A2, SLC27A3, SLC27A3, SLC27A4, SLC27A5, SLC27A6, SLC45A3, SNCA, SOX9, SREBF1, TBXAS1, TECR, TECRL, TH, THEM4, THEM5, THNSL2, TNFRSF1A, TNXB, TNXB, TNXB, TNXB, TNXB, TNXB, TNXB, TNXB, TREX1, TRIB3, TWIST1, TYRP1, TYSND1, UCP3, UGT1A8, WDTC1, XBP1, ZADH2

**KEGG_GLYCEROLIPID_METABOLISM：**AGK, AGK, AGPAT1, AGPAT1, AGPAT1, AGPAT1, AGPAT1, AGPAT1, AGPAT1, AGPAT2, AGPAT3, AGPAT4, AKR1A1, AKR1B1, ALDH1B1, ALDH2, ALDH3A2, ALDH7A1, ALDH9A1, AWAT2, CEL, DGAT1, DGAT1, DGAT1, DGAT2, DGKA, DGKB, DGKD, DGKD, DGKE, DGKG, DGKH, DGKI, DGKQ, DGKZ, GK, GK2, GLA, GLYCTK, GPAM, GPAT2, GPAT3, GPAT4, LCLAT1, LIPC, LIPF, LIPG, LPL, MBOAT1, MBOAT2, MGLL, PLPP1, PLPP2, PLPP3, PNLIP, PNLIPRP1, PNLIPRP2, PNPLA3, TKFC
