# Supplementary material for: Integration of single-cell and bulk RNA-seq via machine learning to reveal ferroptosis- and lipid metabolism-driven immune landscape heterogeneity and predict immunotherapy response in colon cancer
Source: Front Immunol. 2025 Dec 5;16:1699079. doi: 10.3389/fimmu.2025.1699079 (PMC12714941; doi:10.3389/fimmu.2025.1699079)
Supplement: Supplementary file 24 [file Table9.docx]

FABP4,TRARG1,SNCG,CALB2,SERPINE1,UCHL1,ADAMTS4,ARL4C,LAMP5,FSTL3,ANGPTL4,DAPK1,SLC2A3,ADAM8,CPA3,SLC12A2,NEBL,PPA1,EPOP,AXIN2,ASPHD1,TMEM220,SEZ6L2,ANXA3,WDR72,RIMS4,RAB15,EPHB2,SLC38A5,TUBA1C,PTGDR2,PLCD1,KCNE3,SHH,ASPG,EFNA4,CDC25C,TMEM72,GRB14,CEACAM6,NMRAL2P,SEL1L3,CLCA4,ANKRD22,PLPP2,SERPINA1,NOX1,EDN3,SPINK4,BMP5,NOS2,MYB,SPINK1,DNASE1L3,TNFRSF17,CEACAM5,LYPD8,LINC00261,GJB1,UGT2A3
