# Supplementary material for: Integration of single-cell and bulk RNA-seq via machine learning to reveal ferroptosis- and lipid metabolism-driven immune landscape heterogeneity and predict immunotherapy response in colon cancer
Source: Front Immunol. 2025 Dec 5;16:1699079. doi: 10.3389/fimmu.2025.1699079 (PMC12714941; doi:10.3389/fimmu.2025.1699079)
Supplement: Supplementary file 25 [file Table10.docx]

variable permutation dropout_loss label

SPINK4 0 0.407680792104442 SVM

ANGPTL4 0 0.407751832108689 SVM

NOX1 0 0.407752821785926 SVM

DNASE1L3 0 0.407778182879732 SVM

UGT2A3 0 0.407936920689856 SVM

CDC25C 0 0.40794328031302 SVM

SNCG 0 0.4079615739095 SVM

ANXA3 0 0.40797901733848 SVM

DAPK1 0 0.407988831013466 SVM

PTGDR2 0 0.408180443543303 SVM

CEACAM6 0 0.408201589327346 SVM

BMP5 0 0.408226678226159 SVM

TUBA1C 0 0.408377057202259 SVM

PLPP2 0 0.408511138545385 SVM

SERPINA1 0 0.408547252498304 SVM

SEZ6L2 0 0.408570909481607 SVM

ANKRD22 0 0.408593614427749 SVM

SLC38A5 0 0.408631595972048 SVM

GRB14 0 0.408680014310062 SVM

WDR72 0 0.408876949333287 SVM

ASPG 0 0.40904034361609 SVM

TNFRSF17 0 0.409501870507239 SVM

EDN3 0 0.409664751952998 SVM

FABP4 0 0.409709014898766 SVM

LINC00261 0 0.410297952089199 SVM

NOS2 0 0.410330973715321 SVM

TMEM220 0 0.411009156338992 SVM

TRARG1 0 0.41283583368667 SVM

NMRAL2P 0 0.415073048638506 SVM

CPA3 0 0.415555413441388 SVM
