# Supplementary material for: Integration of single-cell and bulk RNA-seq via machine learning to reveal ferroptosis- and lipid metabolism-driven immune landscape heterogeneity and predict immunotherapy response in colon cancer
Source: Front Immunol. 2025 Dec 5;16:1699079. doi: 10.3389/fimmu.2025.1699079 (PMC12714941; doi:10.3389/fimmu.2025.1699079)
Supplement: Supplementary file 26 [file Table11.docx]

variable permutation dropout_loss label

ASPHD1 0 0.393036619844694 RF

ANKRD22 0 0.393040371519129 RF

CLCA4 0 0.393052798443802 RF

TMEM72 0 0.393099597758906 RF

ANGPTL4 0 0.393146119220966 RF

CEACAM6 0 0.393150657570021 RF

UGT2A3 0 0.393156347228528 RF

LAMP5 0 0.393283063116639 RF

SLC38A5 0 0.393291889375562 RF

PPA1 0 0.393297053952176 RF

EDN3 0 0.393341177547208 RF

LINC00261 0 0.393428349951465 RF

CEACAM5 0 0.393447978291718 RF

ASPG 0 0.393515551306985 RF

ANXA3 0 0.393544761896774 RF

DNASE1L3 0 0.393544886544574 RF

TUBA1C 0 0.393665244640396 RF

NEBL 0 0.393778657075873 RF

EPOP 0 0.393820001493416 RF

FABP4 0 0.393845768282966 RF

CDC25C 0 0.393888990367396 RF

BMP5 0 0.393967314449126 RF

TRARG1 0 0.393991685218135 RF

NOS2 0 0.394075668436742 RF

CPA3 0 0.394513969072014 RF

TNFRSF17 0 0.394524223201455 RF

SERPINA1 0 0.394599923622843 RF

GRB14 0 0.394752121705506 RF

TMEM220 0 0.395324328865324 RF

NMRAL2P 0 0.396775220151228 RF
