# Supplementary material for: Integration of single-cell and bulk RNA-seq via machine learning to reveal ferroptosis- and lipid metabolism-driven immune landscape heterogeneity and predict immunotherapy response in colon cancer
Source: Front Immunol. 2025 Dec 5;16:1699079. doi: 10.3389/fimmu.2025.1699079 (PMC12714941; doi:10.3389/fimmu.2025.1699079)
Supplement: Supplementary file 27 [file Table12.docx]

variable permutation dropout_loss label

TUBA1C 0 0.40234387419425 XGB

SEL1L3 0 0.402465204562941 XGB

BMP5 0 0.402533596373984 XGB

UCHL1 0 0.40257738528909 XGB

ADAM8 0 0.402720246461752 XGB

PTGDR2 0 0.402730328483421 XGB

RAB15 0 0.402792396675452 XGB

SPINK1 0 0.402868807181171 XGB

SEZ6L2 0 0.402886402360687 XGB

SLC38A5 0 0.403073678018487 XGB

SNCG 0 0.403185263839083 XGB

SERPINA1 0 0.403412769743325 XGB

FABP4 0 0.403429597027793 XGB

SHH 0 0.403585072913126 XGB

UGT2A3 0 0.403706817450074 XGB

KCNE3 0 0.404195320121525 XGB

ANXA3 0 0.404391273665713 XGB

LYPD8 0 0.404415105461352 XGB

EDN3 0 0.40471773822428 XGB

PPA1 0 0.404764173379134 XGB

ASPG 0 0.406040224936737 XGB

TMEM220 0 0.406088705806077 XGB

TNFRSF17 0 0.406310893147257 XGB

NOS2 0 0.406316533995059 XGB

CPA3 0 0.406581161184579 XGB

TMEM72 0 0.406872511028187 XGB

WDR72 0 0.407194421528183 XGB

CEACAM5 0 0.40812114089242 XGB

LINC00261 0 0.408794851931228 XGB

NMRAL2P 0 0.418413554960335 XGB
