# Supplementary material for: Integration of single-cell and bulk RNA-seq via machine learning to reveal ferroptosis- and lipid metabolism-driven immune landscape heterogeneity and predict immunotherapy response in colon cancer
Source: Front Immunol. 2025 Dec 5;16:1699079. doi: 10.3389/fimmu.2025.1699079 (PMC12714941; doi:10.3389/fimmu.2025.1699079)
Supplement: Supplementary file 28 [file Table13.docx]

Start: AIC=757.68

Surv(time, event) ~ GRB14 + CPA3 + NMRAL2P + UCHL1 + CDC25C +

SHH + FABP4 + DAPK1 + NEBL + SNCG + ANKRD22 + ADAM8 + TUBA1C +

SERPINA1 + ASPG + WDR72 + SEZ6L2 + ASPHD1 + TMEM220 + NOS2 +

EPOP + ANGPTL4 + LINC00261 + SLC38A5

Df AIC

- FABP4 1 755.70

- NEBL 1 755.78

- UCHL1 1 755.87

- SNCG 1 755.88

- SLC38A5 1 755.88

- ADAM8 1 755.96

- LINC00261 1 756.15

- ASPHD1 1 756.17

- SEZ6L2 1 756.40

- GRB14 1 756.57

- TMEM220 1 756.60

- NOS2 1 756.79

- TUBA1C 1 757.10

- SHH 1 757.19

<none> 757.68

- DAPK1 1 758.23

- SERPINA1 1 758.50

- CDC25C 1 758.91

- ANKRD22 1 759.13

- ANGPTL4 1 759.47

- EPOP 1 761.00

- ASPG 1 761.39

- CPA3 1 761.97

- WDR72 1 762.22

- NMRAL2P 1 767.92

Step: AIC=755.7

Surv(time, event) ~ GRB14 + CPA3 + NMRAL2P + UCHL1 + CDC25C +

SHH + DAPK1 + NEBL + SNCG + ANKRD22 + ADAM8 + TUBA1C + SERPINA1 +

ASPG + WDR72 + SEZ6L2 + ASPHD1 + TMEM220 + NOS2 + EPOP +

ANGPTL4 + LINC00261 + SLC38A5

Df AIC

- NEBL 1 753.79

- SLC38A5 1 753.90

- UCHL1 1 753.94

- SNCG 1 753.98

- ADAM8 1 753.98

- LINC00261 1 754.15

- ASPHD1 1 754.19

- SEZ6L2 1 754.40

- TMEM220 1 754.61

- GRB14 1 754.67

- NOS2 1 754.91

- TUBA1C 1 755.12

- SHH 1 755.19

<none> 755.70

- DAPK1 1 756.33

- SERPINA1 1 756.64

- CDC25C 1 756.97

- ANKRD22 1 757.16

+ FABP4 1 757.68

- ANGPTL4 1 758.12

- EPOP 1 759.03

- ASPG 1 759.54

- CPA3 1 759.98

- WDR72 1 760.23

- NMRAL2P 1 766.02

Step: AIC=753.79

Surv(time, event) ~ GRB14 + CPA3 + NMRAL2P + UCHL1 + CDC25C +

SHH + DAPK1 + SNCG + ANKRD22 + ADAM8 + TUBA1C + SERPINA1 +

ASPG + WDR72 + SEZ6L2 + ASPHD1 + TMEM220 + NOS2 + EPOP +

ANGPTL4 + LINC00261 + SLC38A5

Df AIC

- UCHL1 1 751.97

- SLC38A5 1 752.01

- SNCG 1 752.11

- ADAM8 1 752.13

- LINC00261 1 752.33

- ASPHD1 1 752.35

- SEZ6L2 1 752.47

- TMEM220 1 752.67

- GRB14 1 752.87

- NOS2 1 752.91

- TUBA1C 1 753.16

<none> 753.79

- SHH 1 753.99

- DAPK1 1 754.48

- SERPINA1 1 754.82

- CDC25C 1 755.20

- ANKRD22 1 755.61

+ NEBL 1 755.70

+ FABP4 1 755.78

- ANGPTL4 1 756.34

- EPOP 1 757.05

- ASPG 1 757.55

- CPA3 1 758.44

- WDR72 1 758.54

- NMRAL2P 1 764.02

Step: AIC=751.97

Surv(time, event) ~ GRB14 + CPA3 + NMRAL2P + CDC25C + SHH + DAPK1 +

SNCG + ANKRD22 + ADAM8 + TUBA1C + SERPINA1 + ASPG + WDR72 +

SEZ6L2 + ASPHD1 + TMEM220 + NOS2 + EPOP + ANGPTL4 + LINC00261 +

SLC38A5

Df AIC

- SLC38A5 1 750.20

- ADAM8 1 750.35

- LINC00261 1 750.46

- ASPHD1 1 750.50

- SNCG 1 750.52

- SEZ6L2 1 750.66

- TMEM220 1 750.75

- GRB14 1 751.05

- NOS2 1 751.23

- TUBA1C 1 751.39

<none> 751.97

- SHH 1 752.57

- DAPK1 1 752.60

- SERPINA1 1 753.21

- CDC25C 1 753.36

- ANKRD22 1 753.75

+ UCHL1 1 753.79

+ FABP4 1 753.92

+ NEBL 1 753.94

- ANGPTL4 1 754.64

- EPOP 1 755.66

- ASPG 1 756.12

- CPA3 1 756.59

- WDR72 1 756.92

- NMRAL2P 1 762.10

Step: AIC=750.2

Surv(time, event) ~ GRB14 + CPA3 + NMRAL2P + CDC25C + SHH + DAPK1 +

SNCG + ANKRD22 + ADAM8 + TUBA1C + SERPINA1 + ASPG + WDR72 +

SEZ6L2 + ASPHD1 + TMEM220 + NOS2 + EPOP + ANGPTL4 + LINC00261

Df AIC

- ASPHD1 1 748.62

- ADAM8 1 748.66

- LINC00261 1 748.71

- SNCG 1 748.85

- TMEM220 1 749.05

- SEZ6L2 1 749.06

- GRB14 1 749.33

- NOS2 1 749.46

- TUBA1C 1 749.89

<none> 750.20

- DAPK1 1 750.81

- SHH 1 751.11

- SERPINA1 1 751.43

- CDC25C 1 751.45

- ANKRD22 1 751.76

+ SLC38A5 1 751.97

+ UCHL1 1 752.01

+ NEBL 1 752.14

+ FABP4 1 752.16

- ANGPTL4 1 752.71

- EPOP 1 753.69

- ASPG 1 754.50

- CPA3 1 754.98

- WDR72 1 755.87

- NMRAL2P 1 761.47

Step: AIC=748.62

Surv(time, event) ~ GRB14 + CPA3 + NMRAL2P + CDC25C + SHH + DAPK1 +

SNCG + ANKRD22 + ADAM8 + TUBA1C + SERPINA1 + ASPG + WDR72 +

SEZ6L2 + TMEM220 + NOS2 + EPOP + ANGPTL4 + LINC00261

Df AIC

- LINC00261 1 747.13

- ADAM8 1 747.23

- TMEM220 1 747.36

- SNCG 1 747.41

- GRB14 1 747.80

- NOS2 1 747.87

- TUBA1C 1 748.51

<none> 748.62

- DAPK1 1 748.93

- CDC25C 1 749.75

- ANKRD22 1 749.87

- SHH 1 749.97

- SEZ6L2 1 750.07

- SERPINA1 1 750.10

+ ASPHD1 1 750.20

+ UCHL1 1 750.47

+ SLC38A5 1 750.50

+ NEBL 1 750.51

+ FABP4 1 750.59

- ANGPTL4 1 750.96

- EPOP 1 753.06

- ASPG 1 753.08

- CPA3 1 753.10

- WDR72 1 754.06

- NMRAL2P 1 759.94

Step: AIC=747.13

Surv(time, event) ~ GRB14 + CPA3 + NMRAL2P + CDC25C + SHH + DAPK1 +

SNCG + ANKRD22 + ADAM8 + TUBA1C + SERPINA1 + ASPG + WDR72 +

SEZ6L2 + TMEM220 + NOS2 + EPOP + ANGPTL4

Df AIC

- SNCG 1 746.05

- TMEM220 1 746.10

- NOS2 1 746.10

- ADAM8 1 746.33

- GRB14 1 746.70

- TUBA1C 1 746.80

- DAPK1 1 746.96

<none> 747.13

- ANKRD22 1 748.20

- SEZ6L2 1 748.35

+ LINC00261 1 748.62

+ ASPHD1 1 748.71

- CDC25C 1 748.79

+ NEBL 1 748.93

+ SLC38A5 1 748.99

+ UCHL1 1 749.03

- SHH 1 749.09

+ FABP4 1 749.12

- ANGPTL4 1 749.27

- EPOP 1 751.31

- CPA3 1 751.43

- SERPINA1 1 751.69

- ASPG 1 751.74

- WDR72 1 752.30

- NMRAL2P 1 760.87

Step: AIC=746.05

Surv(time, event) ~ GRB14 + CPA3 + NMRAL2P + CDC25C + SHH + DAPK1 +

ANKRD22 + ADAM8 + TUBA1C + SERPINA1 + ASPG + WDR72 + SEZ6L2 +

TMEM220 + NOS2 + EPOP + ANGPTL4

Df AIC

- TMEM220 1 744.89

- ADAM8 1 745.28

- NOS2 1 745.33

- TUBA1C 1 745.49

- GRB14 1 745.65

- DAPK1 1 745.80

<none> 746.05

- SEZ6L2 1 746.91

+ SNCG 1 747.13

+ LINC00261 1 747.41

+ ASPHD1 1 747.48

+ UCHL1 1 747.69

+ NEBL 1 747.78

+ SLC38A5 1 747.82

+ FABP4 1 747.82

- ANKRD22 1 748.07

- CDC25C 1 748.57

- SHH 1 748.64

- EPOP 1 749.90

- CPA3 1 749.92

- ANGPTL4 1 750.00

- ASPG 1 750.28

- WDR72 1 750.53

- SERPINA1 1 751.00

- NMRAL2P 1 760.19

Step: AIC=744.89

Surv(time, event) ~ GRB14 + CPA3 + NMRAL2P + CDC25C + SHH + DAPK1 +

ANKRD22 + ADAM8 + TUBA1C + SERPINA1 + ASPG + WDR72 + SEZ6L2 +

NOS2 + EPOP + ANGPTL4

Df AIC

- ADAM8 1 743.96

- GRB14 1 744.21

- DAPK1 1 744.27

- TUBA1C 1 744.28

- NOS2 1 744.38

<none> 744.89

- SEZ6L2 1 745.95

+ LINC00261 1 746.04

+ TMEM220 1 746.05

+ SNCG 1 746.10

+ ASPHD1 1 746.49

+ SLC38A5 1 746.58

+ NEBL 1 746.62

+ UCHL1 1 746.69

+ FABP4 1 746.76

- ANKRD22 1 747.28

- CDC25C 1 747.53

- SHH 1 747.79

- ANGPTL4 1 749.57

- WDR72 1 749.59

- EPOP 1 749.74

- SERPINA1 1 750.21

- ASPG 1 750.69

- CPA3 1 751.34

- NMRAL2P 1 759.49

Step: AIC=743.96

Surv(time, event) ~ GRB14 + CPA3 + NMRAL2P + CDC25C + SHH + DAPK1 +

ANKRD22 + TUBA1C + SERPINA1 + ASPG + WDR72 + SEZ6L2 + NOS2 +

EPOP + ANGPTL4

Df AIC

- TUBA1C 1 742.86

- GRB14 1 743.13

<none> 743.96

- NOS2 1 744.24

- DAPK1 1 744.49

+ LINC00261 1 744.50

- SEZ6L2 1 744.78

+ ADAM8 1 744.89

+ SNCG 1 745.14

+ TMEM220 1 745.28

+ ASPHD1 1 745.34

+ NEBL 1 745.41

+ SLC38A5 1 745.52

+ UCHL1 1 745.70

+ FABP4 1 745.87

- ANKRD22 1 747.25

- CDC25C 1 747.46

- SHH 1 747.76

- EPOP 1 748.43

- WDR72 1 748.48

- SERPINA1 1 749.13

- CPA3 1 749.40

- ASPG 1 749.43

- ANGPTL4 1 750.43

- NMRAL2P 1 758.06

Step: AIC=742.86

Surv(time, event) ~ GRB14 + CPA3 + NMRAL2P + CDC25C + SHH + DAPK1 +

ANKRD22 + SERPINA1 + ASPG + WDR72 + SEZ6L2 + NOS2 + EPOP +

ANGPTL4

Df AIC

- GRB14 1 742.30

<none> 742.86

- NOS2 1 743.22

- DAPK1 1 743.28

+ LINC00261 1 743.93

+ TUBA1C 1 743.96

+ ASPHD1 1 744.16

+ TMEM220 1 744.21

+ SNCG 1 744.21

+ ADAM8 1 744.28

+ SLC38A5 1 744.28

- SEZ6L2 1 744.41

+ NEBL 1 744.53

+ UCHL1 1 744.58

+ FABP4 1 744.77

- EPOP 1 746.49

- ANKRD22 1 746.66

- SHH 1 746.89

- WDR72 1 747.14

- SERPINA1 1 748.11

- ANGPTL4 1 748.64

- ASPG 1 748.88

- CPA3 1 749.17

- CDC25C 1 749.36

- NMRAL2P 1 758.87

Step: AIC=742.3

Surv(time, event) ~ CPA3 + NMRAL2P + CDC25C + SHH + DAPK1 + ANKRD22 +

SERPINA1 + ASPG + WDR72 + SEZ6L2 + NOS2 + EPOP + ANGPTL4

Df AIC

<none> 742.30

- NOS2 1 742.65

- DAPK1 1 742.77

+ GRB14 1 742.86

+ LINC00261 1 743.01

+ TUBA1C 1 743.13

+ ASPHD1 1 743.44

+ SNCG 1 743.62

+ SLC38A5 1 743.68

+ NEBL 1 743.76

+ ADAM8 1 743.84

+ TMEM220 1 743.90

+ UCHL1 1 743.99

+ FABP4 1 744.07

- SEZ6L2 1 744.16

- SHH 1 746.08

- ANKRD22 1 746.64

- SERPINA1 1 747.02

- WDR72 1 747.41

- EPOP 1 747.50

- ANGPTL4 1 747.89

- CDC25C 1 747.97

- CPA3 1 748.75

- ASPG 1 748.88

- NMRAL2P 1 759.49
